# Subgenome‐specific assembly of vitamin E biosynthesis genes and expression patterns during seed development provide insight into the evolution of oat genome

**DOI:** 10.1111/pbi.12571

**Published:** 2016-05-26

**Authors:** Juan J. Gutierrez‐Gonzalez, David F. Garvin

**Affiliations:** ^1^ Department of Agronomy and Plant GeneticsUniversity of MinnesotaSt. PaulMNUSA; ^2^ USDA‐ARS Plant Science Research UnitSt. PaulMNUSA

**Keywords:** oat homeologs, vitamin E, homeolog expression, oat evolution, seed composition, tocols

## Abstract

Vitamin E is essential for humans and thus must be a component of a healthy diet. Among the cereal grains, hexaploid oats (*Avena sativa* L.) have high vitamin E content. To date, no gene sequences in the vitamin E biosynthesis pathway have been reported for oats. Using deep sequencing and orthology‐guided assembly, coding sequences of genes for each step in vitamin E synthesis in oats were reconstructed, including resolution of the sequences of homeologs. Three homeologs, presumably representing each of the three oat subgenomes, were identified for the main steps of the pathway. Partial sequences, likely representing pseudogenes, were recovered in some instances as well. Pairwise comparisons among homeologs revealed that two of the three putative subgenome‐specific homeologs are almost identical for each gene. Synonymous substitution rates indicate the time of divergence of the two more similar subgenomes from the distinct one at 7.9–8.7 MYA, and a divergence between the similar subgenomes from a common ancestor 1.1 MYA. A new proposed evolutionary model for hexaploid oat formation is discussed. Homeolog‐specific gene expression was quantified during oat seed development and compared with vitamin E accumulation. Homeolog expression largely appears to be similar for most of genes; however, for some genes, homoeolog‐specific transcriptional bias was observed. The expression of *HPPD*, as well as certain homoeologs of *VTE2* and *VTE4*, is highly correlated with seed vitamin E accumulation. Our findings expand our understanding of oat genome evolution and will assist efforts to modify vitamin E content and composition in oats.

## Introduction

Cereal crops have experienced a nearly 2.2‐fold increase in production in the last 50 years (FAOSTATS, 2015). This is due both to the use of improved cultural practices and genetic enhancement, and to an increase in cultivated land area. However, this increased production is largely biased towards generally less nutritious but high‐yielding staple cereals such as rice, wheat and maize, at the expense of other more nutrient‐rich cereals including oats, barley, sorghum, rye and millet (DeFries *et al*., 2015). Thus, to meet global food demand, it will be critical to increase crop nutritional quality in addition to production. Developing cereals with enhanced nutritional content will require a deeper understanding of the metabolic pathways involved in the synthesis of phytonutrients and the factors that regulate them.

Compared to other foods, cereals contain only moderate amounts of vitamin E, but because cereals are a dietary staple in many regions around the world, they serve as an important source of this vitamin. Oats and barley have higher concentrations of vitamin E than other cereals (Gutierrez‐Gonzalez *et al*., 2013a; Panfili *et al*., 2003). However, due to the nature of commercial processing of barley, oat is likely to be a better source of vitamin E. Vitamin E and related compounds are potent antioxidants with free radical scavenging action. Most of the health benefits are associated with its strong antioxidant activity and include maintaining blood cholesterol levels, prevention of certain types of cancers and cardiovascular and neurological disorders, including cognitive function, and protection against Alzheimer's disease and other detrimental conditions (Kontush and Schekatolina, 2004; Ulatowski and Manor, 2015). Vitamin E is a blanket term that includes tocopherols (T) and tocotrienols (T3). Collectively, T and T3 are designated tocochromanols or tocols. There are four T analogues (forms): α−Τ, β−Τ, δ−T and γ−Τ, and four T3 analogues: α−Τ3, β−Τ3, δ−T3 and γ−T3, distinguished by the number and position of methyl groups in the chromanol ring. While α−T is the form with the highest vitamin E activity, other forms of vitamin E have higher, unique antioxidant and anti‐inflammatory properties against chronic diseases (Jiang, 2014). In oats, α−T3 is the predominant form of vitamin E, followed by α−T, β−T3 and β−T. Traces of δ and γ isoforms are also found (Gutierrez‐Gonzalez *et al*., 2013a; Peterson *et al*., 2007). Tocol accumulation profiles in developing oat seeds have only been studied recently (Gutierrez‐Gonzalez *et al*., 2013a). Herein, the terms tocochromanol, tocol and vitamin E form will be used interchangeably. Vitamin E, the synthesis of which has been intensively investigated in plants, plays a role in cell membrane stability, protection against lipid oxidation and stress tolerance.

All intermediate compounds and enzyme steps have been fully elucidated and are highly conserved among plant species (Mene‐Saffrane and DellaPenna, 2010). The vitamin E pathway is branched but also involves many of the same enzymes for the synthesis of both T and T3. For instance, the synthesis of all vitamin E compounds is initiated with GGDP. However, in the T branch, GGDP is reduced to PDP by GGR; while in the synthesis of T3, the initial step is the condensation of GGDP and HGA by HGGT, which is considered the committed step in tocotrienol biosynthesis. The committed step in the biosynthesis of tocopherols is the condensation of HGA and PDP, catalysed by VTE2. The last three enzymes, VTE1, VTE3 and VTE4, are common for the synthesis of both T and T3.

Several levels of ploidy are found in the oat genus *Avena*, with a base chromosome number of seven. The predominant cultivated oat (*Avena sativa* L) has been traditionally considered an allohexaploid with disomic inheritance (2*n* = 6*x* = 42). Its genome is designed ACD, reflecting the current belief that its 21 pairs of chromosomes originated from three distinct ancestral diploid genomes (A, C, and D), with the C genome more distinct from the others (Fu and Williams, 2008). However, because both the A and D hexaploid oat subgenomes have been shown to hybridize with A genome diploid species in genomic *in situ* hybridization experiments using fluorescent labels, it has been proposed that the A and D subgenomes evolved through autopolyploidy (Jellen *et al*., 1994). If this alternate hypothesis is true, the current nomenclature describing the oat genome does not accurately reflect its evolution (Ladizinsky, 2012). Tetraploid and diploid *Avena* species have been described (Rines *et al*., 2006), and current knowledge suggests that tetraploid genomes are CD, AC or AB. Notably, diploid A and C genome species are found in nature, but a diploid D genome donor has yet to be recognized. Further, results of hexaploid–diploid cross‐compatibility hybridizations do not support any recognized diploid A or C genome species as participants in the evolution of hexaploid oat species. Thus, considerable gaps in our understanding of oat evolution exist.

Improving vitamin E content in seeds is an important breeding objective for oats. Understanding homeolog‐specific sequence variation and differential expression is crucial to designing markers for key genes that regulate vitamin E accumulation that can be used to develop new improved varieties. Here, we elucidate the coding sequences of genes of the vitamin E pathway, including individual homeologs, in oats. Using this information, we estimate divergence times between the three oat subgenomes and characterize the relationship between homeolog‐specific expression profiles of these genes and accumulation profiles of vitamin E forms in developing oat seeds. Lastly, homeolog‐specific sequences for vitamin E pathway genes provide a unique opportunity to shed light on different scenarios that may have led to hexaploid oat evolution.

## Results

### Reconstruction of oat vitamin E pathway homeologs

We published the first oat seed transcriptome (Gutierrez‐Gonzalez *et al*., 2013b) and noted the high similarity among sequences presuming to represent the different homeologs. Due to low sequence divergence, the assembly process was likely to have comingled SNPs among homeologs, especially where low coverage prevented the assembler from achieving an accurate resolution. This commonly resulted in 5 or more transcript isoforms assembled for the same gene. Here, 1.1 m of Roche/454 reads averaging 457 bp were produced. These reads, together with the Illumina reads from the aforementioned oat seed transcriptome study, were used as a framework to reconstruct oat vitamin E pathway homeologs, through an *in silico* iterative procedure designed to recover oat homeolog‐specific consensus sequences from the orthologous sequences of the close relatives wheat, barley and Brachypodium (see Experimental Procedures). Reads were mapped to the consensus sequences and visually inspected to identify SNP polymorphisms that differentiate each specific homeolog for each catalytic step in the pathway (Figure S1). This identified 248 SNPs across all homeologs and enzymatic steps, most of which were biallelic. Only three of the SNPs were triallelic. A few indels were also identified, mostly in the UTRs (Figure S2). Primers were designed to target the homeolog‐specific SNPs, and PCR amplicons were Sanger‐sequenced and assembled for allele reconstruction.

We focused on the seven main catalytic reactions in vitamin E biosynthesis, which involve the enzymes GGR, HPPD, HGGT, VTE1, VTE2, VTE3 and VTE4. At least three distinct sequences were assembled for each gene; however, some of the sequences were not represented by complete cds (Figure S2). For VTE2 and GGR, five and four sequences, respectively, were identified; the additional sequences presumably reflect paralogs. When three putative homeologs were assembled and could be discriminated, we followed the convention of naming the most divergent one with the number 1 by adding the suffix ‘_1’ to the gene name. The two that are more similar to each other were termed with the suffixes ‘_2’ and ‘_3’; for instance, *HGGT_1*,* HGGT_2* and *HGGT_3*. We hypothesize that the most distinct homeolog (_1) originates from the C genome, and the other two more similar (_2 and _3) from the A and D genomes. To reflect each subgenome's loci, the following name convention was assigned: As_1, As_2 and As_3 to describe the grouping of all *A. sativa* homeolog_1, homeolog_2 and homeolog_3, respectively. For instance, As_1 includes the homeologous loci: *GGR_1*,* HPPD_1*,* HGGT_1*,* VTE1_1*,* VTE2_1*,* VTE3_1* and *VTE4_1*. Thus, As_1, As_2 and As_3 represent the three putative homeolog subgenomes. It is important to note that it is not possible to determine which homeolog belongs to either A or D subgenomes due to their high degree of similarity and the lack of chromosome anchoring. To test whether this is relevant to our study, particularly to the estimation of subgenome divergence times, homeologous sequences from the arbitrary As_2 and As_3 groups (A/D subgenomes) were shuffled between them and synonymous substitution rates computed within each group. Here, ‘A/D’ is used to indicate that the subgenome‐assigned homeologs could belong to either A or D subgenomes. Differences between all C versus A/D group comparisons were not significant, based on Student's *t*‐tests.

**Figure 1:**
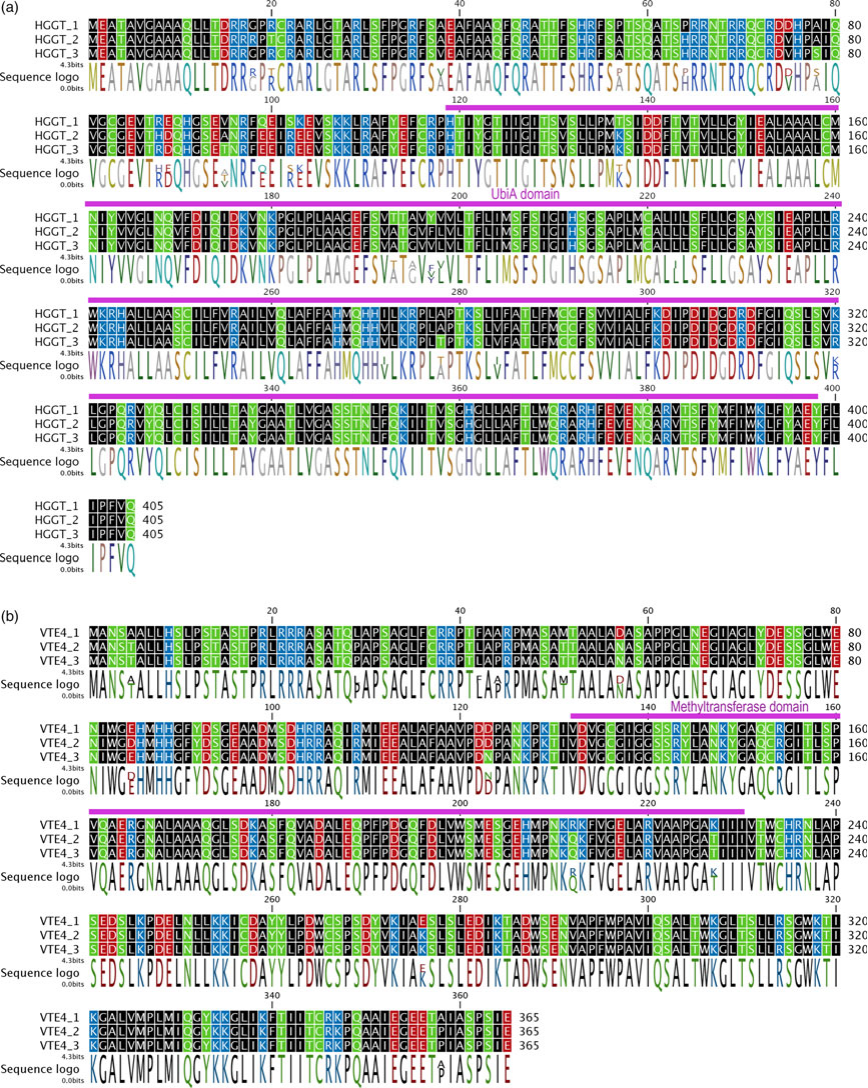
Examples of oat homeolog amino acid alignment of (a) HGGT and (b) VTE4. Residues colour‐coded by polarity: nonpolar side chains (black), polar (green), negative (−) electrical charge (red) and positive (+) electrical charge (blue). Purple bars on top of alignments represent main Pfam domains. Sequence logos colour‐coded using RasMol colours.

Overall, the nucleotide identity between C and A/D ranged from 95.5% to 98.2% (amino acid identity: 95.1–99.6%). Within the A/D genomes, the identities ranged between 98.6% and 99.8% (98.0%–100% aa identity). GGR was assembled into four distinct partial sequences, with the two largest having 380 aa in their coding sequences (cds), 82 aa shorter that the wheat and barley homologs. These two 380 aa‐long cds shared 98.9% similarity, and just 3 aa differences. All four assembled GGR partial sequences were not further considered for phylogenetic analyses because of the lack of enough length of overlapping fragments to provide reliable results. For HPPD, three partial homeologs were found, with lengths of 257, 251 and 248 aa. A 220‐ to 230‐bp‐long 3′‐UTR was also found. Three partial 421‐aa‐long VTE1 homeologs were assembled, covering 90% of the length of the wheat and barley orthologs, and 67 bp of the 3′‐UTR. Five different sequences were assembled for *VTE2*. However, two of them, *VTE2_4* and *VTE2_5*, are incomplete, and the partial sequenced fragments suggest the presence of premature stop codons, and therefore, they are presumably pseudogenes. Thus, they were not considered for phylogenetic analyses. Of the other three, one was assembled into a complete cds (398 aa), while the other two are 358 and 350 aa, respectively. An extended ~300 bp 3′‐UTR was also assembled. Three partial 301‐, 284‐ and 250‐aa‐long cds were assembled for VTE3, together with 140‐ to 210‐bp 3′‐UTR. Three complete cds for homeologs were assembled both for HGGT (405 aa) and VTE4 (361 aa) (Figure 1). Fragments of both 5′‐UTR and 3′‐UTR were also assembled. Subsequent phylogenetic analyses focused on HGGT, VTE1, VTE2, VTE3 and VTE4 because their homeologous cds were either complete or represented at least 85% of the predicted length, which permitted comparisons among different homeologs and robust inferences from the analyses.

### Phylogenetic analysis and evolution

**Figure 2:**
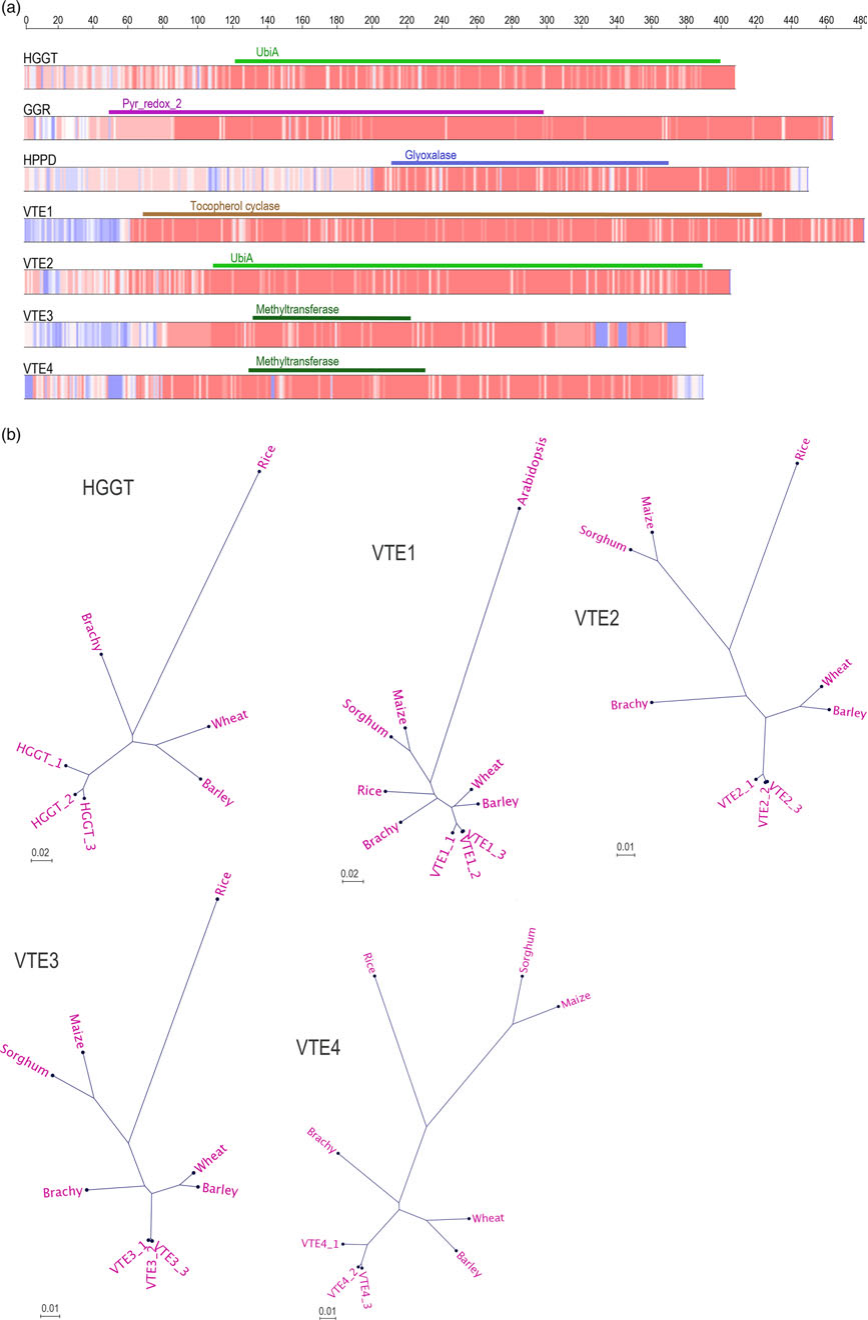
Amino acid conservation within principal enzymes in the vitamin E pathway. (a) Heat map of conserved regions showing areas with high (red) and low (blue) homology among the oat homeologous proteins and the amino acid sequences of other grass relatives: wheat, barley, Brachypodium, rice, maize and sorghum. Colour bars on top of alignments represent main Pfam domains. A rule at the top represents length in amino acids (b) Radial phylogenetic tree representation of the oat predicted homeologous proteins and those of grass relatives.

Nucleotide coding sequences of *HGGT*,* VTE1*,* VTE2*,* VTE3* and *VTE4* were aligned to homologous sequences from barley, wheat, Brachypodium, rice, sorghum, maize and Arabidopsis to generate the corresponding phylogenetic trees (Figure S3). Translated homeologous oat cds were also aligned to the same sequences (Figure S4). Nucleotide and aa alignments show that similarity between oat homeologs is higher than to homologs from the other species (Figure 2). The alignments also illustrate a high degree of similarity across all of the species, which is consistent with the fundamental importance of vitamin E in plants. The high homology is particularly evident in the regions containing the predicted main structural domains (Figure 2a). The phylogenetic trees of the predicted proteins are displayed in Figure 2b.

The number of synonymous nucleotide substitutions per synonymous site (*Ks*) was used to estimate divergence times between *A. sativa* homeologs (Table 1). Pairwise comparisons were performed for *HGGT*,* VTE1*,* VTE2*,* VTE3* and *VTE4*. *Ks* was computed by two approximate and one maximum‐likelihood (ML) models. For each model, *Ks* was reported as the average value of the pairwise comparisons. *Ks* values were also calculated between reference genomes and included as quality controls to test our molecular clock. Even though ML estimates are known to be often biased in small samples (Yang and Nielsen, 2000), the three models gave similar divergence times for most of the pairwise comparisons. Divergence times were calculated with averaged *Ks* values and assuming a molecular clock of 6.1 × 10^−9^ synonymous substitutions per synonymous site per year (TIBI, 2010). Results suggest that A/D diverged from C between 7.9 and 8.7 MYA and that A/D diverged from a common ancestor 1.1 MYA. Furthermore, pairwise synonymous substitution rates between *A. sativa* vitamin E pathway homeologs and orthologs from other grass genomes were also determined (Table 1). For instance, the times of divergence between oats and members of the Triticeae (wheat, barley) were estimated to be 25.5–26.5 MYA between oats and wheat, and 23–25 MYA between oats and barley. Synonymous substitution values also suggest that *A. sativa* and Brachypodium diverged approximately 29–30 MYA.

**Table 1:** Synonymous substitution rates and divergence times between vitamin E biosynthesis genes and between oat homeologs and putative orthologs from other grass species

Genome pairs	aKs	MYA	bKs	MYA	cKs	MYA
A. sativa_3–A. sativa_2	0.0135	1.1	0.0137	1.1	0.0130	1.1
A. sativa_1–A. sativa_2	0.1019	8.4	0.0964	7.9	0.1003	8.2
A. sativa_1–A. sativa_3	0.1056	8.7	0.1002	8.2	0.1038	8.5
Wheat–Barley	0.1479	12.1	0.1547	12.7	0.1523	12.5
Barley–A. sativa_2	0.3040	24.9	0.2940	24.1	0.2981	24.4
Barley–A. sativa_3	0.3034	24.9	0.2944	24.1	0.2983	24.5
Barley–A. sativa_1	0.2855	23.4	0.2811	23.0	0.2849	23.4
Wheat–A. sativa_2	0.3228	26.5	0.3173	26.0	0.3109	25.5
Wheat–A. sativa_3	0.3221	26.4	0.3173	26.0	0.3112	25.5
Wheat–A. sativa_1	0.3148	25.8	0.3096	25.4	0.3098	25.4
Brachy–A. sativa_2	0.3561	29.2	0.3620	29.7	0.3747	30.7
Brachy–A. sativa_3	0.3657	30.0	0.3711	30.4	0.3815	31.3
Brachy–A. sativa_1	0.3667	30.1	0.3684	30.2	0.3802	31.2
Brachy–Barley	0.3760	30.8	0.3787	31.0	0.3789	31.1
Brachy–Wheat	0.3805	31.2	0.3893	31.9	0.3866	31.7
Rice–A. sativa_2	0.5859	48.0	0.6530	53.5	0.5964	48.9
Rice–A. sativa_3	0.5851	48.0	0.6498	53.3	0.5943	48.7
Rice–A. sativa_1	0.5745	47.1	0.6397	52.4	0.5914	48.5
Rice–Barley	0.6205	50.9	0.7048	57.8	0.6615	54.2
Rice–Wheat	0.5742	47.1	0.6465	53.0	0.5951	48.8
Rice–Brachy	0.5905	48.4	0.6352	52.1	0.6071	49.8
Maize–A. sativa_2	0.7074	58.0	0.7137	58.5	0.6862	56.2
Maize–A. sativa_3	0.6939	56.9	0.7085	58.1	0.6856	56.2
Maize–A. sativa_1	0.7168	58.8	0.7200	59.0	0.6992	57.3
Maize–Barley	0.7531	61.7	0.7524	61.7	0.7175	58.8
Maize–Wheat	0.6814	55.8	0.6400	52.5	0.6221	51.0
Maize–Brachy	0.8462	69.4	0.7091	58.1	0.7087	58.1
Maize–Rice	0.6886	56.4	0.7318	60.0	0.7259	59.5
Sorghum–A. sativa_2	0.7630	62.5	0.6377	52.3	0.7064	57.9
Sorghum–A. sativa_3	0.7840	64.3	0.6496	53.2	0.7208	59.1
Sorghum–A. sativa_1	0.7330	60.1	0.6432	52.7	0.7151	58.6
Sorghum–Barley	0.7703	63.1	0.6715	55.0	0.7278	59.7
Sorghum–Wheat	0.6988	57.3	0.5827	47.8	0.6434	52.7
Sorghum–Brachy	0.6946	56.9	0.6076	49.8	0.6476	53.1
Sorghum–Rice	0.5821	47.7	0.6642	54.4	0.6509	53.4
Sorghum–Maize	0.1660	13.6	0.1771	14.5	0.1610	13.2

MYA: million years ago. Times calculated assuming λ = 6.1 × 10^−9^.

a Method of Yang and Nielsen (2000).

b Method of Nei and Gojobori (1986).

c Maximum‐likelihood (ML) method (Goldman and Yang, 1994).

### Homeolog‐specific expression analysis

Homeolog expression in developing seeds was calculated as normalized absolute fragment counts as described in the Methods section. Several tests were run to quality‐control the expression values among replications. First, relationships between samples (replicates at each time point) were inspected using a multidimensional scaling plot (MDS) (Figure S5). Distances on the plot approximate the expression differences between the samples. The plot shows that replicated samples which belong to the same developmental stage tend to group together. Second, a principal component analysis (PCA) was computed on the first two PCs (Figure S6). This revealed that the first two PCs captured 95.8% of the variance in the data. The first PC (PC1) explained 89.4% of the variance, and PC2 6.4%. The PCA also shows that replicates of each developmental stage tend to group together. Third, a heat map of distance matrix was computed to examine similarities and dissimilarities among the experimental factors (developmental stages) (Figure S7). It is clear from this analysis that samples are more similar between replicates in the same stage than they are among distinct stages, in agreement with the experimental design.

**Figure 3:**
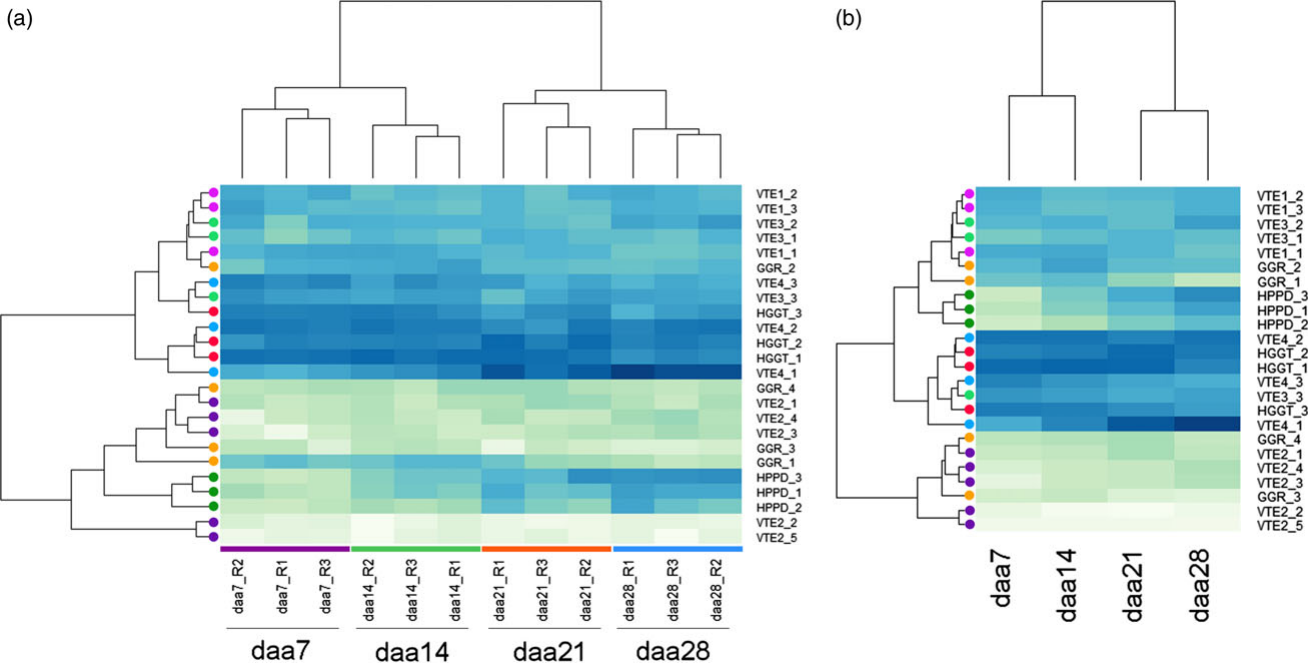
Developmental expression profiles of vitamin E biosynthesis genes in oat seeds. Heat maps show variance stabilization transformed homeolog normalized expression count values for (a) each individual replicate and (b) average of the three replicates per stage. Samples taken at the grain developmental stages 7, 14, 21 and 28 days after anthesis: daa7, daa14, daa21 and daa28, respectively. Replications are noted with the suffixes R1, R2 and R3. Darker blue colour implies higher expression. Circles show homeologs colour‐coded by gene for easier identification.

Transformed and normalized replicated homeolog‐specific expression count values were visualized with the help of a heat map (Figure 3). Clustering shows a replicate‐by‐stage grouping of samples according to developmental stage, as expected (Figure 3a), indicating that expression values are consistent between replicates of the same developmental stage. Sample clustering also shows that changes in expression of vitamin E biosynthetic genes occur progressively during seed development and maturation, in agreement with vitamin E accumulation profiles during development (Gutierrez‐Gonzalez *et al*., 2013a). To facilitate visual inspection of expression profiles, replicated counts were also averaged by developmental stage and clustered (Figure 3b). In addition, to visually identify transcripts that increase or decrease concomitantly with time, expression levels were also standardized to the mean and clustered (Figure S8).

**Figure 4:**
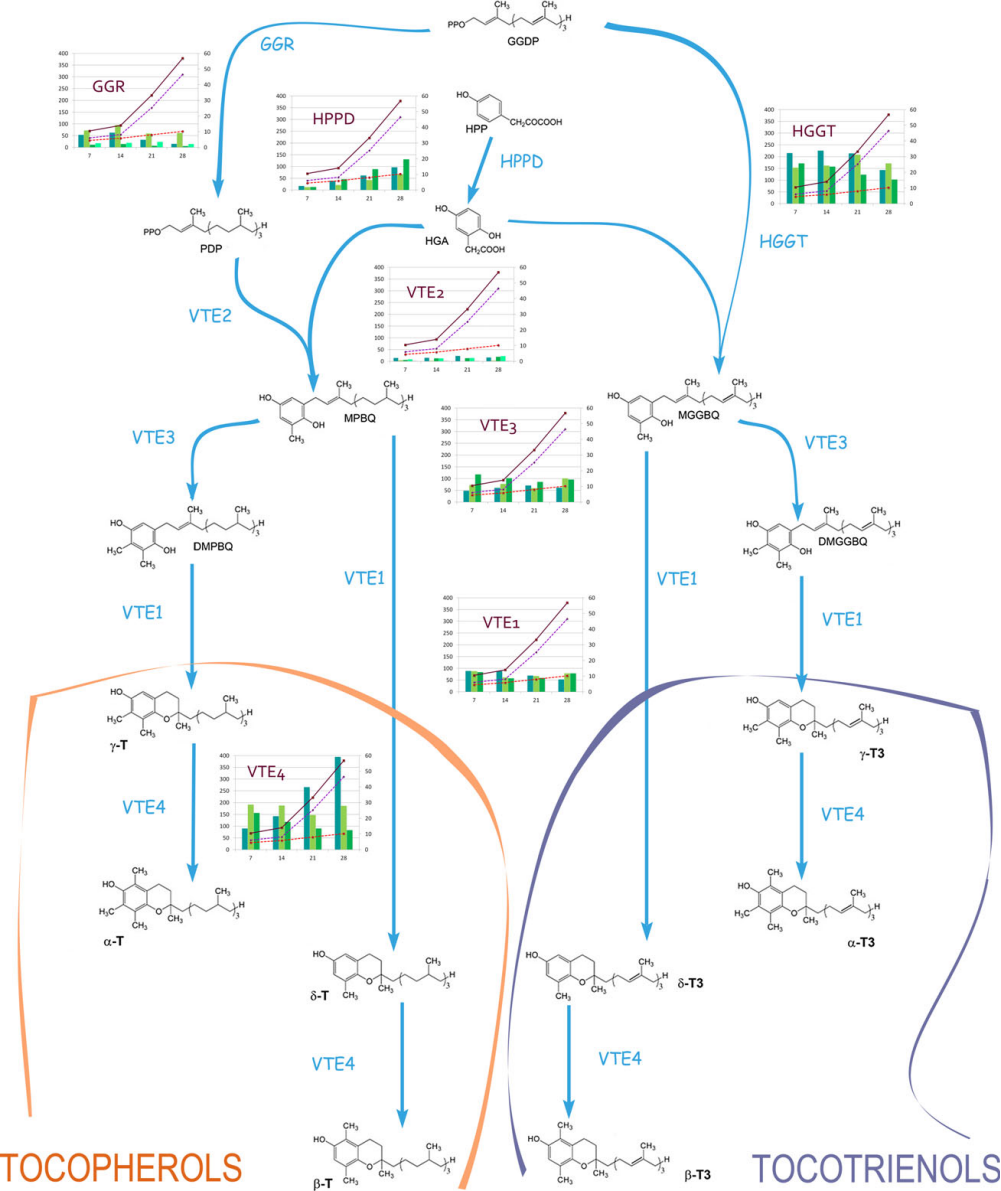
Expression of oat gene homeologs comprising vitamin E biosynthesis in developing oat seeds. Each enzymatic step is linked to a graph. Each column in the graph represents the expression of a homeolog form, gene_1, gene _2 and gene_3 for the first, second and third homeologs, respectively, and grouped by developmental stage (*x*‐axis). *GGR* has a fourth column for *GGR_4* and *VTE2* two extra columns (*VTE2_4* and *VTE2_5*). Lines in the graph represent tocopherol (red dashed line), tocotrienol (purple dashed line) and total tocol accumulation (brown solid liner) values throughout seed development, as reported by Gutierrez‐Gonzalez *et al*. (2013a). Values on the *y*‐axis refer to absolute counts (left axis) and μg/g (right). DMGGBQ: 2,3‐dimethyl‐5‐geranylgeranylbenzoquinol; DMPBQ: 2,3‐dimethyl‐6‐phytyl‐1,4‐benzoquinone; GGDP: geranylgeranyl diphosphate; GGR: geranylgeranyl diphosphate reductase; HGA: homogentisic acid; HPP: p‐hydroxyphenylpyruvic acid; HPT: homogentisate phytyltransferase; HPPD: 4‐hydroxyphenylpyruvate dioxygenase; HGGT: homogentisate geranylgeranyl transferase; MGGBQ: 2‐methyl‐6‐geranylgeranylbenzoquinol; MPBQ: 2‐methyl‐6‐phytylbenzoquinol; PDP: phytyl‐diphosphate; VTE1: 2‐methyl‐6‐phytyl‐1,4‐benzoquinone cyclase; VTE2: homogentisate phytyltransferase; VTE3: 2‐methyl‐6‐phytyl‐1,4‐benzoquinone/2‐methyl‐6‐solanyl‐1,4‐benzoquinone methyltransferase; VTE4: tocopherol methyltransferase; T: tocopherol; T3: tocotrienol.

It is of interest to compare homeolog expression values with the patterns of accumulation for vitamin E forms at each seed developmental stage (Table S1). Table S2 shows the Pearson product‐moment correlation coefficients among homeolog expression counts and vitamin E forms alongside seed development. The three HPPD homeologs are highly correlated (*r* = 0.97–1.0) with T, T3 and total vitamin E forms. VTE2_3 and VTE2_4 (a putative pseudogene), but not the other three VTE2 homologs, show also a high correlation (*r* = 0.89–0.96) with T, and total vitamin E forms, and slightly less with T3. Finally, the last homeolog that shows a high correlation with vitamin E forms is VTE4_1 (*r* = 0.99 with T3 and total; and *r* = 1.0 with T). Interestingly, the other two VTE4 homeologs have negative correlation coefficients, in the range of −0.94 to −0.22. This information is summarized in Figure 4, which employs vitamin E accumulation data over time in developing seeds from an earlier study (Gutierrez‐Gonzalez *et al*., 2013a) with patterns of homeolog expression at each catalytic step of the vitamin E pathway.

## Discussion

Gene sequences in oats are difficult to dissect due to the hexaploid nature of the oat genome, with three constituent subgenomes, and genomic studies in oats have been hindered by the high similarity between these subgenomes. In this study, we took advantage of the fact that the vitamin E pathway is highly conserved in plants to sequence oat genes coding for the main vitamin E biosynthesis enzymes. Three homeologs were assembled for all genes considered, except for *VTE2* and *GGR*, where five and four were found, respectively. For two of the *VTE2* copies, the presence of early stop codons is suggested, although this could not be unequivocally contrasted because the sequences lack part of the 5′ region. For *GGR*, no homeolog could be assembled in full and thus it was not possible to elucidate the presence of early stop codons. Clusters of paralogous genes are common in plants. Examples include disease‐resistant gene clusters and usually arise by gene duplication and unequal crossing over, which may also be the case here.

Our multigene comparison corroborates previous evidence that hexaploid oat possesses two highly similar subgenomes and a more divergent one (Fu and Williams, 2008; Gutierrez‐Gonzalez and Garvin, 2011; Oliver *et al*., 2013). The three constituent oat subgenomes differed in the level of polymorphism observed. The amount of polymorphism between two of the homeologs from the more similar subgenomes was always significantly lower for all of the vitamin E genes than levels of polymorphism with the third subgenome homeolog. Under the nomenclature employed, As_2 and As_3 are the homeolog subgenomes with higher similarity and presumably correspond to the A/D subgenomes, with the degree of homology reaching 98.0%–100% amino acid identity.

The molecular clock used in our computations has given pairwise divergence times between grass genomes that are consistent with previous studies on species diversification. For instance, the wheat–barley divergence time of 12.1–12.7 MYA is within the range of previous multigene estimates (11.6 ± 2.4) (Chalupska *et al*., 2008), and the 30.8–31.9 MYA time of divergence of Brachypodium from the Triticeae (wheat and barley) is also very similar to the 31 MYA estimated by Abrouk *et al*. (2010), and slightly lower than the 32–39 MYA reported elsewhere (The International Brachypodium Initiative (TIBI), 2010). Finally, the sorghum–Brachypodium divergence date estimated to be 49.8–56.9 MYA is similar to the 45–60 MYA previously calculated (The International Brachypodium Initiative (TIBI), 2010). Thus, evolutionary divergence models computed employing as sequences genes involved in vitamin E synthesis are similar to those previously reported from other gene sets and sequenced genomes, despite the smaller number of loci considered and the small number of homeolog‐specific synonymous substitutions found in some pairwise comparisons.

According to pairwise comparisons with vitamin E pathway homeologs, the divergence between *A. sativa* and Brachypodium occurred approximately 29–30 MYA (Table 1). The estimated time of divergence of *A. sativa* from the Triticeae is in the range of 23–26 MYA, which is consistent with the predominant belief that barley/wheat and oats diverged from each other after they diverged from Brachypodium (Catalan *et al*., 1997). Our pairwise comparisons posit the time of divergence A/D from C at 7.9–8.7 MYA. The molecular clock also indicates that A/D diverged from a common ancestor approximately 1.1 MYA.

Two long‐time proposed evolutionary models can accommodate our results and the accumulated evidence to explain how the constituent subgenomes may have arisen: (i) an autotetraploid origin of the two more similar subgenomes As_2 and As_3 (A/D), and subsequent hybridization with a C genome (Badaeva *et al*., 2010; Ladizinsky, 2012); and (ii) an allotetraploid formation with two close A genome species and later subgenome divergence (Fu and Williams, 2008; Ladizinsky, 1998, 2012). Nevertheless, to date, none of them have been unequivocally substantiated as participating in the formation of the hexaploid species. While speculative, a third potential evolutionary model to consider involves homoploid hybrid speciation (HHS), hybrid speciation without change in ploidy level. This model would have occurred between two similar ancestral A genome diploid *Avena* species 1.1 MYA, to form the D genome. HHS would have been preceded by an allopolyploid event to form the ancient AC tetraploid, which occurred earlier than our estimated divergence date of 7.9–8.7 MYA. Subsequently, after HHS formed the D genome, a second allopolyploidization event between the AC tetraploid and the D genome homoploid produced hexaploid *Avena sativa*. Each polyploid might have also undergone additional rearrangement during both tetraploidy and/or after hexaploid hybrid speciations, including gain or loss of parental segments, gene suppression and activation, structural rearrangements and epigenetic modifications, often observed in polyploids of the genus *Avena* (Jellen *et al*., 1994; Sanz *et al*., 2010; Yang *et al*., 1999). These structural and functional modifications, which could also have taken place following HHS as well as in the other proposed models after a polyploid has formed, are presumably required to stabilize duplicated genomes and permit fertility in the nascent polyploid. The HHS model may explain the high similarity between A and D hexaploid subgenomes (Linares *et al*., 1998), and the fact that a diploid D genome species has not been identified in nature. It is also possible that, because none of the existing diploid A genome species has been recognized to be part of the original hexaploid oat subgenomes, a second homoploid hybridization event between two ancient A genome diploid *Avena* species to form the actual A hexaploid subgenome could have also taken place. This second HHS event would have involved two or more of the A genome *Avena* species that up to now have been proposed to have donated the A hexaploid subgenome: *A. strigosa*,* A. canariensis* and *A. wiestii* (Fu and Williams, 2008; Li *et al*., 2000; Rajhathy and Thomas, 1974). Recent empirical studies have pointed to HHS as a relatively common phenomenon (Schumer *et al*., 2014). Indeed, in wheat, recent research significantly revises the evolution of this species, with the A and B genomes giving rise to the D genome through HHS. Thus, the wheat genome is a product of successive rounds of homoploid and polyploid hybrid speciation (Marcussen *et al*., 2014). Frequently, HHS is proposed from limited genetic/genomic data, which is the case here. Thus, more genome sequence information will be needed to explore this evolutionary scenario.

It is important to note that only one oat genotype was considered for this study. Haplotype differences in oat could be important and may lead to overestimation of the time of polyploid formation. However, the comparable divergence times obtained in our pairwise comparisons of other species validate our molecular clock calculations and the proposed divergence time for the oat subgenomes. Equally important, as our study was focused in just one metabolic pathway, it will be valuable to compare our results to those obtained both from gene families for other pathways as well as with whole subgenome assemblies, when that information becomes available.

Tissue‐specific silencing can occur after polyploidization as part of gene subfunctionalization (Adams *et al*., 2003). Because at least three homeologs per gene were assembled using cDNA reads, our data suggest that this is not the case for vitamin E gene expression in developing seeds. In the majority of the genes studied, individual homeologs appear to be expressed nearly equally in seed tissue. However, *VTE2* and *VTE4* diverge from this pattern; the number of transcripts of *VTE2_1* and *VTE2_3* is seven times more abundant than those of *VTE2_2*, and it does not appear to be affected by the seed developmental stage. For *VTE4*,* VTE4_1* is expressed at the highest level, and its expression is highly correlated with T (*r* = 1), T3 (*r* = 0.99) and total vitamin E content (*r* = 0.99). Expression of *VTE4* in transgenic Arabidopsis did not increase total tocols in seeds but instead modified tocol composition from practically all γ‐tocopherol, the predominant vitamin E form in Arabidopsis, into α‐tocopherol (Van Eenennaam *et al*., 2003). Because VTE4 catalyses the conversion of γ and δ forms of both T and T3 into α and β forms, the elevated levels of VTE4_1 transcripts are consistent with the relative abundance of the forms of vitamin E in oat seeds: α‐T3 > αT > βT3 > βT (Gutierrez‐Gonzalez *et al*., 2013a). Unequal contributions from homeologs in polyploids have been previously shown in wheat (Akhunova *et al*., 2010; Zhang *et al*., 2014), cotton (Adams *et al*., 2003) and rapeseed (Xu *et al*., 2009) and are commonly due to mutations in regulatory regions or epigenetic changes in both methylation patterns and histone modifications. In addition to VTE4_1, the other enzyme with homeologs exhibiting a high correlation (*r* = 0.97–1) with T, T3 and total vitamin E forms is HPPD. In contrast to *VTE4*, whose *VTE4_1* homeolog was expressed at a much higher rate than the other two, all three *HPPD* homeologs appear to be expressed similarly. Interestingly, HPPD and VTE4 catalyse the first and last shared reactions in the synthesis of both tocopherols and tocotrienols, respectively, and thus appear to be key points in the regulation of metabolic flux through the vitamin E pathway.

The committed steps in the vitamin E biosynthetic pathway have been traditionally considered the condensation of HGA and PDP to form MPBQ, the intermediate of all tocopherols, and the condensation of HGA and GGPP to form MGGBQ, the intermediate of all tocotrienols. Both reactions are catalysed by VTE2 and HGGT, respectively. All *HGGT* and *VTE2* homeologs, except for *VTE2_*2, appear to be expressed in a similar fashion and at constant levels throughout oat grain maturation (Figure 4, Table S2). However, basal levels of each of the *HGGT* homeologs transcripts are on average 15‐fold higher than those of *VTE2*, consistent with the higher presence of tocotrienols than tocopherols. Thus, what appears to direct metabolic flux towards tocotrienols rather than tocopherols in oat seeds is the increased *HGGT*/*VTE2* expression ratio. Overall, the differences in the amino acid composition within homeologs do not suggest variable catalytic activities for them. Instead, differences in metabolite synthesis appear to be largely due to changes in transcript abundance produced by changes in the regulatory elements.

Seeds are the plant organ that accumulates more distinct vitamin E forms than other organs and in greater amounts (DellaPenna, 2005). Thus, examining the expression of genes associated with vitamin E accumulation in seeds provides a powerful opportunity to explore the relationship between gene expression and metabolite accumulation. This study not only provides a comprehensive view of the expression of genes coding for all of the steps of vitamin E biosynthesis but provides new insight into how their expression ultimately leads to the observed composition of vitamin E forms in oat seeds. Our successful unravelling of individual vitamin E homeolog sequences will allow an exploration of natural variation for vitamin E content and composition in oats, and its causal basis. Our study paves the way for future research on other pathways involved in the synthesis of health‐promoting compounds in oat seeds, such as those that lead to the accumulation of β‐glucan and avenanthramides. Analysis of homeolog expression in these pathways, in a manner similar to the current study, may identify particular targets for modification, to improve nutritional attributes of oat seeds.

## Experimental procedures

### Plant materials, growth conditions, RNA extraction and Illumina sequencing

Oat genotype Ogle‐C, derived from a single plant reselection with several rounds of selfing from the cultivar ‘Ogle’, was used for this study. Plant growth, tissue harvesting, RNA extraction and Illumina sequencing were reported in Gutierrez‐Gonzalez *et al*. (2013b). However, in this previous analysis to develop an oat seed transcriptome, reads were pooled across replicates and developmental stages, while in this study, reads were not pooled but instead were independently analysed by replicate. Thus, a total of 12 independent non‐normalized libraries were constructed, one for each developmental stage (developing seeds harvested 7, 14, 21 and 28 days after anthesis (daa)) and replicate. With the help of Prinseq (Schmieder and Edwards, 2011) and custom scripts, the raw reads were cleaned of primer adaptors, low‐quality reads, reads with nonidentified bases and 3′‐trimmed according their individual base‐call quality score (QS), maintaining only the bases with a QS above 20 and at least 90 bases long. For the paired‐end reads in which just one read passed all quality filters, that read was maintained as a singleton. After the cleaning steps, a total of 166 m reads with average QS of 34 remained distributed as follows: for 7‐daa, 40.4 m (80.7% paired and 19.3% singletons), with an average of 13.5 m per replicate; for 14‐daa, 41.0 m (79.1% paired and 20.9% singletons), with an average of 13.7 m per replicate; for 21‐daa, 34.3 m (84.9% paired and 15.1% singletons), with an average of 11.4 m per replicate; lastly, for 28‐daa, 50.9 m (78.2% paired and 21.8% singletons), with an average of 17.0 m per replicate.

### Roche sequencing

Equal amounts of RNA from each stage and replicate were pooled and used for normalized cDNA library preparation at the University of Illinois Keck Center, using a custom in‐house protocol developed specifically for Roche/454 sequencing. Quality control of the completed library sample included Invitrogen Qubit concentration and quality evaluation on an Agilent DNA7500 chip. The sample was processed from emPCR through sequencing following the latest Roche emPCR Method Manuals, and run on the Roche GS FLX+ system version 2.9, flow pattern A and analysed through signal processing using Roche software version 2.9. Quality filtering of the reads included tag removal with TagCleaner (Schmieder *et al*., 2010), elimination of duplicates, trimming 3′ bases with quality less than Q20 and removal of reads with undefined bases with the help of Prinseq (Schmieder and Edwards, 2011). Reads were retained if they were at least 90 nt long and Q25 on average, and had no undefined bases. After quality control, a total of 1.1 m reads ranging from 90 to 861 bp (457 bp on average) remained.

### Sanger sequencing

A pool of equal amounts of RNA from all sampled seed developmental stages and replications was used for reverse transcription with Superscript III transcriptase (Life Technologies, Grand Island, NY, USA), following the manufacturer's recommendations. Sequencing primers were selected to target homeolog‐specific SNPs. PCR reactions were conducted using Q5^®^ High‐Fidelity DNA polymerase (New England BioLabs, Ipswich, MA) following the vendor's protocol. Optimal primer annealing temperatures were calculated with NEB Tm Calculator v1.7.3 (www. http://tmcalculator.neb.com). PCR amplicons were sequenced in both directions using the Sanger dideoxy terminator method in an ABI PRISM^™^ 3730xl DNA Analyzer (Applied Biosystems, Foster City, CA, USA), with the ABI PRISM^®^ BigDye^®^ Terminator v3.1 Cycle Sequencing Kit chemistry. Assays were conducted in the Biomedical Genomics Center, University of Minnesota. Bidirectional sequenced amplicons were visually inspected, quality checked and assembled with the help of Mega v6.06 (Tamura *et al*., 2013).

### Orthology‐guided gene assembly

To reconstruct oat homeolog coding sequences, an iterative approach was employed. As the vitamin E pathway is highly conserved in plants, putative orthologous sequences from related grass species (barley, wheat and Brachypodium) were used as starting templates. Illumina and Roche reads with homology to known vitamin E pathway genes were filtered out (BLASTN, e‐value 1e‐3) and used in subsequent steps. Reads were then mapped to the orthologous sequences with relaxed parameters (minimum fraction of total alignment length that must match the reference sequence 0.75, and minimum identity fraction between the aligned region of the read and the reference sequence 0.75), to produce a consensus sequence that was used in the next alignment cycle. Reads with more than one match with the same score were randomly assigned. The process of read mapping and attaining the consensus sequence was repeated until no change in the consensus sequence was observed. Mapped reads were checked for candidate homoeolog SNPs by visual inspection. These SNPs were used to design homeolog‐specific primers for PCR reactions and subsequent Sanger sequencing of amplicons. Sequences have been deposited in GenBank (accession numbers: JZ917187 ‐ JZ917210).

### Expression analysis

To quantify gene expression, paired reads and singletons were mapped to the assembled vitamin E pathway homeologs using CLC Genomics Workbench 8.0.2 (CLCGW8) (www.clbio.com), with zero‐tolerance parameters: minimum fraction length and minimum identity fraction of 1. Fragments were counted as follows: when both reads in a pair successfully mapped, only one fragment was considered; singletons were considered as single fragments; broken pairs, where just one read of the pair mapped, were not counted. When a paired read or singleton could be matched to more than one position, it was randomly assigned to one of these positions. This random distribution was proportional to the number of unique matches that each transcript has, normalized by the transcript length. Using these stringent parameters, 75.6% of the reads mapped to a unique location. Total fragment counts were normalized across the 12 samples (four developmental stages × three replicates) using the scaling normalization method provided in the DESeq package of R and described in (Anders and Huber, 2010). For graphical representations, normalized counts were variance‐stabilizing transformed (Tibshirani, 1988). Principal component analysis (PCA) was computed with the R package ggbiplot, which projects the data on the first two PCs. Multidimensional scaling plot (MDS) was calculated using the R package edgeR to show the relationships between all pairs of samples. Heat maps were produced with DEGseq using variance stabilization transformed counts.

### Sequence alignments, divergence times and phylogeny

Nucleotide and amino acid multiple alignments were performed using both a modified ClustalW algorithm implemented in Vector NTI Advance v.11.5.4 software (Life Technologies) and the progressive alignment algorithm implemented in CLCGW8, and the best alignment was chosen after visual inspection. A maximum‐likelihood phylogenetic tree based on expectation maximization on substitution model parameters and branch length optimization (PHYML) was used to construct the protein and nucleotide phylogeny trees. The UPGMA algorithm, which assumes a constant rate of evolution, was used to obtain a starting tree. For nucleotide sequence phylogenetic trees, five substitution models were tested; HKY with variable substitution rates Hasegawa *et al*., 1985) usually produced the best statistics and was therefore employed. For protein substitution model, WAG model (Whelan and Goldman, 2001) was used. A bootstrap analysis with 1000 replicates was performed to evaluate the reliability of the trees. Branch lengths are given in terms of expected numbers of substitutions per nucleotide site.

Pairwise nucleotide synonymous substitution rates (*Ks*) were calculated by three methods: (i) Yang and Nielsen (2000) method, which takes into account transition/transversion rate bias and base/codon frequency bias; (ii) Nei and Gojobori (1986) method, a codon‐based model that corrects for multiple substitutions allowing transition/transversion rate bias and codon usage bias; and (iii) a maximum‐likelihood method (ML) (Goldman and Yang, 1994). For the first two, the *yn00* program of the PAML package v. 4.8a (Yang, 2007) was used, while *codeml* of the same package was used for ML. All positions with gaps were excluded from the calculations. Divergence times were calculated based on a molecular clock of 6.1 × 10^−9^ synonymous substitutions per synonymous site per year (The International Brachypodium Initiative (TIBI), 2010). Vitamin E pathway sequences from other species used in these analyses are listed in Table S3.

## Conflict of interest

The authors declare that they have no conflict of interests.

## Supporting information


**Figure S1**. Capture of reads mapped to reference barley vitamin E gene.

**Figure S2**. Nucleotide and amino‐acid alignment of oat homeologs.

**Figure S3**. Maximum likelihood phylogenetic trees.

**Figure S4**. Amino‐acid alignment of oat homeolog proteins with orthologous proteins from close relatives.

**Figure S5**. Multidimensional scaling (MDS) plot of distances.

**Figure S6**. PCA plot projecting the data on the first two PCs.

**Figure S7**. Heat map showing distances between replicated samples and developmental stages.

**Figure S8**. Heat map of expression profiles standardized to the mean.

**Table S1**. Accumulation of individual tocopherols and tocotrienols in the seeds of oats during seed development.

**Table S2**. Transcript normalized counts and Pearson correlation with tocol concentrations.

**Table S3**. Vitamin E orthologous sequences used for the comparisons.
