# Supplementary material for: Subgenome‐specific assembly of vitamin E biosynthesis genes and expression patterns during seed development provide insight into the evolution of oat genome
Source: Plant Biotechnol J. 2016 May 26;14(11):2147–57. doi: 10.1111/pbi.12571 (PMC5096403; doi:10.1111/pbi.12571)
Supplement: Supplementary file 2 — Figure S2. Nucleotide and amino‐acid alignment of oat homeologs. [file PBI-14-2147-s010.pdf]

A1

HGGT

Nucleotide alignment

```
                    1                                                          60
HGGT_1      (1)     -----------------------------------------------AACGACGGCCATG
HGGT_2      (1)     TTCACAATTTCTCCTGCGCCGGAGAGAGATCCCCCG-CGGCGGCGGCGACGACGGCCATG
HGGT_3      (1)     -----AATTTCTCCTCCGCCGGAGAGATCCACCGCGGCGGCGGCGAAGACGACGGCGATG
                    61                                                        120
HGGT_1      (14)    GAAGCCACGGCGGTGGGGGCGGCGGCGCAGCTCCTCACAGACAGGAGAGGGCCCAGATGT
HGGT_2      (60)    GAGGCCACGGCGGTGGGGGCGGCGGCGCAGCTGCTAACAGATAGGAGACGGCCCACATGT
HGGT_3      (56)    GAAGCCACGGCGGTGGGGGCGGCGGCGCAGCTGCTAACAGATAGGAGAGGGCCCAGATGT
                    121                                                       180
HGGT_1      (74)    AGGGCTCGACTTGGAACGGCAAGATTATCCTTCCCAGGTCGATTTTCAGCGGAAGCTTTT
HGGT_2      (120)   AGGGCTCGGCTAGGAACGGCAAGATTATCCTTCCCAGGTCGATTTTCAGCGGAAGCTTTT
HGGT_3      (116)   AGGGCTCGGCTGGGAACGGCAAGATTATCCTTCCCAGGTCGATTTTCAGTGGAAGCTTTT
                    181                                                       240
HGGT_1      (134)   GCAGCCCAATTTCAAAGAGCTACTACTTTCAGTCATAGATTCAGTCCCACTTCTCAAGCT
HGGT_2      (180)   GCAGCCCAATTTCAAAGAGCTACTACTTTCAGTCATAGATTCAGTGCCACTTCTCAAGCT
HGGT_3      (176)   GCAGCCCAATTTCAAAGAGCTACTACTTTCAGTCATAGATTCAGTGCCACTTCTCAAGCT
                    241                                                       300
HGGT_1      (194)   ACCAGCCCTAGAAGAAACACAAGGAGGCAGTGCAGAGATGATCATCCAGCCATCCAAGTT
HGGT_2      (240)   ACCAGCCATAGAAGAAACACAAGGAGGCAGTGCAGAGATGTTCATCCAGCCATCCAAGTT
HGGT_3      (236)   ACCAGCCATAGAAGAAACACAAGGAGGCAGTGCAGAGATGTTCATCCATCCATCCAAGTT
                    301                                                       360
HGGT_1      (254)   GGATGCGGCGAGGTTACTCGTGAACAGCATGGTTCCGAAGTCAATCGGTTTCAGGAAATC
HGGT_2      (300)   GGATGCGGCGAGGTTACTCACGATCAGCATGGTTCCGAAGCCAATCGGTTTGAGGAAATC
HGGT_3      (296)   GGATGCGGCGAGGTTACTCGCGATCAGCATGGTTCCGAAACCAATCGGTTTGAGGAAATC
                    361                                                       420
HGGT_1      (314)   AGTAAAGAAGTTTCGAAGAAACTGCGCGCTTTCTATGAGTTTTGCAGGCCACACACAATT
HGGT_2      (360)   CGTGAAGAAGTTTCGAAGAAATTGCGCGCTTTCTATGAGTTTTGCAGGCCACACACAATT
HGGT_3      (356)   CGTGAAGAAGTTTCGAAGAAATTGCGCGCTTTCTATGAGTTTTGCAGGCCACACACAATT
                    421                                                       480
HGGT_1      (374)   TATGGCACTATAATAGGCATAACTTCAGTGTCCCTCCTGCCAATGACGAGCATAGATGAT
HGGT_2      (420)   TACGGCACTATAATAGGCATAACTTCAGTGTCCCTCCTGCCAATGAAGAGCATAGATGAT
HGGT_3      (416)   TACGGCACTATAATAGGCATAACTTCAGTGTCCCTCCTGCCAATGAAGAGCATAGATGAT
                    481                                                       540
HGGT_1      (434)   TTTACAGTGACGGTACTACTTGGATATATTGAGGCTTTGGCCGCTGCTTTATGTATGAAC
HGGT_2      (480)   TTTACAGTGACGGTACTACTGGGATATATCGAGGCTTTGGCCGCTGCTTTATGTATGAAC
HGGT_3      (476)   TTTACAGTGACGGTACTACTGGGATATATCGAGGCTTTGGCTGCTGCTTTATGTATGAAC
                    541                                                       600
HGGT_1      (494)   ATTTATGTGGTAGGGCTGAACCAGGTGTTTGACATTCAGATTGATAAGGTCAATAAGCCA
HGGT_2      (540)   ATTTATGTGGTAGGGCTGAATCAGGTGTTTGACATTCAGATTGATAAGGTCAATAAGCCA
HGGT_3      (536)   ATTTATGTGGTAGGGCTGAATCAGGTGTTTGACATTCAGATTGATAAGGTCAATAAGCCA
                    601                                                       660
HGGT_1      (554)   GGCCTTCCATTGGCTGCCGGGGAATTCTCAGTAACAACTGCAGTATATGTAGTACTCACA
HGGT_2      (600)   GGCCTTCCATTGGCAGCCGGGGAATTCTCAGTAGCAACTGGAGTTTTTTTAGTACTCACA
HGGT_3      (596)   GGCCTTCCATTGGCAGCCGGGGAATTCTCAGTAGCAACTGGAGTTGTTTTAGTACTCACA
                    661                                                       720
HGGT_1      (614)   TTCCTGATCATGAGCTTTAGCATCGGAATTCATTCTGGATCAGCACCATTGATGTGTGCT
HGGT_2      (660)   TTCCTGATCATGAGCTTCAGCATCGGGATTCATTCTGGATCAGCACCATTGATGTGTGCT
HGGT_3      (656)   TTCTTGATCATGAGCTTCAGCATCGGAATTCATTCTGGATCAGCACCATTGATGTGTGCT
                    721                                                       780
HGGT_1      (674)   TTAATTCTCAGCTTCCTTCTTGGAAGTGCATACTCCATTGAGGCTCCGTTACTCCGATGG
HGGT_2      (720)   TTACTTCTCAGCTTCCTTCTTGGAAGTGCATACTCCATTGAGGCTCCGTTACTCCGATGG
HGGT_3      (716)   TTACTTCTCAGCTTCCTTCTTGGAAGTGCATACTCCATTGAGGCTCCGTTACTCCGATGG
                    781                                                       840
HGGT_1      (734)   AAACGGCACGCTCTCCTCGCTGCATCCTGCATCCTGTTTGTGAGGGCTATCTTGGTTCAG
HGGT_2      (780)   AAACGGCACGCGCTCCTTGCTGCATCCTGCATCCTGTTTGTGAGGGCTATCTTGGTTCAG
HGGT_3      (776)   AAACGGCACGCGCTCCTCGCTGCATCCTGCATCCTGTTTGTGAGGGCTATCTTGGTTCAG
                    841                                                       900
HGGT_1      (794)   CTGGCTTTCTTTGCACACATGCAGCATCATATTCTGAAGAGGCCCTTGGCACCAACAAAA
HGGT_2      (840)   CTGGCTTTCTTTGCACACATGCAGCATCATGTTCTGAAGAGGCCCTTGGCACCAACAAAA
HGGT_3      (836)   CTGGCTTTCTTTGCACACATGCAGCATCATGTTCTGAAGAGGCCCTTGACACCAACAAAA
                    901                                                       960
HGGT_1      (854)   TCACTGATCTTTGCAACATTATTCATGTGCTGCTTCTCTGTGGTCATAGCTCTATTCAAG
HGGT_2      (900)   TCACTGGTCTTTGCAACATTATTCATGTGCTGCTTCTCTGTGGTCATAGCTCTATTCAAG
HGGT_3      (896)   TCATTGGTCTTTGCAACATTATTCATGTGCTGCTTCTCTGTGGTCATAGCTCTATTCAAG
```

```
                   961                                                       1020
HGGT_1    (914)    GATATTCCTGATATTGATGGAGACCGGGATTTTGGCATCCAATCCTTGAGTGTGAAATTG
HGGT_2    (960)    GATATTCCTGATATTGATGGAGACCGGGATTTTGGCATTCAATCCTTGAGTGTGAGATTG
HGGT_3    (956)    GATATTCCTGATATTGATGGAGACCGGGATTTTGGCATTCAATCCTTGAGTGTGAGATTG
                   1021                                                      1080
HGGT_1    (974)    GGCCCTCAAAGAGTGTATCAACTCTGTATAAGCATATTGTTGACAGCCTACGGGGCTGCC
HGGT_2   (1020)    GGACCTCAAAGAGTGTATCAACTCTGTATAAGCATATTGCTGACAGCCTATGGGGCTGCC
HGGT_3   (1016)    GGACCTCAAAGAGTGTATCAACTCTGTATAAGCATATTGCTGACAGCCTATGGGGCTGCC
                   1081                                                      1140
HGGT_1   (1034)    ACTCTTGTAGGAGCTTCATCCACAAACCTCTTTCAAAAGATCATCACTGTATCCGGCCAT
HGGT_2   (1080)    ACTCTGGTAGGAGCTTCATCCACAAACCTCTTTCAAAAGATCATCACTGTATCCGGCCAT
HGGT_3   (1076)    ACTCTGGTAGGAGCTTCATCCACAAACCTCTTTCAAAAGATCATCACTGTATCCGGCCAT
                   1141                                                      1200
HGGT_1   (1094)    GGCCTGCTTGCTTTCACACTTTGGCAGAGGGCACGGCACTTTGAGGTTGAAAACCAAGCG
HGGT_2   (1140)    GGCCTTCTTGCTTTCACACTTTGGCAGAGGGCACGACACTTTGAGGTTGAAAACCAAGCG
HGGT_3   (1136)    GGCCTGCTTGCTTTCACACTTTGGCAGAGGGCACGGCACTTTGAGGTTGAAAACCAAGCG
                   1201                                                      1260
HGGT_1   (1154)    CGCGTCACATCATTTTACATGTTCATTTGGAAGCTATTCTATGCAGAGTATTTCCTTATA
HGGT_2   (1200)    CGCGTCACATCATTTTACATGTTCATTTGGAAGCTATTCTATGCAGAGTATTTCCTTATA
HGGT_3   (1196)    CGCGTCACATCATTTTACATGTTCATTTGGAAGCTATTCTATGCAGAGTATTTCCTTATA
                   1261                                                      1320
HGGT_1   (1214)    CCATTTGTGCAATGAGAATGTACAAGGGGCAGCATTCGTGTGAACTATGTACAATTGTAA
HGGT_2   (1260)    CCATTTGTGCAGTGAGAATGTACAAGGGCCAGCATT--TGTGAACTATGTACACATGTAA
HGGT_3   (1256)    CCATTTGTGCAGTGAGAATGTACAAGGGCCAGCATT--TGTGAACTATGTACACATGTAA
                   1321                                                      1380
HGGT_1   (1274)    AGCAAATTAGGTGGAGGCCAATGCTTGGATTGTGTTTTTGTTTCATTGTGTAATTCGATA
HGGT_2   (1318)    AGCAAATTAGGTGGTG-CTAATGCTTGGACTGTGTTTTTATTTCATTATGTAATTCGATA
HGGT_3   (1314)    AGCAAATTAGGTGGTG-ATAATGCTTGGACTGTGTTTTTATTTCATTATGTAATTCGATA
                   1381                 1403
HGGT_1   (1334)    TTCTAGAGGAAGAGGCAAATGTT
HGGT_2   (1377)    TTCTAGAGGA-------------
HGGT_3   (1373)    TTCTA------------------
```

A2

Amino-acid alignment

```
                1                                                          60
HGGT_1    (1)   MEATAVGAAAQLLTDRRGPRCRARLGTARLSFPGRFSAEAFAAQFQRATTFSHRFSPTSQ
HGGT_2    (1)   MEATAVGAAAQLLTDRRRPTCRARLGTARLSFPGRFSAEAFAAQFQRATTFSHRFSATSQ
HGGT_3    (1)   MEATAVGAAAQLLTDRRGPRCRARLGTARLSFPGRFSVEAFAAQFQRATTFSHRFSATSQ
                61                                                        120
HGGT_1   (61)   ATSPRRNTRRQCRDDHPAIQVGCGEVTREQHGSEVNRFQEISKEVSKKLRAFYEFCRPHT
HGGT_2   (61)   ATSHRRNTRRQCRDVHPAIQVGCGEVTHDQHGSEANRFEEIREEVSKKLRAFYEFCRPHT
HGGT_3   (61)   ATSHRRNTRRQCRDVHPSIQVGCGEVTRDQHGSETNRFEEIREEVSKKLRAFYEFCRPHT
                121                                                       180
HGGT_1  (121)   IYGTIIGITSVSLLPMTSIDDFTVTVLLGYIEALAAALCMNIYVVGLNQVFDIQIDKVNK
HGGT_2  (121)   IYGTIIGITSVSLLPMKSIDDFTVTVLLGYIEALAAALCMNIYVVGLNQVFDIQIDKVNK
HGGT_3  (121)   IYGTIIGITSVSLLPMKSIDDFTVTVLLGYIEALAAALCMNIYVVGLNQVFDIQIDKVNK
                181                                                       240
HGGT_1  (181)   PGLPLAAGEFSVTTAVYVVLTFLIMSFSIGIHSGSAPLMCALILSFLLGSAYSIEAPLLR
HGGT_2  (181)   PGLPLAAGEFSVATGVFLVLTFLIMSFSIGIHSGSAPLMCALLLSFLLGSAYSIEAPLLR
HGGT_3  (181)   PGLPLAAGEFSVATGVVLVLTFLIMSFSIGIHSGSAPLMCALLLSFLLGSAYSIEAPLLR
                241                                                       300
HGGT_1  (241)   WKRHALLAASCILFVRAILVQLAFFAHMQHHILKRPLAPTKSLIFATLFMCCFSVVIALF
HGGT_2  (241)   WKRHALLAASCILFVRAILVQLAFFAHMQHHVLKRPLAPTKSLVFATLFMCCFSVVIALF
HGGT_3  (241)   WKRHALLAASCILFVRAILVQLAFFAHMQHHVLKRPLTPTKSLVFATLFMCCFSVVIALF
                301                                                       360
HGGT_1  (301)   KDIPDIDGDRDFGIQSLSVKLGPQRVYQLCISILLTAYGAATLVGASSTNLFQKIITVSG
HGGT_2  (301)   KDIPDIDGDRDFGIQSLSVRLGPQRVYQLCISILLTAYGAATLVGASSTNLFQKIITVSG
HGGT_3  (301)   KDIPDIDGDRDFGIQSLSVRLGPQRVYQLCISILLTAYGAATLVGASSTNLFQKIITVSG
                361                                         405
HGGT_1  (361)   HGLLAFTLWQRARHFEVENQARVTSFYMFIWKLFYAEYFLIPFVQ■
HGGT_2  (361)   HGLLAFTLWQRARHFEVENQARVTSFYMFIWKLFYAEYFLIPFVQ■
HGGT_3  (361)   HGLLAFTLWQRARHFEVENQARVTSFYMFIWKLFYAEYFLIPFVQ■
```

M Start codon   ■ Stop codon   X Identical   X Conservative   X Similar   X Weakly similar   X Non similar

B1

GGR

Nucleotide alignment

```
                  1                                                          60
GGR_1      (1) -AACTGCAAGCCGTGCGGCGGGGCCATCCCGCTGTGCATGGTGTCGGAGTTCGACCTGCC
GGR_2      (1) CAACTGCAAGCCGTGCGGCGGCGCCATCCCGCTCTGCATGGTGTCGGAGTTCGACCTGCC
GGR_3      (1) ----------------------------------TGCATGGTGTCGGAGTTCGACCTGCC
GGR_4      (1) ------------------------------------------------------------
               61                                                         120
GGR_1     (60) GCTCGACCTCGTGGACCGGAGGGTCACCAAGATGAAGATGATCTCGCCCTCCAACGTCGC
GGR_2     (61) GCTCGACCTCGTCGACAGGAGGGTCACCAAGATGAAGATGATCTCGCCCTCCAACGTCGC
GGR_3     (27) GCTCGACCTCGTCGACAGGAGGGTCACCAAGATGAAGATGATCTCGCCCTCCAACGTCGC
GGR_4      (1) ------------------------------------------------------------
               121                                                        180
GGR_1    (120) CGTCGACATCGGCCGCACGCTCGCCCCCCACGAGTACATCGGATGGTCAGGCGCGAGGT
GGR_2    (121) CGTCGACATCGGCCGCACGCTCGCCCCCCACGAGTACATCGGATGGTCAGGCGCGAGGT
GGR_3     (87) CGTCGACATCGGCCGCACGCTCGCCCCCCACGAGTACATCGGATGGTCAGGCGCGAGGT
GGR_4      (1) ------------------------------------------------------------
               181                                                        240
GGR_1    (180) GCTCGACGACTACCTCCGTAACCGGGCCCAGAAGGCCGGCGCCGAGGTCCTCAATGGCCT
GGR_2    (181) GCTCGACGACTACCTCCGGAACCGGGCACAGAAGGCCGGCGCCGAGGTCCTCAATGGTCT
GGR_3    (147) GCTCGACGACTACCTCCGGAACCGGGCACAGAAGGCCGGCGCCGAGGTCCTCAATGGTCT
GGR_4      (1) ------------------------------------------------------------
               241                                                        300
GGR_1    (240) CTTCCTAAGGTACGAGGAGCCCAAGGAGCGCAACGGCACGTACACGGTGCACTACAACCA
GGR_2    (241) CTTCCTAAGGTATGAGGAGCCCAAGGAGCGCAACGGCACGTACACTGTGCACTACAACCA
GGR_3    (207) CTTCCTAAGGTATGAGGAGCCCAAGGAGCGCAACGGCACGTACACTGTGCACTACAACCA
GGR_4      (1) ------------------------------------------------------------
               301                                                        360
GGR_1    (300) CTACGACAGCTCCAATGGCAAGGTGGGCGGCGAGAAGCGTTCGTTCGAGGTGGACGCGAT
GGR_2    (301) CTACGACAGCTCCAACGGCAAGGTGGGCGGCGAGAAGCGTTCGTTCGAGGTGGACGCGAT
GGR_3    (267) CTACGACAGCTCCAACGGCAAGGTGGGCGGCGAGAAGCGTTCGTTCGAGGTGGACGCGAT
GGR_4      (1) ------------------------------------------------------------
               361                                                        420
GGR_1    (360) CGTGGGCGCGGACGGCGCCAACTCCCGCGTGGCCAAGGACATGGGTGCCGGCGACTACGA
GGR_2    (361) CGTGGGCGCGGACGGCGCCAACTCCCGCGTGGCCAAGGACATGGGCGCCGGCGACTACGA
GGR_3    (327) CGTGGGCGCGGACGGCGCCAACTCCCGCGTGGCCAAGGACATGGGCGCCGGCGACTACGA
GGR_4      (1) ------------------------------------------------------------
               421                                                        480
GGR_1    (420) GTACGCCATCGCCTTTCAGGAGCGCGTGAAGATTCCCGACGACAAGATGCGGTACTACGA
GGR_2    (421) GTACGCCATCGCCTTCCAGGAGCGCGTGAAGATCCCCGATGACAAGATGCGCTACTACGA
GGR_3    (387) GTACGCCATCGCCTTCCAGGAGCGCGTGAAGATCCCCGATGACAAGATGCGCTACTACGA
GGR_4      (1) ------------------------------------------------------------
               481                                                        540
GGR_1    (480) GGAGCGCGCCGAGATGTACGTCGGCGACGACGTCTCCCCCGACTTCTACGGCTGGGTCTT
GGR_2    (481) GGAGCGTGCCGAGATGTACGTCGGCGACGACGTCTCCCCCGACTTCTACGGCTGGGTGTT
GGR_3    (447) GGAGCGCGCCGAGATGTACGTCGGCGACGACGTCTCCCCCGACTTCTACGGCTGGGTCTT
GGR_4      (1) -------------------------------------------------------GTCTT
               541                                                        600
GGR_1    (540) CCCCAAGTGCGACCACGTCGCCGTCGGCACCGGCACCGTCACCCACAAGGCCGACATCAA
GGR_2    (541) CCCCAAGTGCGACCACGTCGCCGTCGGCACCGGCACCGTCACCCACAAGGCCGACATCAA
GGR_3    (507) CCCCAAGTGCGACCACGTCGCCGTCGGCACCGGCACCGTCACCCACAAGGCCGACATCAA
GGR_4      (6) CCCCAAGTGCGACCACGTCGCGGTCGGCACCGGCACCGTCACGCACAAGGCCGACATCAA
               601                                                        660
GGR_1    (600) GAAGTTCCAGGCCGCCACCCGCCTCCGCGCGAAGGACAAGATCGAGGGCGGCAAGATCAT
GGR_2    (601) GAAGTTCCAGGCCGCCACTCGCCTCCGCGCCAAGGACAAGATCGAGGGCGGCAAGATCAT
GGR_3    (567) GAAGTTCCAGGCCG----------------------------------------------
GGR_4     (66) GAAGTTCCAGGCCGCCACTCGCCTCCGCGCCAAGGACAAGATCGAGGGCGGCAAGATCAT
               661                                                        720
GGR_1    (660) CCGCGTCGAGGCCCACCCCATCCCCGAACACCCACGCCCCAAAAGGGTGGCAGGGCGGGT
GGR_2    (661) CCGCGTCGAGGCTCACCCCATCCCCGAGCACCCACGTCCCAAAAGGGTGTCAGGGCGGGT
GGR_3    (581) ------------------------------------------------------------
GGR_4    (126) CCGCGTCGAGGCTCACCCCATCCCCGAGCACCCACGTCCCAAAAGGGTGTCAGGGCGGGT
               721                                                        780
GGR_1    (720) GACGCTGGTTGGCGACGCCGCGGGGTACGTGACCAAATGCTCCGGCGAGGGTATCTACTT
GGR_2    (721) GACGCTGGTCGGCGACGCCGCGGGGTACGTGACCAAGTGCTCCGGCGAGGGGATCTACTT
GGR_3    (581) ------------------------------------------------------------
GGR_4    (186) GACGCTGGTCGGCGACGCCGCGGGGTACGTGACCAAGTGCTCCGGCGAGGGGATCTACTT
```

```
                781                                                        840
GGR_1   (780)  TGCGGCGAAGAGCGGGCGGATGTGCGCGGAGGCGATCGTGGCCGGGTCGGCCAACGGGAC
GGR_2   (781)  CGCGGCGAAGAGCGGGCGGATGTGCGCGGAGGCGATCGTGGCCGGGTCGGCGAACGGGAC
GGR_3   (581)  ------------------------------------------------------------
GGR_4   (246)  CGCGGCGAAGAGCGGGCGGATGTGCGCGGAGGCGATCGTGGCCGGGTCGGCGAACGGGAC
                841                                                        900
GGR_1   (840)  GCGGCTGGTGGATGAGAGCGACCTGCGCAAGTACCTGGCGGAGTTCGACCGGCTCTACTG
GGR_2   (841)  GCGGCTGGTGGACGAGAGCGACCTGCGCAAGTACCTGGCGGAGTTTGACCGGCTCTACTG
GGR_3   (581)  ------------------------------------------------------------
GGR_4   (306)  GCGGCTGGTGGACGAGAGCGACCTGCGCAAGTACCTGGCGGAGTTCGACCGGCTCTACTG
                901                                                        960
GGR_1   (900)  GCCAACCTACAAGGTGCTGGACATCCTGCAGAAGGTGTTCTACCGCTCCAACGCGGCGCG
GGR_2   (901)  GCCCACGTACAAGGTGCTGGACATCCTGCAGAAGGTGTTCTACCGCTCCAACGCGGCGCG
GGR_3   (581)  ------------------------------------------------------------
GGR_4   (366)  GCCCACGTACAAGGTGCTGGACATCCTGCAGAAGGTGTTCTACCGCTCCAACGCGGCGCG
                961                                                       1020
GGR_1   (960)  GGAGGCGTTCGTGGAGATGTGCGCCGACGACTACGTGCAGCGGATGACCTTCGACAGCTA
GGR_2   (961)  GGAGGCGTTCGTGGAGATGTGCGCCGACGACTACGTGCAGCGGATGACCTTCGACAGCTA
GGR_3   (581)  ------------------------------------------------------------
GGR_4   (426)  GGAGGCGTTCGTGGAGATGTGCGCCGACGACTACGTGCAGCGCATGACCTTCGACAGCTA
                1021                                                      1080
GGR_1  (1020)  CCTCTACAAGCGCGTCGTGCCGGGCAACCCGATCGAGGACATCAAGCTCGCCGTCAACAC
GGR_2  (1021)  CCTCTACAAGCGCGTCGTGCCGGGCAACCCGATCGAGGACATCAAGCTCGCCGTCAACAC
GGR_3   (581)  ------------------------------------------------------------
GGR_4   (486)  CCTCTACAAGCGCGTCGTGCCGGGGAACCCGATCGAGGACATCAAGCTCGCCGTCAACAC
                1081                                                      1140
GGR_1  (1080)  CATCGGCAGCCTCGTCAGGGCCACCGCGCTGCGCCGGGAGATGAAGAACCTCACCTTGTG
GGR_2  (1081)  CATCGGCAGCCTCGTCAGGGCCACCGCGCTGCGCGGGGAGATGAAGAAGCTCACCTTGTG
GGR_3   (581)  ------------------------------------------------------------
GGR_4   (546)  CATCGGCAGCCTCGTCAGGGCCACCGCGCTGCGCGGGGAGATGAAGAAGCTCACCTTGTG
                1141                                                      1200
GGR_1  (1140)  ATCATCGAGATGATTTTCGCACGAACCTGACATCTGCACGCTTGGATTGTTACTACGAAC
GGR_2  (1141)  ATCATCGAGATGATTTTCCCAGGAACCTGACATCTGCACGCTCGGGTTGCTACTTCCAAC
GGR_3   (581)  ------------------------------------------------------------
GGR_4   (606)  ATCATCGAGATGATT---------------------------------------------
                1201                                                      1260
GGR_1  (1200)  TGCGGTGGATCGATCGGTATAGTAGAGATTCCCCGGCTGATCGAACAGACTTGGGA-CGG
GGR_2  (1201)  TGCGGTGGATTGATCGGTATAGTAGAGATTCCCCGGCTGATCGAGCAGATTTGGGATCGG
GGR_3   (581)  ------------------------------------------------------------
GGR_4   (621)  ------------------------------------------------------------
                1261                                                      1320
GGR_1  (1259)  ACGAGAAGACAATAAGGTTACGTGGATCGAATCGGAAGGTCGTGCCTCGATCGATCCTGT
GGR_2  (1261)  ACGAGAAGAGAATAAGGTTACGTGGATCGA-TCGGAAGGTCGTGCCTCGACCGATCGTGT
GGR_3   (581)  ------------------------------------------------------------
GGR_4   (621)  ------------------------------------------------------------
                1321                                                      1380
GGR_1  (1319)  ACGATTTTCTTGTACAACA-TACGCACGGTGATGTTCAGAGTCTTCAGACTTCAGAGCAG
GGR_2  (1320)  ACGATTTTCTTGTACAACAATACGCACGGTGATGTTCAGAAT---------TCAGAGCAG
GGR_3   (581)  ------------------------------------------------------------
GGR_4   (621)  ------------------------------------------------------------
                1381                                                      1440
GGR_1  (1378)  TTC---------------------------------------------------------
GGR_2  (1371)  TTCGTCTGGGTTATTGAAGCAAACCGATATTCCATATGTGACTGAATGTAGTATAAACTA
GGR_3   (581)  ------------------------------------------------------------
GGR_4   (621)  ------------------------------------------------------------
                1441           1459
GGR_1  (1381)  -------------------
GGR_2  (1431)  AGATAAATCGCGTGATAAA
GGR_3   (581)  -------------------
GGR_4   (621)  -------------------
```

B2

Amino-acid alignment

```
                   1                                                          60
GGR_1      (1)   NCKPCGGAIPLCMVSEFDLPLDLVDRRVTKMKMISPSNVAVDIGRTLAPHEYIGMVRREV
GGR_2      (1)   NCKPCGGAIPLCMVSEFDLPLDLVDRRVTKMKMISPSNVAVDIGRTLAPHEYIGMVRREV
GGR_3      (1)   -----------CMVSEFDLPLDLVDRRVTKMKMISPSNVAVDIGRTLAPHEYIGMVRREV
GGR_4      (1)   ------------------------------------------------------------
                 61                                                        120
GGR_1     (61)   LDDYLRNRAQKAGAEVLNGLFLRYEEPKERNGTYTVHYNHYDSSNGKVGGEKRSFEVDAI
GGR_2     (61)   LDDYLRNRAQKAGAEVLNGLFLRYEEPKERNGTYTVHYNHYDSSNGKVGGEKRSFEVDAI
GGR_3     (50)   LDDYLRNRAQKAGAEVLNGLFLRYEEPKERNGTYTVHYNHYDSSNGKVGGEKRSFEVDAI
GGR_4      (1)   ------------------------------------------------------------
                 121                                                       180
GGR_1    (121)   VGADGANSRVAKDMGAGDYEYAIAFQERVKIPDDKMRYYEERAEMYVGDDVSPDFYGWVF
GGR_2    (121)   VGADGANSRVAKDMGAGDYEYAIAFQERVKIPDDKMRYYEERAEMYVGDDVSPDFYGWVF
GGR_3    (110)   VGADGANSRVAKDMGAGDYEYAIAFQERVKIPDDKMRYYEERAEMYVGDDVSPDFYGWVF
GGR_4      (1)   ----------------------------------------------------------VF
                 181                                                       240
GGR_1    (181)   PKCDHVAVGTGTVTHKADIKKFQAATRLRAKDKIEGGKIIRVEAHPIPEHPRPKRVAGRV
GGR_2    (181)   PKCDHVAVGTGTVTHKADIKKFQAATRLRAKDKIEGGKIIRVEAHPIPEHPRPKRVSGRV
GGR_3    (170)   PKCDHVAVGTGTVTHKADIKKFQA------------------------------------
GGR_4      (3)   PKCDHVAVGTGTVTHKADIKKFQAATRLRAKDKIEGGKIIRVEAHPIPEHPRPKRVSGRV
                 241                                                       300
GGR_1    (241)   TLVGDAAGYVTKCSGEGIYFAAKSGRMCAEAIVAGSANGTRLVDESDLRKYLAEFDRLYW
GGR_2    (241)   TLVGDAAGYVTKCSGEGIYFAAKSGRMCAEAIVAGSANGTRLVDESDLRKYLAEFDRLYW
GGR_3    (194)   ------------------------------------------------------------
GGR_4     (63)   TLVGDAAGYVTKCSGEGIYFAAKSGRMCAEAIVAGSANGTRLVDESDLRKYLAEFDRLYW
                 301                                                       360
GGR_1    (301)   PTYKVLDILQKVFYRSNAAREAFVEMCADDYVQRMTFDSYLYKRVVPGNPIEDIKLAVNT
GGR_2    (301)   PTYKVLDILQKVFYRSNAAREAFVEMCADDYVQRMTFDSYLYKRVVPGNPIEDIKLAVNT
GGR_3    (194)   ------------------------------------------------------------
GGR_4    (123)   PTYKVLDILQKVFYRSNAAREAFVEMCADDYVQRMTFDSYLYKRVVPGNPIEDIKLAVNT
                 361               380
GGR_1    (361)   IGSLVRATALRREMKNLTL■
GGR_2    (361)   IGSLVRATALRGEMKKLTL■
GGR_3    (194)   -------------------■
GGR_4    (183)   IGSLVRATALRGEMKKLTL■
```

M Start codon ■ Stop codon X Identical X Conservative X Similar X Weakly similar X Non similar

C1

HPPD

Nucleotide alignment

```
                1                                                          60
HPPD_1    (1)   -----------------------------GTGCCCTTCCTGCCGGGGTTCGAGCGCGTGA
HPPD_2    (1)   -------------------CGAGACAGACCTGCCATTCCTGCCGGGGTTCGAGCGCGTGA
HPPD_3    (1)   GCTTCGTCAGCTACCCGGACGGGACAGACCTGCCATTCCTGCCGGGGTTCGAGCGCGTGA
                61                                                        120
HPPD_1   (32)   GCAGCCCCGGCGCCGTGGACTACGGGCTCACGCGGTTCGACCACGTCGTCGGCAACGTCC
HPPD_2   (42)   GCAGCCCGGGCGCCGTGGACTACGGCCTCACGCGGTTCGACCACGTCGTCGGCAACGTCC
HPPD_3   (61)   GCAGCCCGGGCGCCGTGGACTACGGTCTCACGCGGTTCGACCACGTCGTGGGCAACGTCC
                121                                                       180
HPPD_1   (92)   CGGAGATGGCCCCGGTCATCGCCTACATGAAAGGCTTCCTGGGGTTCCACGAGTTCGCCG
HPPD_2  (102)   CGGAGATGGCCCCGGTCATAGACTACATGAAAGGATTCTTGGGGTTCCACGAGTTCGCCG
HPPD_3  (121)   CGGAGATGGCCCCGGTCATCGCCTACATGAAAGGCTTCTTGGGGTTCCACGAGTTCGCCG
                181                                                       240
HPPD_1  (152)   AGTTCACCGCCGAGGACGTCGGCACGACCGAGAGCGGGCTCAACTCGGTGGTGCTCGCCA
HPPD_2  (162)   AGTTCACCGCCGAGGACGTGGGCACGACCGAGAGCGGGCTCAACTCGGTGGTGCTCGCCA
HPPD_3  (181)   AGTTCACCGCCGAGGACGTCGGCACGACCGAGAGCGGGCTCAACTCGGTGGTGCTCGCCA
                241                                                       300
HPPD_1  (212)   ACAACTCCGAGGCCGTGCTGCTGCCGCTCAACGAGCCCGTGCAGGGCACCAAGCGACGGA
HPPD_2  (222)   ACAACTCCGAGGCCGTGCTGCTGCCGCTCAACGAGCCCGTGCACGGCACGAAGCGCCGGA
HPPD_3  (241)   ACAACTCCGAGGCCGTGCTGCTGCCGCTCAACGAGCCCGTGCACGGCACCAAGCGCCGGA
                301                                                       360
HPPD_1  (272)   GCCAGATACAGACGTACCTGGAGTACCATGGAGGGCCCGGCGTGCAGCACATCGCGCTCG
HPPD_2  (282)   GCCAGATACAGACGTACCTGGAGTACCACGGCGGGCCCGGCGTGCAGCACATCGCGCTCG
HPPD_3  (301)   GCCAGATACAGACGTACCTGGAGTACCACGGCGGGCCCGGCGTGCAGCACATCGCGCTCG
                361                                                       420
HPPD_1  (332)   CCAGCAACGACGTGCTCAGGACGCTCAGGGAGATGCGGGCGCGCACGCCCATGGGTGGCT
HPPD_2  (342)   CCAGCAACGACGTGCTCAGGACGCTCAGGGAGATGCGGGCGCGCACGCCCATGGGCGGCT
HPPD_3  (361)   CCAGCAACGACGTGCTCAGGACGCTCAGGGAGATGAGGGCGCGCACGCCCATGGGCGGCT
                421                                                       480
HPPD_1  (392)   TCGAGTTCATGGCGCCACCCCAGGCGAAATACTATGACGGCGTGCGGCGCATCGCAGGTG
HPPD_2  (402)   TCGAGTTCATGGCGCCACCGCAGGCGAAGTACTATGACGGCGTGCGGCGCATCGCAGGTG
HPPD_3  (421)   TCGAGTTCATGGCGCCACCGCAGGCGAAGTACTATGACGGCGTGCGGCGCATCGCAGGTG
                481                                                       540
HPPD_1  (452)   ACGTGCTCTCCGAAGAGCAGATCAAGGAATGCCAGGAGCTGGGGGTGCTCGTCGACAGGG
HPPD_2  (462)   ACGTGCTCTCGGAAGAGCAGATCAAGGAATGCCAGGAGCTGGGGGTGCTCGTCGACAGGG
HPPD_3  (481)   ACGTCCTCTCGGAAGAGCAGATCAAGGAATGCCAGGAGCTGGGGGTGCTCGTCGACAGGG
                541                                                       600
HPPD_1  (512)   ATGACCAAGGGGTGTTGCTCCAAATCTTCACCAAGCCAGTAGGGGACAGGCCAACGTTTT
HPPD_2  (522)   ATGACCAAGGGGTGTTGCTCCAGATCTTCACCAAGCCAGTAGGGGACAGGCCAACGTTTT
HPPD_3  (541)   ATGACCAAGGGGTGTTGCTCCAGATCTTCACCAAGCCAGTAGGGGACAGGCCAACGTTTT
                601                                                       660
HPPD_1  (572)   TCCTGGAGATGATCCAAAGAATCGGGTGCATGGAGAAGGACGAGGTCGGGCAAGAGTACC
HPPD_2  (582)   TCCTGGAGATGATCCAAAGAATCGGGTGCATGGAGAAGGACGAGGTCGGGCAAGAGTACC
HPPD_3  (601)   TCCTGGAGATGATCCAAAGAATCGGGTGCATGGAGAAGGACGAGGTCGGGCAAGAGTACC
                661                                                       720
HPPD_1  (632)   AGAAGGGTGGCTGCGGCGGGTTTGGCAAGGGCAATTTCTCCGAGCTGTTCAAGTCCATTG
HPPD_2  (642)   AGAAGGGTGGCTGCGGCGGGTTCGGCAAGGGCAACTTCTCCGAGCTGTTCAAGTCCATTG
HPPD_3  (661)   AGAAGGGTGGCTGCGGCGGGTTCGGCAAGGGCAACTTCTCCGAGCTGTTCAAGTCCATTG
                721                                                       780
HPPD_1  (692)   AGGACTATGAGAAGTCCCTTGAGGCCAAGCAATCTGTTGTAGCTCAGCAATCCTAGGATA
HPPD_2  (702)   AGGACTATGAGAAGTCCCTTGAGGTCAAGCAATCTGTTGTAGCTCAGAAATCCTAGGATA
HPPD_3  (721)   AGGACTATGAGAAGTCCCTTGAGGTCAAGCAATCTGTTGTAGCTCAGAAATCCTAGGATA
                781                                                       840
HPPD_1  (752)   GAAGCTGGAGCAGCAGATCCAGTACTTTGTACTATCATGGAGCAAAAGAGAACACAGATG
HPPD_2  (762)   GAAGCTGGAGCAACAGATCCAGTACTTTGTA---TCGTGGAGCAAAAGAAAACACAGATG
HPPD_3  (781)   GAAGCTGGAGCAACAGATCCAGTACTTTGTA---TCGTGGAGCAAAAGAAAACACAGATG
                841                                                       900
HPPD_1  (812)   TTCTTTGCAAAATGCAACATCACAATTACATCCATGTTATAATGGTGAAGCTGAACACAT
HPPD_2  (819)   TTCTTTGTAATATGCAGCATCACAGTTACATCCATGCTATAATGGTGAAGCTGAACACAT
HPPD_3  (838)   TTCTTTGTAATATGCAGCATCACAGTTACATCCATGCTATAATGGTGAAGCTGAACACAT
                901                                                       960
HPPD_1  (872)   ATGTATCCTATGTACAATGAATGTAATAGATGGTTAGAGAGGGCTCACACAGATGAACAT
HPPD_2  (879)   ATGTATCATATGTACAATGAATGTAATAGATGGTTAGAGAAGGCTCACACAGATGAACAT
HPPD_3  (898)   ATGTATCATATGTACAATGAATGTAATAGATGGTTAGAGAGGGCTCACACAGATGAACAT
                961                                        1005
HPPD_1  (932)   ATGGCATTGTTGTACTAT---------------------------
HPPD_2  (939)   ATGGCATTGTTGTACTATCTTGTTGTACTATCTATCTATCTATCT
HPPD_3  (958)   ATGGCATTGTTGTACTCTCTGGTACTCTCTG--------------
```

C2

Amino-acid alignment

```
                   1                                                         60
HPPD_1      (1)    ---------VPFLPGFERVSSPGAVDYGLTRFDHVVGNVPEMAPVIAYMKGFLGFHEFAE
HPPD_2      (1)    ------ETDLPFLPGFERVSSPGAVDYGLTRFDHVVGNVPEMAPVIDYMKGFLGFHEFAE
HPPD_3      (1)    FVSYPDGTDLPFLPGFERVSSPGAVDYGLTRFDHVVGNVPEMAPVIAYMKGFLGFHEFAE
                   61                                                       120
HPPD_1     (52)    FTAEDVGTTESGLNSVVLANNSEAVLLPLNEPVQGTKRRSQIQTYLEYHGGPGVQHIALA
HPPD_2     (55)    FTAEDVGTTESGLNSVVLANNSEAVLLPLNEPVHGTKRRSQIQTYLEYHGGPGVQHIALA
HPPD_3     (61)    FTAEDVGTTESGLNSVVLANNSEAVLLPLNEPVHGTKRRSQIQTYLEYHGGPGVQHIALA
                   121                                                      180
HPPD_1    (112)    SNDVLRTLREMRARTPMGGFEFMAPPQAKYYDGVRRIAGDVLSEEQIKECQELGVLVDRD
HPPD_2    (115)    SNDVLRTLREMRARTPMGGFEFMAPPQAKYYDGVRRIAGDVLSEEQIKECQELGVLVDRD
HPPD_3    (121)    SNDVLRTLREMRARTPMGGFEFMAPPQAKYYDGVRRIAGDVLSEEQIKECQELGVLVDRD
                   181                                                      240
HPPD_1    (172)    DQGVLLQIFTKPVGDRPTFFLEMIQRIGCMEKDEVGQEYQKGGCGGFGKGNFSELFKSIE
HPPD_2    (175)    DQGVLLQIFTKPVGDRPTFFLEMIQRIGCMEKDEVGQEYQKGGCGGFGKGNFSELFKSIE
HPPD_3    (181)    DQGVLLQIFTKPVGDRPTFFLEMIQRIGCMEKDEVGQEYQKGGCGGFGKGNFSELFKSIE
                   241            257
HPPD_1    (232)    DYEKSLEAKQSVVAQQS■
HPPD_2    (235)    DYEKSLEVKQSVVAQKS■
HPPD_3    (241)    DYEKSLEVKQSVVAQKS■
```

M Start codon ■ Stop codon X Identical X Conservative X Similar X Weakly similar X Non similar

D1

VTE1

Nucleotide alignment

```
                   1                                                          60
VTE1_1      (1)   CTACGCTCCCACCCCGCGGGATCGGCCTCTGCGGACGCCGCACAGCGGGTATCACTACGA
VTE1_2      (1)   GTACGCTCCCACCCCGCGGGATCGGCCTCTGCGGACGCCGCACAGCGGGTATCACTACGA
VTE1_3      (1)   GTACGCTCCCACCCCGCGGGATCGGCCTCTGCGGACGCCGCACAGCGGGTATCACTACGA
                   61                                                        120
VTE1_1     (61)   CGGGACTGCCAGGCCTTTCTTCGAAGGATGGTACTTCAAGGTGTCCATTCCCGAGTGCAG
VTE1_2     (61)   CGGGACCGCCAGACCTTTCTTCGAAGGATGGTACTTCAAGGTGTCCATTCCCGAGTGCAG
VTE1_3     (61)   CGGGACCGCCAGACCTTTCTTCGAAGGATGGTACTTCAAGGTGTCCATTCCCGAGTGCAG
                   121                                                       180
VTE1_1    (121)   GCAGAGCTTCTGTTTCATGTACTCTGTTGAGAACCCATTTTTTCGTGATGGGATGAGTGC
VTE1_2    (121)   GCAGAGCTTCTGTTTCATGTACTCTGTTGAGAACCCATTTTTTCGTGATGGGATGAGTTC
VTE1_3    (121)   GCAGAGCTTCTGTTTCATGTACTCTGTTGAGAACCCATTTTTTCGTGATGGGATGAGTTC
                   181                                                       240
VTE1_1    (181)   TCTTGACCGGACCTTATACGGTCCACGGTTTACCGGGGCAGGAGCACAAATTCTTGGTGC
VTE1_2    (181)   TTTTGACCGGACCTTATACGGTCCACGGTTTACTGGGGCAGGAGCGCAAATTCTTGGTGC
VTE1_3    (181)   TTTTGACCGGACCTTATACGGTCCACGGTTTACTGGGGCAGGAGCGCAAATTCTTGGTGC
                   241                                                       300
VTE1_1    (241)   AGATGACAAGTACATATGCCAGTTCTCTGAAAAATCAAACAACTTTTGGGGGAGTAGAAA
VTE1_2    (241)   GGATGACAAGTACATATGCCAGTTCTCTGAAAAATCAAACAACTTTTGGGGGAGTAGACA
VTE1_3    (241)   GGATGACAAGTACATATGCCAGTTCTCTGAAAAATCAAACAACTTTTGGGGGAGTAGACA
                   301                                                       360
VTE1_1    (301)   TGAGCTAACTCTCGGAAACACTTTCATTTCCAATAAAGACTCAACTCCCCCGGAACGGGA
VTE1_2    (301)   TGAGCTAACTCTCGGCAACACTTTCATTCCCAATAAAGACTCAACTCCCCCGGAACGGGA
VTE1_3    (301)   TGAGCTAACTCTCGGCAACACTTTCATTCCCAATAAAGACTCAACTCCCCCCGAACGGGA
                   361                                                       420
VTE1_1    (361)   AGTCCCTCCTCAGGAATTTTCAAATCGTGTTTTGGAAGGCTACCAAGTCACACCAGTTTG
VTE1_2    (361)   AGTCCCTCCTCAGGAATTTTCAAATCGTGTTTTGGAAGGCTACCAAGTCACACCAGTTTG
VTE1_3    (361)   AGTCCCTCCTCAGGAATTTTCAAATCGTGTTTTGGAAGGCTACCAAGTCACACCAGTTTG
                   421                                                       480
VTE1_1    (421)   GCATCAGGGTTTCATACGTGATGATGGAAGGTCAAAGTATGTGCCGAATGTACAAACAGC
VTE1_2    (421)   GCATCAGGGTTTCATACGTGATGATGGAAGGTCAAAGTATGTGCCAAATGTCCAAACAGC
VTE1_3    (421)   GCATCAGGGTTTCATACGTGATGATGGAAGGTCAAAGTATGTGCCAAATGTCCAAACAGC
                   481                                                       540
VTE1_1    (481)   TCGTTGGGAGTACAGCACTCGTCCGGTATATGGGTGGGGTGATGTCACATCTAAGCAGAA
VTE1_2    (481)   TCGTTGGGAGTACAGCACTCGTCCTGTGTATGGGTGGGGTGATGTCACATCTAAGCAGAA
VTE1_3    (481)   TCGTTGGGAGTACAGCACTCGTCCTGTGTATGGGTGGGGTGATGTCACATCTAAGCAGAA
                   541                                                       600
VTE1_1    (541)   GTCGACAGCTGGTTGGCTTGCTGCTTTTCCATTCTTTGAACCTCATTGGCAAATATGCAT
VTE1_2    (541)   GTCGACAGCTGGTTGGCTTGCTGCTTTTCCATTCTTTGAACCTCATTGGCAAATATGCAT
VTE1_3    (541)   GTCGACAGCTGGTTGGCTTGCTGCTTTTCCATTCTTTGAACCTCATTGGCAAATATGCAT
                   601                                                       660
VTE1_1    (601)   GGCTGGTGGCCTATCCACAGGATGGATAGAATGGGATGGAGAACGGTTTGAATTTGAAAA
VTE1_2    (601)   GGCTGGTGGCCTATCCACAGGATGGATAGAATGGGATGGAGAACGGTTTGAATTCGAAAA
VTE1_3    (601)   GGCTGGTGGCCTATCCACAGGATGGATAGAATGGGATGGAGAACGGTTTGAATTCGAAAA
                   661                                                       720
VTE1_1    (661)   TGCTCCCTCTTACTCAGAAAAGAACTGGGGCGGGGGTTTCCCAAGAAAGTGGTACTGGAT
VTE1_2    (661)   TGCTCCCTCTTACTCAGAAAAGAACTGGGGCGGGGGTTTCCCAAGAAAGTGGTACTGGAT
VTE1_3    (661)   TTCTCCCTCTTACTCAGAAAAGAACTGGGGCGGGGGTTTCCCAAGAAAGTGGTACTGGAT
                   721                                                       780
VTE1_1    (721)   TCAGTGCAATGTCTTCTCAGGCACATCTGGTGAGGTTGCTCTAACTGCTGCCGGTGGATT
VTE1_2    (721)   TCAGTGCAATGTCTTCTCAGGCGCATCTGGAGAAGTTGCTTTAACTGCTGCCGGTGGGTT
VTE1_3    (721)   TCAGTGCAATGTCTTCTCAGGCGCATCTGGAGAAGTTGCTTTAACTGCTGCCGGTGGGTT
                   781                                                       840
VTE1_1    (781)   GAGGAAAATTGGATTGGGAGATACATACGAGAGTCCTTCCCTGATTGGCGTCCATCATGA
VTE1_2    (781)   GAGGAAAATTGGATTGGGAGATACATACGAGAGTCCTTCCCTGATTGGCGTCCATCATGA
VTE1_3    (781)   GAGGAAAATTGGATTGGGAGATACATACGAGAGTCCTTCCCTGATTGGCGTCCATCATGA
                   841                                                       900
VTE1_1    (841)   GGGAAATTTTTATGAGTTTGTGCCTTGGACTGGGACAGTAAGCTGGGACATTGCTCCTTG
VTE1_2    (841)   GGGAAAATTTTATGAGTTTGTGCCTTGGACTGGGACAGTAAGCTGGGACATTGCTCCTTG
VTE1_3    (841)   GGGAAAATTTTATGAGTTTGTGCCTTGGACTGGGACAGTAAGCTGGGACATTGCTCCTTG
                   901                                                       960
VTE1_1    (901)   GGGTCACTGGAGGATGTCTGGCGAGAACAAAAATCATCTGGTGGAAATAGAAGCAACCAC
VTE1_2    (901)   GTGTCACTGGAGGATGTCTGGCGAGAACAAAAATCATCTGGTGGAAATAGAAGCAACCAC
VTE1_3    (901)   GGGTCACTGGAGGATGTCTGGCGAGAACAAAAATCATCTGGTGGAAATAGAAGCAACCAC
```

```
                  961                                                          1020
VTE1_1    (961)  CAAAGAACTAGGCACTGCTTTGAGAGCTCCAACAGTGGAGGCTGGGTTGGTACCAGCATG
VTE1_2    (961)  CAAAGAACCCGGCACTGCTTTGAGAGCTCCAACAATGGAGGCTGGGTTGGTACCAGCATG
VTE1_3    (961)  CAAAGAACCCGGCACTGCTTTGAGAGCTCCAACAATGGAGGCTGGGTTGGTACCAGCATG
                  1021                                                         1080
VTE1_1   (1021)  CAAAGACACCTGTTATGGAGATCTCAAGCTGCAAATGTGGGAAAAAAGATTTGATGGGAG
VTE1_2   (1021)  CAAAGACACCTGTTATGGAGATCTCAAGCTGCAAATGTGGGAAAAAAGATTTGATGGGAG
VTE1_3   (1021)  CAAAGACACCTGTTATGGAGATCTCAAGCTGCAAATGTGGGAAAAAAGATTTGATGGGAG
                  1081                                                         1140
VTE1_1   (1081)  CAAGGGGAAGATGATACTTGAGGCCACAAGCAACATGGCGGCGGTGGAAGTTGGAGGAGG
VTE1_2   (1081)  CAAGGGGAAGATGATACTTGAGGCCACAAGCAACATGGCGGCGGTGGAAGTTGGAGGAGG
VTE1_3   (1081)  CAAGGGGAAGATGATACTTGAGGCCACAAGCAACATGGCGGCGGTGGAAGTTGGAGGAGG
                  1141                                                         1200
VTE1_1   (1141)  TCCCTGGTTCAACGGGTGGAAAGGCACGACTGTTTCAAATGAGCTTGTGAATAACCTTGT
VTE1_2   (1141)  TCCCTGGTTCAACGGGTGGAAAGGCACGACTGCTTCAAATGAGCTTGTGAATAATCTTGT
VTE1_3   (1141)  TCCCTGGTTCAACGGGTGGAAAGGCACGACTGCTTCAAATGAGCTTGTGAATAATCTTGT
                  1201                                                         1260
VTE1_1   (1201)  TGGTACTCAGATTGACGTGGAGAGCCTCTTCCCTATTCCAGTTCTCAAGCCCCCTGGCCT
VTE1_2   (1201)  TGGTACTCAGATTGACGTGGAGAGCCTCTTCCCTATTTCAGTTCTCAAGCCCCCTGGCCT
VTE1_3   (1201)  TGGTACTCAGATTGACGTGGAGAGCCTCTTCCCTATTTCAGTTCTCAAGCCCCCTGGCCT
                  1261                                                         1320
VTE1_1   (1261)  GTAGGTTTATTCAGCAGAACTATGAAGAAACTGAAATGACAAGGCACTCGAGGTTCTC--
VTE1_2   (1261)  GTAGGTTTATTCAGGATAACTATGGAGAAATTGAAATGACAAGGCACTCCAGGTTCTCTT
VTE1_3   (1261)  GTAGGTTTATTCAGGACAACTATGGAGAAATTGAAATGACAAGGCACTCCAGGTTCTCTT
                  1321
VTE1_1   (1319)  ----------
VTE1_2   (1321)  GGCTTAGCTT
VTE1_3   (1321)  GGCTTAGCTT
```

D2

Amino-acid alignment

```
                   1                                                          60
VTE1_1      (1)    YAPTPRDRPLRTPHSGYHYDGTARPFFEGWYFKVSIPECRQSFCFMYSVENPFFRDGMSA
VTE1_2      (1)    YAPTPRDRPLRTPHSGYHYDGTARPFFEGWYFKVSIPECRQSFCFMYSVENPFFRDGMSS
VTE1_3      (1)    YAPTPRDRPLRTPHSGYHYDGTARPFFEGWYFKVSIPECRQSFCFMYSVENPFFRDGMSS
                   61                                                        120
VTE1_1     (61)    LDRTLYGPRFTGAGAQILGADDKYICQFSEKSNNFWGSRNELTLGNTFISNKDSTPPERE
VTE1_2     (61)    FDRTLYGPRFTGAGAQILGADDKYICQFSEKSNNFWGSRHELTLGNTFIPNKDSTPPERE
VTE1_3     (61)    FDRTLYGPRFTGAGAQILGADDKYICQFSEKSNNFWGSRHELTLGNTFIPNKDSTPPERE
                   121                                                       180
VTE1_1    (121)    VPPQEFSNRVLEGYQVTPVWHQGFIRDDGRSKYVPNVQTARWEYSTRPVYGWGDVTSKQK
VTE1_2    (121)    VPPQEFSNRVLEGYQVTPVWHQGFIRDDGRSKYVPNVQTARWEYSTRPVYGWGDVTSKQK
VTE1_3    (121)    VPPQEFSNRVLEGYQVTPVWHQGFIRDDGRSKYVPNVQTARWEYSTRPVYGWGDVTSKQK
                   181                                                       240
VTE1_1    (181)    STAGWLAAFPFFEPHWQICMAGGLSTGWIEWDGERFEFENAPSYSEKNWGGGFPRKWYWI
VTE1_2    (181)    STAGWLAAFPFFEPHWQICMAGGLSTGWIEWDGERFEFENAPSYSEKNWGGGFPRKWYWI
VTE1_3    (181)    STAGWLAAFPFFEPHWQICMAGGLSTGWIEWDGERFEFENSPSYSEKNWGGGFPRKWYWI
                   241                                                       300
VTE1_1    (241)    QCNVFSGTSGEVALTAAGGLRKIGLGDTYESPSLIGVHHEGNFYEFVPWTGTVSWDIAPW
VTE1_2    (241)    QCNVFSGASGEVALTAAGGLRKIGLGDTYESPSLIGVHHEGKFYEFVPWTGTVSWDIAPW
VTE1_3    (241)    QCNVFSGASGEVALTAAGGLRKIGLGDTYESPSLIGVHHEGKFYEFVPWTGTVSWDIAPW
                   301                                                       360
VTE1_1    (301)    GHWRMSGENKNHLVEIEATTKELGTALRAPTVEAGLVPACKDTCYGDLKLQMWEKRFDGS
VTE1_2    (301)    CHWRMSGENKNHLVEIEATTKEPGTALRAPTMEAGLVPACKDTCYGDLKLQMWEKRFDGS
VTE1_3    (301)    GHWRMSGENKNHLVEIEATTKEPGTALRAPTMEAGLVPACKDTCYGDLKLQMWEKRFDGS
                   361                                                       420
VTE1_1    (361)    KGKMILEATSNMAAVEVGGGPWFNGWKGTTVSNELVNNLVGTQIDVESLFPIPVLKPPGL
VTE1_2    (361)    KGKMILEATSNMAAVEVGGGPWFNGWKGTTASNELVNNLVGTQIDVESLFPISVLKPPGL
VTE1_3    (361)    KGKMILEATSNMAAVEVGGGPWFNGWKGTTASNELVNNLVGTQIDVESLFPISVLKPPGL
                   421
VTE1_1    (421)    ■
VTE1_2    (421)    ■
VTE1_3    (421)    ■
```

M Start codon ■ Stop codon X Identical X Conservative X Similar X Weakly similar X Non similar

E1

VTE2

Nucleotide alignment

```
                    1                                                         60
VTE2_1        (1) CGGCAGATGGACTCGCTCTGCCTCCGCCCGTCCGCGCCCGCCTGCGCCGCCGCCGCCGCC
VTE2_2        (1) ------------------------------------------------------------
VTE2_3        (1) ------------------------------------------------------------
VTE2_4        (1) ------------------------------------------------------------
VTE2_5        (1) -----------------------------------------------------------C
                    61                                                       120
VTE2_1       (61) CGCCGGCGAGATCATATTCTACCATCATTCTGTTCTATCCAACGAAGTGGTAAAGGGCGA
VTE2_2        (1) ------------------------------------------------------------
VTE2_3        (1) ------------------------------------------------------------
VTE2_4        (1) ------------------------------------------------------------
VTE2_5        (2) TCTCATCGAGGGTTATATCTACCATCATT-TGTTCTATCCAGCGAAGTGGTAAAGG-CGA
                    121                                                      180
VTE2_1      (121) GTTTCTTTGTCCATCC-AAGGGTCCAAAGGCCCTACTGATGATCATTGTAAAAAATTCTT
VTE2_2        (1) -----TTTGTCCATCC-AAGGGTCCAAAGGCCCCACTGTTGATCATTGTAAAAAGTTCTT
VTE2_3        (1) ------------------------------CCCCACTGTTGATCATTGTAAAAAGTTCTT
VTE2_4        (1) ----------------------------GGCCATACTGTTGATCATTGTA-AAATTTCTT
VTE2_5       (60) GTTACTTTGTCCATCCTAAGTATCCAAAGGCCCCAGTGTATATCAT-GTAGAAAGTTCTT
                    181                                                      240
VTE2_1      (180) GGATTGGAAATACTCCTACCATAGGATATCACATCAATCAAGAAATACTTCTGCAAATGC
VTE2_2       (55) GGATTGGAAATACTCCTACCATAGGATATCACATCAATCAAGAAATACTTCTGCAAATGC
VTE2_3       (31) GGATTGGAAATACTCCTACCATAGGATATCACATCAATCAAGAAATACTTCTGCAAATGC
VTE2_4       (32) AGATTGAAAATACTCCTACCATTGGATATCACATCAATCAAGAAATACTTCTGCAAATGT
VTE2_5      (119) GGATTGGAAATACTCCTTCCATAGGATATCACATCAATCAAGAAATACTTCTGCAAATGC
                    241                                                      300
VTE2_1      (240) TGGGCAGTCACTACAGCCTGAAACTGAAGCGCACGATCCAGCAAGCATCTGGAAGCCAAT
VTE2_2      (115) TGGGCAATCACTACTACCTGAAACTGAAGCGCACGATCCAGCAAGCATCTGGAAGTCAAT
VTE2_3       (91) TGGGCAATCACTACTACCTGAAACTGAAGCGCACGATCCAGCAAGCATCTGGAAGTCAAT
VTE2_4       (92) TGGGCAATCACTACAGCCTGAAACTGAAGCACACCATCCAGCAAGCATCTGGAAGCCAAT
VTE2_5      (179) TGGGCAATCACTACTACCTGAAACTGAAGCGCACGATCCAGCAAGCATCTGGAAGTCAAT
                    301                                                      360
VTE2_1      (300) ATCTTCT---CTGGATGCATTTTACAGGTTTTCTCGGCCACATACCGTCATAGGAACAGC
VTE2_2      (175) ATCTTCTTCTCTGGATGCATTTTACAGGTTTTCGCGGCCACATACCGTCATAGGAACAGC
VTE2_3      (151) ATCTTCTTCTCTGGATGCATTTTACAGGTTTTCGCGGCCACATACCGTCATAGGAACAGC
VTE2_4      (152) ATCATCTTCTCTGGATGCATTTTACAGGTTTTCTCGGCCACATACCGTCATAGGAACAGC
VTE2_5      (239) ATCTTCTTCTCTGGATGCATTTTACAGGTTTTCGCGGCCACATACCGTCATAGGAACAGC
                    361                                                      420
VTE2_1      (357) ACTAAGCATAGTCTCAGTTTCCCTACTAGCTGTCGAGAGCTTGTCTGATATTTCGCCCCT
VTE2_2      (235) ACTAAGCATAGTCTCAGTTTCCCTACTAGCTGTCGAGAGCTTGTCTGATATTTCGCCCTT
VTE2_3      (211) ACTAAGCATAGTCTCAGTTTCCCTACTAGCTGTCGAGAGCTTGTCTGATATTTCGCCCTT
VTE2_4      (212) ACTAAGCATAGTCTCAGTTTCCCTACTAGCTGTCGAGAGCTTGTCTGATATTTCGCCCTT
VTE2_5      (299) ACTAAGCATAGTCTCAGTTTCCCTACTAGCTGTCGAGAGCTTGTCTGATATTTCGCCCTT
                    421                                                      480
VTE2_1      (417) GTTCCTCACTGGTTTGCTGGAGGCAGTGGTGGCTGCTTTTTTTATGAACATCTATATTGT
VTE2_2      (295) GTTCCTCACTGGTTTGCTGGAGGCAGTGGTGGCTGCTTTTTTCATGAACATCTATATTGT
VTE2_3      (271) GTTCCTCACTGGTTTGCTGGAGGCAGTGGTGGCTGCTTTTTTCATGAACATCTATATTGT
VTE2_4      (272) GTTCCTCACTGGTTTGCTGGAGGCAGTGGTGGCTGCTTTTTTTATGAACATCTATATTGT
VTE2_5      (359) GTTCCTCACTGGTTTGCTGGAGGCAGTGGTGGCTGCTTTTTTCATGAACATCTATATTGT
                    481                                                      540
VTE2_1      (477) TGGATTGAACCAGCTGTTTGACATTGAAATAGACAAGGTTAACAAGCCAACTCTTCCACT
VTE2_2      (355) TGGATTGAATCAGCTGTTTGACATTGAAATAGACAAGGTTAACAAGCCAACTCTTCCACT
VTE2_3      (331) TGGATTGAATCAGCTGTTTGACATTGAAATAGACAAGGTTAACAAGCCAACTCTTCCACT
VTE2_4      (332) TGGATTGAACCAGGTGTTTGACATTGAAATAGATAAGGTTAACAAGCCAACTCTTCCACT
VTE2_5      (419) TGGATTGAATCAGCTGTTTGACATTGAAATAGACAAGGTTAACAAGCCAACTCTTCCACT
                    541                                                      600
VTE2_1      (537) AGCATCTGGGGAATACTCTCCTGCAACTGGGGTTGCAATAGTGTCAGTTTCTGCTGCTAT
VTE2_2      (415) AGCATCTGGGGAATACTCTCCTGCAACTGGGGTTGCGATAGTGTCAGTTTCTGCTGCTAT
VTE2_3      (391) AGCATCTGGGGAATACTCTCCTGCAACTGGGGTTGCGATAGTGTCAGTTTCTGCTGCTAT
VTE2_4      (392) AGCATCTGGGGAATACTCTCCTGCAACTGGGGTTGCGATAGTGTCAGTTTCTGCTGCTAT
VTE2_5      (479) AGCATCTGGGGAATACTCTCCTGCAACTGGGGTTGCGATAGTGTCAGTTTCTGCTGCTAT
                    601                                                      660
VTE2_1      (597) GAGCTTTGCCCTTGGATGGCTTGTTGGATCACCACCTCTGTTTTGGGCTCTTTTTATTAG
VTE2_2      (475) GAGCTTTGCCCTTGGATGGCTTGTTGGATCACCACCTCTGTTTTGGGCTCTTTTTATTAG
VTE2_3      (451) GAGCTTTGCCCTTGGATGGCTTGTTGGATCACCACCTCTGTTTTGGGCTCTTTTTATTAG
VTE2_4      (452) GAGCTTTGCCCTTGGATGGCTTGTTGGATCACCACCTCTGTTTTGGGCTCTTTTTATTAG
VTE2_5      (539) GAGCTTTGCCCTTGGATGGCTTGTTGGATCACCACCTCTGTTTTGGGCTCTTTTTATTAG
```

```
                   661                                                        720
VTE2_1      (657)  CTTTGTTCTTGGAACTGCTTATTCAGTCAATCTCCCATACTTTCGGTGGAAGAGGTTTGC
VTE2_2      (535)  CTTTGTTCTTGGAACTGCTTATTCAGTCAATCTCCCATACTTTCGATGGAAGAGGTTTGC
VTE2_3      (511)  CTTTGTTCTTGGAACTGCTTATTCAGTCAATCTCCCATACTTTCGATGGAAGAGGTTTGC
VTE2_4      (512)  CTTTGTTCTTGGAACTGCTTATTCAGTCAATCTCCCATACTTCCGATGGAAGAGGTTTGC
VTE2_5      (599)  CTTTGTTCTTGGAACTGCTTATTCAGTCAATCTCCCATACTTTCGATGGAAGAGGTTTGC
                   721                                                        780
VTE2_1      (717)  TGTTGTTGCAGCACTCTGCATATTAGCAGTGCGGGCGGTGATTGTTCAACTGGCATTTTT
VTE2_2      (595)  TGTTGTTGCAGCACTCTGCATATTAGCAGTGCGTGCTGTGATTGTTCAACTGGCATTTTT
VTE2_3      (571)  TGTTGTTGCAGCACTCTGCATATTAGCAGTGCGTGCTGTGATTGTTCAACTGGCATTTTT
VTE2_4      (572)  TGTTGTTGCAGCACTCTGCATATTAGCAGTGCGTGCTGTGATTGTTCAACTGGCATTTTT
VTE2_5      (659)  TGTTGTTGCAGCACTCTGCATATTAGCAGTGCGTGCTGTGATTGTTCAACTGGCATTTTT
                   781                                                        840
VTE2_1      (777)  TCTCCACATTCAGACATTTGTTTTCAGAAGGCCGGCAGTCTTTTCAAAGCCATTGATATT
VTE2_2      (655)  TCTCCACATTCAGACATTTGTTTTCAGAAGGCCGGCAGTCTTTTCAAAGCCATTGATATT
VTE2_3      (631)  TCTCCACATTCAGACATTTGTTTTCAGAAGGCCGGCAGTCTTTTCAAAGCCATTGATATT
VTE2_4      (632)  TCTCCACATTCAGACATTTGTTTTCAGAAGGCCGGCAGTCTTTTCAAAGCCATTGATATT
VTE2_5      (719)  TCTCCACATTCAGACATTTGTTTTCAGAAGGCCGGCAGTCTTTTCAAAGCCATTGATATT
                   841                                                        900
VTE2_1      (837)  TGCAACTGCATTCATGACCTTCTTCTCAGTTGTAATAGCATTATTCAAGGATATCCCTGA
VTE2_2      (715)  TGCAACTGCATTCATGACCTTCTTCTCAGTTGTAATAGCGTTATTCAAGGATATCCCTGA
VTE2_3      (691)  TGCAACTGCATTCATGACCTTCTTCTCAGTTGTAATAGCGTTATTCAAGGATATCCCTGA
VTE2_4      (692)  TGCAACTGCATTCATGACCTTCTTCTCAGTTGTAATAGCATTATTCAAGGATATCCCTGA
VTE2_5      (779)  TGCAACTGCATTCATGACCTTCTTCTCAGTTGTAATAGCGTTATTCAAGGATATCCCTGA
                   901                                                        960
VTE2_1      (897)  TATTGAAGGGGACCGCATTTTTGGAATTCAATCTTTCAGTGTTAGATTAGGGCAAAACAA
VTE2_2      (775)  TATTGAAGGGGACCGCATTTTTGGAATTCAATCTTTCAGTGTTAGATTAGGGCAAAACAA
VTE2_3      (751)  TATTGAAGGGGACCGCATTTTTGGAATTCAATCTTTCAGTGTTAGATTAGGGCAAAACAA
VTE2_4      (752)  TATTGAAGGGGACCGCATTTTTGGAATTCAATCTTTCAGTGTTAGATTAGGACAAAACAA
VTE2_5      (839)  TATTGAAGGGGACCGCATTTTTGGAATTCAATCTTTCAGTGTTAGATTAGGGCAAAACAA
                   961                                                       1020
VTE2_1      (957)  GGTTTTCTGGATTTGTGTCGGCCTACTTGAGATGGCCTATGGTGTTGCAATACTGATGGG
VTE2_2      (835)  GGTTTTCTGGATTTGTGTCGGCCTACTTGAGATGGCCTATGGTGTTGCAATACTGATGGG
VTE2_3      (811)  GGTTTTCTGGATTTGTGTCGGCCTACTTGAGATGGCCTATGGTGTTGCAATACTGATGGG
VTE2_4      (812)  GGTTTTCTGGATCTGTGTTGGTCTACTTGAGATGGCCTATGGTGTTGCAATACTGATGGG
VTE2_5      (899)  GGTTTTCTGGATTTGTGTCGGCCTACTTGAGATGGCCTATGGTGTTGCAATACTGATGGG
                   1021                                                      1080
VTE2_1     (1017)  GGCAACTTCTTCCAGTTTGTGGAGCAAATCTGTAACCGTCGCAGGCCATGCCATCCTCGC
VTE2_2      (895)  GGCAACTTCTTCCAGTTTGTGGAGCAAATCTGTAACCGTCGCAGGCCATGCCATCCTCGC
VTE2_3      (871)  GGCAACTTCTTCCAGTTTGTGGAGCAAATCTGTAACCGTCGCAGGCCATGCCATCCTCGC
VTE2_4      (872)  GGCTACTTCTTCCAGTTTGTGGAGCAAATCTGTAACTGTCGCAGGCCATGCCATCCTCGC
VTE2_5      (959)  GGCAACTTCTTCCAGTTTGTGGAGCAAATCTGTAACCGTCGCAGGCCATGCCATCCTCGC
                   1081                                                      1140
VTE2_1     (1077)  CTCGGTCTTATGGAGCTGCGCACGGTCGGTCGACCTCACAAGCAAGGCTGCAATAACATC
VTE2_2      (955)  CTCGGTCTTATGGAGCTGCGCACGGTCGGTCGACCTCACAAGCAAGGCTGCAATAACATC
VTE2_3      (931)  CTCGATCTTGTGGAGCTGCGCACGGTCGGTCGACCTCACAAGCAAGGCTGCAATAACATC
VTE2_4      (932)  CTCGCTCTTATGGAGCTGCGCACGGTCGGTCGACCTCACAAGCAAGGCTGCAATAACATC
VTE2_5     (1019)  CTCGGTCTTATGGAGCTGCGCACGGTCGGTCGACCTCACAAGCAAGGCTGCAATAACATC
                   1141                                                      1200
VTE2_1     (1137)  CTTCTACATGTTCATCTGGAAGCTGTTCTACGCGGAGTACCTGCTCATTCCTCTTGTAAG
VTE2_2     (1015)  CTTCTACATGTTCATCTGGAAGCTGTTCTACGCGGAGTACCTGCTCATTCCTCTTGTAAG
VTE2_3      (991)  CTTCTACATGTTCATCTGGAAGCTGTTCTACGCGGAGTACCTGCTCATTCCCCTTGTAAG
VTE2_4      (992)  CTTCTACATGTTCATCTGGAAGCTGTTCTACGCGGAGTACCTGCTCATTCCTCTTGTAAG
VTE2_5     (1079)  CTTCTACATGTTCATCTGGAAGCTGTTCTACGCGGAGTACCTGCTCATTCCTCTTGTAAG
                   1201                                                      1260
VTE2_1     (1197)  GTGATGAGGACAGAACAGCTCCACGGAGGAACTTGAAGTGCCCGGAGTAAC---------
VTE2_2     (1075)  GTGATGAGGACAGAACAGCTCCACGGAGGAACTTGAAGTGCCCGGAGTAAC---------
VTE2_3     (1051)  GTGATGAGGACAGAACAACTCCACGGAGGAACATCA-GTGCCCGGAGTAAAAGCCGCACG
VTE2_4     (1052)  GTG---AGGACAGAACAACTCCACGGAGGAACTTGA-ATGCCCGGAGTAAT---------
VTE2_5     (1139)  GTGATGAGGACAGAACAGCTCCACGGAGGAACTTGAAGTGCCCGGAGTAAC---------
                   1261                                                      1320
VTE2_1     (1248)  ---CCGTTTGAAAACCGAATGGCCACGGACTGCG---CCCAATAAAATTCCCTGTGCAAG
VTE2_2     (1126)  ---CCGTTTGAAAACCGAATGGCCACGGACTGCG---CCCAATAAAATTCCCTGTGCAAG
VTE2_3     (1110)  AATCCGCTTGAAAATCGAATGGCCACGGATTACG---CCCAATAAAATTCCCTGTGCAAG
VTE2_4     (1099)  ---CCGTTTGAAAATCGAATGGCCACGGACTACTACACCCAATAAAATTCCCTGTGCAAG
VTE2_5     (1190)  ---CCGTTTGAAAACCGAATGGCCACGGACTGCG---CCCAATAAAATTCCCTGTGCAAG
```

```
                  1321                                                       1380
VTE2_1  (1302) CCCCCCCCTGGTACATCCCTT-GATATA------TTCCA--GCGAGGTCACTCCCCTGGA
VTE2_2  (1180) CCCCCCCCTGGTACATCCCTT-GATATA------TTCCA--GCGAGGTCACTCCCCTGGA
VTE2_3  (1167) CCCCC----GGTACATCCCTTTGATATA------TTCCA--GCG-------TTCCCTGGC
VTE2_4  (1156) CCCCC----GGTACA-CCCTT-GATATTCCAGCGTTCCCTGGCGAGGTCACT-CACTGGA
VTE2_5  (1244) CCCCCCCCTGGTACATCCCTT-GATATA------TTCCA--GCGAGGTCACTCCCCTGGA
                  1381                                                       1440
VTE2_1  (1353) GAATGAAGATGGACGACCTCCCAACCCCCCACCCCAGAACGTACCCGCGCTGGGGGGGCC
VTE2_2  (1231) GAATGAAGATGGACGACCTCCCAACCCCCCACCCCAGAACGTACCCGCGCTGGGGGGGCC
VTE2_3  (1208) GA--------GGTC--CCTCCCTCCCTCCCTC----------------------------
VTE2_4  (1209) GAATGGAGATGGACGGCCTCCCAACAAACC-CCCCACAATGTACCCAGGCTGGGGGG-CC
VTE2_5  (1295) GAATGAAGATGGACGACCTCCCAACCCCCCACCCCAGAACGTACCCGCGCTGGGGGGGCC
                  1441                                                       1500
VTE2_1  (1413) AGGTGA------------------------------------------------------
VTE2_2  (1291) AGGTGA------------------------------------------------------
VTE2_3  (1230) ------------------------------------------------------------
VTE2_4  (1267) AGGTGAGTGAGCTGTAGAAGTAGTAGGTGCTGTGCTAGCAACAAACTCTTGCAGCAGGAG
VTE2_5  (1355) AGGTGA------------------------------------------------------
                  1501
VTE2_1  (1419) --
VTE2_2  (1297) --
VTE2_3  (1230) --
VTE2_4  (1327) GG
VTE2_5  (1361) --
```

E2

Amino-acid alignment

```
                 1                                                          60
VTE2_1     (1)   MDSLCLRPSAPACAAAAARRRDHILPSFCSIQRSGKGRVSLSIQGSKGPTDDHCKKFLDW
VTE2_2     (1)   ----------------------------------------LSIQGSKGPTVDHCKKFLDW
VTE2_3     (1)   ------------------------------------------------PTVDHCKKFLDW
VTE2_4     (1)   ------------------------------------------------PYC■SL■NFLD■
VTE2_5     (1)   -------------------LIEGYIYHHLFYPAKW■RRVTLSILSIQRPQCISCRKFLDW
                 61                                                        120
VTE2_1    (61)   KYSYHRISHQSRNTSANAGQSLQPETEAHDPASIWKPISS-LDAFYRFSRPHTVIGTALS
VTE2_2    (21)   KYSYHRISHQSRNTSANAGQSLLPETEAHDPASIWKSISSSLDAFYRFSRPHTVIGTALS
VTE2_3    (13)   KYSYHRISHQSRNTSANAGQSLLPETEAHDPASIWKSISSSLDAFYRFSRPHTVIGTALS
VTE2_4    (10)   KYSYHWISHQSRNTSANVGQSLQPETEAHHPASIWKPISSSLDAFYRFSRPHTVIGTALS
VTE2_5    (41)   KYSFHRISHQSRNTSANAGQSLLPETEAHDPASIWKSISSSLDAFYRFSRPHTVIGTALS
                 121                                                       180
VTE2_1   (120)   IVSVSLLAVESLSDISPLFLTGLLEAVVAAFFMNIYIVGLNQLFDIEIDKVNKPTLPLAS
VTE2_2    (81)   IVSVSLLAVESLSDISPLFLTGLLEAVVAAFFMNIYIVGLNQLFDIEIDKVNKPTLPLAS
VTE2_3    (73)   IVSVSLLAVESLSDISPLFLTGLLEAVVAAFFMNIYIVGLNQLFDIEIDKVNKPTLPLAS
VTE2_4    (70)   IVSVSLLAVESLSDISPLFLTGLLEAVVAAFFMNIYIVGLNQVFDIEIDKVNKPTLPLAS
VTE2_5   (101)   IVSVSLLAVESLSDISPLFLTGLLEAVVAAFFMNIYIVGLNQLFDIEIDKVNKPTLPLAS
                 181                                                       240
VTE2_1   (180)   GEYSPATGVAIVSVSAAMSFALGWLVGSPPLFWALFISFVLGTAYSVNLPYFRWKRFAVV
VTE2_2   (141)   GEYSPATGVAIVSVSAAMSFALGWLVGSPPLFWALFISFVLGTAYSVNLPYFRWKRFAVV
VTE2_3   (133)   GEYSPATGVAIVSVSAAMSFALGWLVGSPPLFWALFISFVLGTAYSVNLPYFRWKRFAVV
VTE2_4   (130)   GEYSPATGVAIVSVSAAMSFALGWLVGSPPLFWALFISFVLGTAYSVNLPYFRWKRFAVV
VTE2_5   (161)   GEYSPATGVAIVSVSAAMSFALGWLVGSPPLFWALFISFVLGTAYSVNLPYFRWKRFAVV
                 241                                                       300
VTE2_1   (240)   AALCILAVRAVIVQLAFFLHIQTFVFRRPAVFSKPLIFATAFMTFFSVVIALFKDIPDIE
VTE2_2   (201)   AALCILAVRAVIVQLAFFLHIQTFVFRRPAVFSKPLIFATAFMTFFSVVIALFKDIPDIE
VTE2_3   (193)   AALCILAVRAVIVQLAFFLHIQTFVFRRPAVFSKPLIFATAFMTFFSVVIALFKDIPDIE
VTE2_4   (190)   AALCILAVRAVIVQLAFFLHIQTFVFRRPAVFSKPLIFATAFMTFFSVVIALFKDIPDIE
VTE2_5   (221)   AALCILAVRAVIVQLAFFLHIQTFVFRRPAVFSKPLIFATAFMTFFSVVIALFKDIPDIE
                 301                                                       360
VTE2_1   (300)   GDRIFGIQSFSVRLGQNKVFWICVGLLEMAYGVAILMGATSSSLWSKSVTVAGHAILASV
VTE2_2   (261)   GDRIFGIQSFSVRLGQNKVFWICVGLLEMAYGVAILMGATSSSLWSKSVTVAGHAILASV
VTE2_3   (253)   GDRIFGIQSFSVRLGQNKVFWICVGLLEMAYGVAILMGATSSSLWSKSVTVAGHAILASI
VTE2_4   (250)   GDRIFGIQSFSVRLGQNKVFWICVGLLEMAYGVAILMGATSSSLWSKSVTVAGHAILASL
VTE2_5   (281)   GDRIFGIQSFSVRLGQNKVFWICVGLLEMAYGVAILMGATSSSLWSKSVTVAGHAILASV
                 361                                  398
VTE2_1   (360)   LWSCARSVDLTSKAAITSFYMFIWKLFYAEYLLIPLVR■
VTE2_2   (321)   LWSCARSVDLTSKAAITSFYMFIWKLFYAEYLLIPLVR■
VTE2_3   (313)   LWSCARSVDLTSKAAITSFYMFIWKLFYAEYLLIPLVR■
VTE2_4   (310)   LWSCARSVDLTSKAAITSFYMFIWKLFYAEYLLIPLVR■
VTE2_5   (341)   LWSCARSVDLTSKAAITSFYMFIWKLFYAEYLLIPLVR■
```

M Start codon ■ Stop codon X Identical X Conservative X Similar X Weakly similar X Non similar

F1

VTE3
Nucleotide alignment

```
                 1                                                          60
VTE3_1     (1)   CGCGTCTCCGGAGCCAGGCCCGCGAAGCCGCTGCTGAGGTGCGCGGCGTCGTCCGCGGCT
VTE3_2     (1)   ------------------------------------------------------------
VTE3_3     (1)   ---------------------------------------------------TCCGCGGCG
                 61                                                        120
VTE3_1    (61)   GCGCGGCCCGCCTCGGCGCCGCGCTTCATCCAGCACAAGAAGGAGGCCTTCTGGTTCTAC
VTE3_2     (1)   ------------------------------------------------------------
VTE3_3    (10)   GCGCGGCCCGCCTCCGCGCCGCGCTTCATCCAGCACAAGAAGGAGGCCTTTTGGTTCTAC
                 121                                                       180
VTE3_1   (121)   CGCTTCCTCTCCATCGTGTACGACCACGTCATCAATCCGGGCCACTGGACCGAGGACATG
VTE3_2     (1)   --------------------------------CAACCCGGGCCACTGGACCGAGGACATG
VTE3_3    (70)   CGCTTCCTCTCCATCGTCTACGACCACGTCATCAACCCGGGCCACTGGACCGAGGACATG
                 181                                                       240
VTE3_1   (181)   CGAGACGACGCGCTCGAGCCCGCCGACCTCCACAGCCGCAAGCTCAAGGTCGTCGACGTC
VTE3_2    (29)   CGCGACGACGCGCTCGAGCCCGCCGACCTCCACAGCCGCAAGCTCAAGGTCGTCGACGTC
VTE3_3   (130)   CGCGACGACGCGCTCGAGCCCGCCGACCTCCACAGCCGCAAGCTCAAGGTCGTCGACGTC
                 241                                                       300
VTE3_1   (241)   GGCGGCGGGACCGGGTTCACCACGCTCGGCATCGTCAAGCACGTCGACAAGGAGAACGTC
VTE3_2    (89)   GGCGGCGGGACCGGGTTCACCACGCTCGGCATCGTCAAGCACGTCGACAAAGAGAACGTC
VTE3_3   (190)   GGCGGCGGGACCGGGTTCACCACGCTCGGCATCGTCAAGCACGTCGACAAAGAGAACGTC
                 301                                                       360
VTE3_1   (301)   ATCCTGCTCGACCAGTCCCCGCACCAGCTCGAGAAGGCCAGGCAGAAGGAGGCGCTCAAG
VTE3_2   (149)   ATACTGCTCGACCAGTCCCCGCACCAGCTCGAGAAGGCCAGGCAGAAGGAGGCGCTCAAG
VTE3_3   (250)   ATACTGCTCGACCAGTCCCCGCACCAGCTCGAGAAGGCCAGGCAGAAGGAGGCGCTCAAG
                 361                                                       420
VTE3_1   (361)   GGGGTCGAGATCATGGAGGGCGACGCCGAGGACCTCCCCTTCCCCACAGACACCTTCGAC
VTE3_2   (209)   GGGGTCGAGATCATGGAGGGCGACGCCGAGGACTTGCCATTCCCCACCGATACATTCGAC
VTE3_3   (310)   GGGGTCGAGATCATGGAGGGCGACGCCGAGGACTTGCCATTCCCCACCGATACATTCGAC
                 421                                                       480
VTE3_1   (421)   CGATACGTCTCCGCCGGCAGCATCGAGTACTGGCCTGATCCCCAGCGAGGCATCAAGGAA
VTE3_2   (269)   CGATACGTCTCCGCCGGCAGCATCGAGTACTGGCCTGATCCGCAGCGAGGCATCAAGGAA
VTE3_3   (370)   CGATATGTCTCCGCCGGCAGCATCGAGTACTGGCCTGATCCGCAGCGAGGCATCAAGGAA
                 481                                                       540
VTE3_1   (481)   GCCTACAGGGTCCTGAGGCTCGGCGGGAAGGCTTGTCTGATCGGCCCTGTGCACCCGACC
VTE3_2   (329)   GCCTACAGGGTCCTGAGGCTCGGCGGAAAGGCTTGTCTGATCGGCCCTGTGCACCCGACC
VTE3_3   (430)   GCCTACAGGGTCCTGAGGCTAGGCGGAAAGGCTTGTCTGATCGGCCCTGTGCACCCGACC
                 541                                                       600
VTE3_1   (541)   TTCTGGCTGTCTCGCTTCTTCGCCGACATGTGGATGCTGTTCCCCACTGAAGAGGAGTAC
VTE3_2   (389)   TTCTGGCTGTCTCGCTTCTTCGCCGACATGTGGATGCTGTTTCCCACTGAAGAGGAGTAC
VTE3_3   (490)   TTCTGGCTGTCTCGCTTCTTCGCCGACATGTGGATGCTGTTTCCCACTGAAGAGGAGTAC
                 601                                                       660
VTE3_1   (601)   ATCGAGTGGTTCAAGAACGCAGGGTTCAGGGATGTCCAACTTAAGAGGATTGGACCAAAG
VTE3_2   (449)   ATTGAGTGGTTCAAGAACGCAGGGTTCAGGGATGTCCAACTTAAGAGGATTGGACCAAAG
VTE3_3   (550)   ATTGAGTGGTTCAAGAACGCAGGGTTCAGGGATGTCCAACTTAAGAGGATTGGACCAAAG
                 661                                                       720
VTE3_1   (661)   TGGTACCGCGGTGTCCGTAGGCACGGCCTGATCATGGGATGCTCCGTGACGGGTGTCAAG
VTE3_2   (509)   TGGTACCGTGGTGTCCGTAGGCATGGCCTGATCATGGGATGCTCTGTGACGGGCGTCAAG
VTE3_3   (610)   TGGTACCGTGGTGTCCGTAGGCATGGCCTGATCATGGGATGCTCTGTGACGGGTGTCAAG
                 721                                                       780
VTE3_1   (721)   AGAGAACGTGGGGACTCCCCTTTGCAGCTGGGTCCGAAGGCTGAGGATGTCAGCAAGCCT
VTE3_2   (569)   AGAGAACGTGGGGACTCCCCTTTGCAGCTTGGTCCGAAGGCTGAGGATGTCAGCAAGCCT
VTE3_3   (670)   AGAGAACGTGGGGACTCCCCTTTGCAGCTTGGTCCGAAGGCTGAGGATGTCAGCAAGCCT
                 781                                                       840
VTE3_1   (781)   GTGAATCCTATCACTTTCTTCTTCTGCTTCCTCATGGGAACGATATGTGCTGCATACTAT
VTE3_2   (629)   GTGAACCCTATCACTTTCTTCTTCCGCTTCCTCATGGGAACGATATGTGCCGCATACTAT
VTE3_3   (730)   GTGAACCCTATCACTTTCTTCTTCCGCTTCCTCATGGGAACGATATGTGCCGCATACTAT
```

```
                841                                                        900
VTE3_1    (841) GTTCTGGTGCCTATTTACATGTGGATAAAGGACCAGATTGTGCCCAAAGGCCAGCCAATC
VTE3_2    (689) GTTCTGGTGCCTATTTACATGTGGATAAAGGACCAGATTGTGCCCAAAGGCCAGCCAATC
VTE3_3    (790) GTTCTGGTGCCTATTTACATGTGGATAAAGGACCAGATTGTGCCCAAAGGCCAGCCAATC
                901                                                        960
VTE3_1    (901) TAAGGGAGAGGAATTAAGCTGCAGCAAAGAGTGGATAGCCAGTATCTGAATTAGGCTTCT
VTE3_2    (749) TAAGGGAGAGGAATTAAGCTGCAGCGAAGAGTGGATAGCCAGTATCTGAATTAGGCTTCT
VTE3_3    (850) TAAGGGAGAGGAATTAAGCTGCAGCGAAGAGTGGATAGCCAGTATCTGAATTAGGCTTCT
                961                                                       1020
VTE3_1    (961) CTATATTAGGCACTCCACGCCTGTTTTTTGTTTATCTTTATATTTCCTGTCGTCGTGTAT
VTE3_2    (809) CTATATTAGGCACTCCACACCTGTTTTT-GTTTATCTTTA--------------------
VTE3_3    (910) CTATATTAGGC-------------------------------------------------
                1021                                                      1080
VTE3_1   (1021) TTATTTTTGCTGCGGTTCTTGTTTCCATTGAGAAATAATATTAAAGACTTTGATTATGCT
VTE3_2    (848) ------------------------------------------------------------
VTE3_3    (921) ------------------------------------------------------------
                1081                          1111
VTE3_1   (1081) CGGACGGCGAGTGTATATTTTTTCTGACGTA
VTE3_2    (848) -------------------------------
VTE3_3    (921) -------------------------------
```

F2

Amino-acid alignment

```
                1                                                          60
VTE3_1      (1) RVSGARPAKPLLRCAASSAAARPASAPRFIQHKKEAFWFYRFLSIVYDHVINPGHWTEDM
VTE3_2      (1) ---------------------------------------------------NPGHWTEDM
VTE3_3      (1) -----------------SAAARPASAPRFIQHKKEAFWFYRFLSIVYDHVINPGHWTEDM
                61                                                        120
VTE3_1     (61) RDDALEPADLHSRKLKVVDVGGGTGFTTLGIVKHVDKENVILLDQSPHQLEKARQKEALK
VTE3_2     (10) RDDALEPADLHSRKLKVVDVGGGTGFTTLGIVKHVDKENVILLDQSPHQLEKARQKEALK
VTE3_3     (44) RDDALEPADLHSRKLKVVDVGGGTGFTTLGIVKHVDKENVILLDQSPHQLEKARQKEALK
                121                                                       180
VTE3_1    (121) GVEIMEGDAEDLPFPTDTFDRYVSAGSIEYWPDPQRGIKEAYRVLRLGGKACLIGPVHPT
VTE3_2     (70) GVEIMEGDAEDLPFPTDTFDRYVSAGSIEYWPDPQRGIKEAYRVLRLGGKACLIGPVHPT
VTE3_3    (104) GVEIMEGDAEDLPFPTDTFDRYVSAGSIEYWPDPQRGIKEAYRVLRLGGKACLIGPVHPT
                181                                                       240
VTE3_1    (181) FWLSRFFADMWMLFPTEEEYIEWFKNAGFRDVQLKRIGPKWYRGVRRHGLIMGCSVTGVK
VTE3_2    (130) FWLSRFFADMWMLFPTEEEYIEWFKNAGFRDVQLKRIGPKWYRGVRRHGLIMGCSVTGVK
VTE3_3    (164) FWLSRFFADMWMLFPTEEEYIEWFKNAGFRDVQLKRIGPKWYRGVRRHGLIMGCSVTGVK
                241                                                       300
VTE3_1    (241) RERGDSPLQLGPKAEDVSKPVNPITFFFCFLMGTICAAYYVLVPIYMWIKDQIVPKGQPI
VTE3_2    (190) RERGDSPLQLGPKAEDVSKPVNPITFFFRFLMGTICAAYYVLVPIYMWIKDQIVPKGQPI
VTE3_3    (224) RERGDSPLQLGPKAEDVSKPVNPITFFFRFLMGTICAAYYVLVPIYMWIKDQIVPKGQPI
                301
VTE3_1    (301) ■
VTE3_2    (250) ■
VTE3_3    (284) ■
```

M Start codon ■ Stop codon X Identical X Conservative X Similar X Weakly similar X Non similar

G1

VTE4
Nucleotide alignment

```
                   1                                                          60
VTE4_1      (1)  GAGGCCCCGATTACAAAAATGGCCAACTCCGCCGCCCTGCTCCACTCACTCCCCTCCACT
VTE4_2      (1)  ----------------AAATGGCCAACTCCACCGCCCTGCTCCACTCACTCCCCTCCACT
VTE4_3      (1)  ----------------AAATGGCCAACTCCACCGCCCTGCTCCACTCACTCCCCTCCACT
                 61                                                          120
VTE4_1     (61)  GCCTCGACCCCGCGCCTCCGCCGCCGAGCCTCGGCCACGCAACTCGCCCCGTCCGCCGGC
VTE4_2     (45)  GCCTCGACCCCGCGCCTCCGCCGCCGAGCCTCGGCCACGCAGCCCGCCCCGTCCGCCGGC
VTE4_3     (45)  GCCTCGACCCCGCGCCTCCGCCGCCGAGCCTCGGCCACGCAGCCCGCCCCGTCCGCCGGC
                 121                                                         180
VTE4_1    (121)  CTTTTCTGCCGCCGGCCGACGTTCGCCGCCCGCCCGATGGCGTCGGCGATGACCGCTGCC
VTE4_2    (105)  CTTTTCTGCCGCCGGCCGACGCTCGCGCCCCGCCCGATGGCGTCGGCGACGACCGCTGCC
VTE4_3    (105)  CTTTTCTGCCGCCGGCCGACGCTCGCGCCCCGCCCGATGGCGTCGGCGACGACCGCTGCC
                 181                                                         240
VTE4_1    (181)  CTGGCCGACGCGTCGGCGCCGCCGGGGCTGAACGAGGGCATCGCGGGGCTCTACGACGAG
VTE4_2    (165)  CTGGCCAACGCGTCGGCGCCGCCAGGGCTGAACGAGGGCATCGCGGGCCTCTACGACGAG
VTE4_3    (165)  CTGGCCAACGCGTCGGCGCCGCCAGGGCTGAACGAGGGCATCGCGGGCCTCTACGACGAG
                 241                                                         300
VTE4_1    (241)  TCGTCCGGCCTGTGGGAGAACATCTGGGGCGAGCACATGCACCACGGCTTCTACGACTCC
VTE4_2    (225)  TCGTCCGGCCTGTGGGAGAACATCTGGGGCGACCACATGCACCACGGCTTCTACGACTCC
VTE4_3    (225)  TCGTCCGGCCTGTGGGAGAACATCTGGGGCGAGCACATGCACCACGGCTTCTACGACTCC
                 301                                                         360
VTE4_1    (301)  GGCGAGGCCGCCGACATGTCCGACCACCGCCGCGCCCAGATCCGCATGATCGAGGAGGCA
VTE4_2    (285)  GGCGAGGCCGCCGACATGTCCGACCACCGCCGCGCCCAGATCCGCATGATCGAGGAGGCG
VTE4_3    (285)  GGCGAGGCCGCCGACATGTCCGACCACCGCCGCGCCCAGATCCGCATGATCGAGGAGGCG
                 361                                                         420
VTE4_1    (361)  CTCGCCTTCGCCGCCGTTCCGGACGATCCGGCGAACAAACCCAAAACGATAGTGGATGTT
VTE4_2    (345)  CTCGCCTTCGCTGCCGTTCCGGACGATCCGGCAAACAAACCCAAAACGATAGTGGATGTT
VTE4_3    (345)  CTCGCCTTCGCTGCCGTTCCGGACAATCCGGCAAACAAACCCAAAACGATAGTGGATGTT
                 421                                                         480
VTE4_1    (421)  GGGTGTGGAATCGGCGGTAGCTCAAGATACCTGGCGAACAAATATGGAGCACAGTGCCGC
VTE4_2    (405)  GGGTGTGGAATCGGCGGTAGCTCAAGGTACCTGGCGAACAAATATGGAGCACAGTGCCGC
VTE4_3    (405)  GGGTGTGGAATCGGCGGTAGCTCAAGGTACCTGGCGAACAAATATGGAGCACAGTGCCGC
                 481                                                         540
VTE4_1    (481)  GGGATCACACTGAGCCCTGTGCAAGCCGAGAGAGGAAATGCCCTCGCGGCGGCACAGGGG
VTE4_2    (465)  GGGATCACACTGAGTCCTGTGCAAGCTGAGAGAGGAAATGCCCTCGCGGCAGCACAGGGG
VTE4_3    (465)  GGGATCACACTGAGTCCTGTGCAAGCCGAGAGAGGAAATGCCCTCGCGGCAGCACAGGGG
                 541                                                         600
VTE4_1    (541)  TTGTCGGATAAGGCTTCTTTCCAAGTTGCTGATGCTCTGGAGCAACCTTTTCCCGATGGT
VTE4_2    (525)  TTGTCGGATAAGGCTTCTTTCCAAGTTGCTGATGCTCTGGAGCAACCTTTTCCCGATGGT
VTE4_3    (525)  TTGTCGGATAAGGCTTCTTTCCAAGTTGCTGATGCTCTGGAGCAACCTTTTCCCGATGGT
                 601                                                         660
VTE4_1    (601)  CAGTTTGATCTGGTTTGGTCTATGGAGAGTGGTGAGCACATGCCGAACAAACGGAAGTTT
VTE4_2    (585)  CAGTTTGATCTAGTTTGGTCTATGGAGAGTGGTGAGCACATGCCGAACAAACAGAAGTTT
VTE4_3    (585)  CAGTTTGATCTAGTTTGGTCTATGGAGAGTGGTGAGCACATGCCGAACAAACAGAAGTTT
                 661                                                         720
VTE4_1    (661)  GTAGGTGAGCTGGCACGAGTTGCAGCTCCTGGAGCGAAGATTATCATCGTGACCTGGTGC
VTE4_2    (645)  GTAGGTGAGCTGGCACGCGTCGCAGCTCCTGGAGCGACAATCATCATCGTGACCTGGTGC
VTE4_3    (645)  GTAGGTGAGCTGGCACGCGTCGCAGCTCCTGGAGCGACAATCATCATCGTGACCTGGTGC
                 721                                                         780
VTE4_1    (721)  CATAGGAACCTAGCGCCATCTGAAGACTCACTGAAACCTGACGAGTTGAATCTCTTGAAA
VTE4_2    (705)  CATAGGAACCTAGCGCCGTCTGAAGACTCACTGAAACCTGACGAGCTGAATCTCTTGAAA
VTE4_3    (705)  CATAGGAACCTAGCGCCGTCTGAAGACTCACTGAAACCTGACGAGCTGAATCTCCTGAAA
                 781                                                         840
VTE4_1    (781)  AAGATTTGTGATGCATATTACCTCCCAGATTGGTGTTCTCCTTCCGATTATGTCAAGATT
VTE4_2    (765)  AAGATCTGTGATGCATATTATCTCCCAGATTGGTGCTCCCCTTCTGATTATGTCAAGATT
VTE4_3    (765)  AAGATCTGTGATGCATACTATCTCCCAGATTGGTGCTCCCCTTCTGATTATGTCAAGATT
```

```
                  841                                                          900
VTE4_1   (841)  GCCGAGTCATTGTCCCTTGAGGATATCAAGACGGCGGACTGGTCGGAAAACGTGGCTCCC
VTE4_2   (825)  GCCAAGTCACTGTCCCTCGAGGATATCAAGACGGCTGACTGGTCAGAAAACGTGGCTCCG
VTE4_3   (825)  GCCAAGTCACTGTCCCTCGAGGATATCAAGACGGCTGACTGGTCAGAAAACGTGGCTCCG
                901                                                          960
VTE4_1   (901)  TTTTGGCCTGCTGTCATTCAATCAGCACTGACTTGGAAAGGGCTCACTTCTCTACTAAGA
VTE4_2   (885)  TTTTGGCCCGCTGTCATCCAATCAGCACTCACATGGAAAGGACTCACCTCTCTACTAAGA
VTE4_3   (885)  TTTTGGCCCGCTGTCATCCAATCAGCACTCACATGGAAAGGACTCACCTCTCTACTAAGA
                961                                                         1020
VTE4_1   (961)  AGTGGATGGAAGACGATAAAAGGAGCTCTGGTGATGCCTCTCATGATTCAAGGCTACAAG
VTE4_2   (945)  AGTGGATGGAAGACGATAAAGGGAGCACTGGTGATGCCTCTCATGATTCAAGGGTACAAG
VTE4_3   (945)  AGTGGATGGAAGACGATAAAGGGAGCACTGGTGATGCCTCTCATGATTCAAGGGTACAAG
                1021                                                        1080
VTE4_1  (1021)  AAAGGCCTCATCAAGTTCACCATCATCACCTGCCGCAAACCCCAAGCAGCCATAGAAGGA
VTE4_2  (1005)  AAAGGCCTCATCAAGTTCACCATCATCACCTGCCGCAAACCCCAAGCAGCCATAGAAGGA
VTE4_3  (1005)  AAAGGCCTCATCAAGTTCACCATCATCACCTGCCGCAAACCCCAAGCAGCCATAGAAGGA
                1081                                                        1140
VTE4_1  (1081)  GAGGAAACTGCCATCGCATCCCCCAGCATAGAATGATAGAACCGATGTGATTGCGATTTG
VTE4_2  (1065)  GAGGAAACTCCCATCGCATCCCCCAGCATAGAATGATAGAACCCATGTGATTGCGATTTG
VTE4_3  (1065)  GAGGAAACTCCCATCGCATCCCCCAGCATAGAATGATAGAACCCATGTGATTGCGATTTG
                1141                                                        1200
VTE4_1  (1141)  TCCATCGTAATAGAGTCGGCATGCTGTTGCCTT---------------------------
VTE4_2  (1125)  TACATCGTAATAGACTCGGCATGTTATTATATTAGGCACTCCACGCCTGTCTTTGTAATC
VTE4_3  (1125)  TACATCGTAATAGAGTC-------------------------------------------
                1201     1213
VTE4_1  (1174)  -------------
VTE4_2  (1185)  TTAATGCCGCCCA
VTE4_3  (1142)  -------------
```

G2

Amino-acid alignment

```
                1                                                          60
VTE4_1     (1)  MANSAALLHSLPSTASTPRLRRRASATQLAPSAGLFCRRPTFAARPMASAMTAALADASA
VTE4_2     (1)  MANSTALLHSLPSTASTPRLRRRASATQPAPSAGLFCRRPTLAPRPMASATTAALANASA
VTE4_3     (1)  MANSTALLHSLPSTASTPRLRRRASATQPAPSAGLFCRRPTLAPRPMASATTAALANASA
                61                                                        120
VTE4_1    (61)  PPGLNEGIAGLYDESSGLWENIWGEHMHHGFYDSGEAADMSDHRRAQIRMIEEALAFAAV
VTE4_2    (61)  PPGLNEGIAGLYDESSGLWENIWGDHMHHGFYDSGEAADMSDHRRAQIRMIEEALAFAAV
VTE4_3    (61)  PPGLNEGIAGLYDESSGLWENIWGEHMHHGFYDSGEAADMSDHRRAQIRMIEEALAFAAV
                121                                                       180
VTE4_1   (121)  PDDPANKPKTIVDVGCGIGGSSRYLANKYGAQCRGITLSPVQAERGNALAAAQGLSDKAS
VTE4_2   (121)  PDDPANKPKTIVDVGCGIGGSSRYLANKYGAQCRGITLSPVQAERGNALAAAQGLSDKAS
VTE4_3   (121)  PDNPANKPKTIVDVGCGIGGSSRYLANKYGAQCRGITLSPVQAERGNALAAAQGLSDKAS
                181                                                       240
VTE4_1   (181)  FQVADALEQPFPDGQFDLVWSMESGEHMPNKRKFVGELARVAAPGAKIIIVTWCHRNLAP
VTE4_2   (181)  FQVADALEQPFPDGQFDLVWSMESGEHMPNKQKFVGELARVAAPGATIIIVTWCHRNLAP
VTE4_3   (181)  FQVADALEQPFPDGQFDLVWSMESGEHMPNKQKFVGELARVAAPGATIIIVTWCHRNLAP
                241                                                       300
VTE4_1   (241)  SEDSLKPDELNLLKKICDAYYLPDWCSPSDYVKIAESLSLEDIKTADWSENVAPFWPAVI
VTE4_2   (241)  SEDSLKPDELNLLKKICDAYYLPDWCSPSDYVKIAKSLSLEDIKTADWSENVAPFWPAVI
VTE4_3   (241)  SEDSLKPDELNLLKKICDAYYLPDWCSPSDYVKIAKSLSLEDIKTADWSENVAPFWPAVI
                301                                                       360
VTE4_1   (301)  QSALTWKGLTSLLRSGWKTIKGALVMPLMIQGYKKGLIKFTIITCRKPQAAIEGEETAIA
VTE4_2   (301)  QSALTWKGLTSLLRSGWKTIKGALVMPLMIQGYKKGLIKFTIITCRKPQAAIEGEETPIA
VTE4_3   (301)  QSALTWKGLTSLLRSGWKTIKGALVMPLMIQGYKKGLIKFTIITCRKPQAAIEGEETPIA
                361
VTE4_1   (361)  SPSIE■
VTE4_2   (361)  SPSIE■
VTE4_3   (361)  SPSIE■
```

M Start codon ■ Stop codon X Identical X Conservative X Similar X Weakly similar X Non similar

Figure S2. Nucleotide (1) and amino-acid (2) alignment of the three oat homeologs. A) HGGT B) GGR C) HPPD D) VTE1 E) VTE2 F) VTE3 and G) VTE4. Arrows on top of nucleotide sequence indicate starting codon (green) and stop codon (red), respectively.
