# Supplementary material for: Subgenome‐specific assembly of vitamin E biosynthesis genes and expression patterns during seed development provide insight into the evolution of oat genome
Source: Plant Biotechnol J. 2016 May 26;14(11):2147–57. doi: 10.1111/pbi.12571 (PMC5096403; doi:10.1111/pbi.12571)
Supplement: Supplementary file 3 — Figure S3. Maximum likelihood phylogenetic trees. [file PBI-14-2147-s009.pdf]

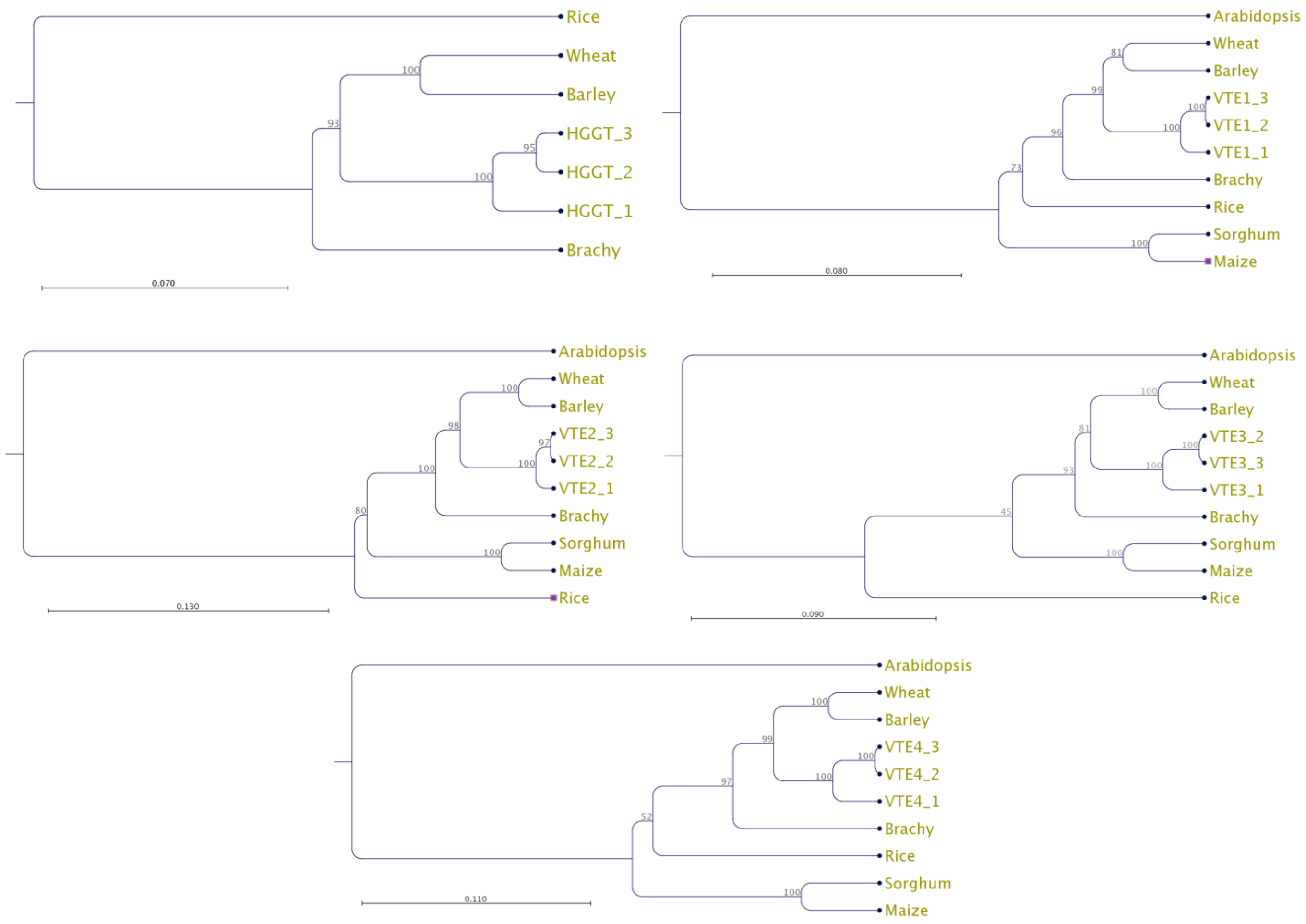

Figure S3. Maximum likelihood (ML) phylogenetic trees constructed with the nucleotide sequence alignments of the *A. sativa* homeologs and the homologous cds of close relatives. ML trees were constructed based on expectation maximization on substitution model parameters and branch length optimization (PHYML). Numbers on diverging points show bootstrap calculation on 1000 replicates. Branch lengths are given in terms of expected numbers of substitutions per nucleotide site.
