# Supplementary material for: Subgenome‐specific assembly of vitamin E biosynthesis genes and expression patterns during seed development provide insight into the evolution of oat genome
Source: Plant Biotechnol J. 2016 May 26;14(11):2147–57. doi: 10.1111/pbi.12571 (PMC5096403; doi:10.1111/pbi.12571)
Supplement: Supplementary file 4 — Figure S4. Amino‐acid alignment of oat homeolog proteins with orthologous proteins from close relatives. [file PBI-14-2147-s008.pdf]

A

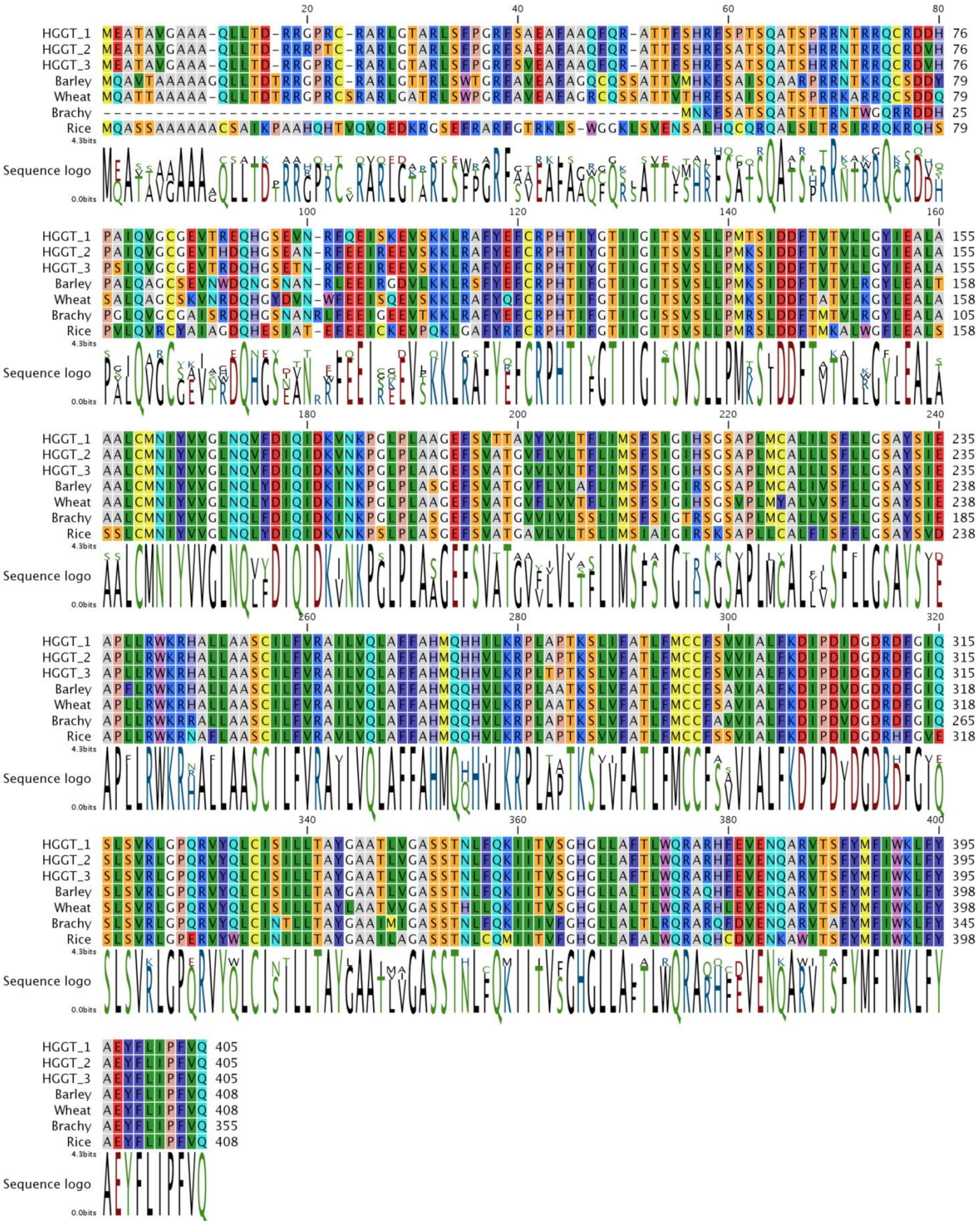

20
40
60
80
HGGT_1
HGGT_2
HGGT_3
Barley
Wheat
Brachy
Rice
Sequence logo
4.3bits
0.0bits

B

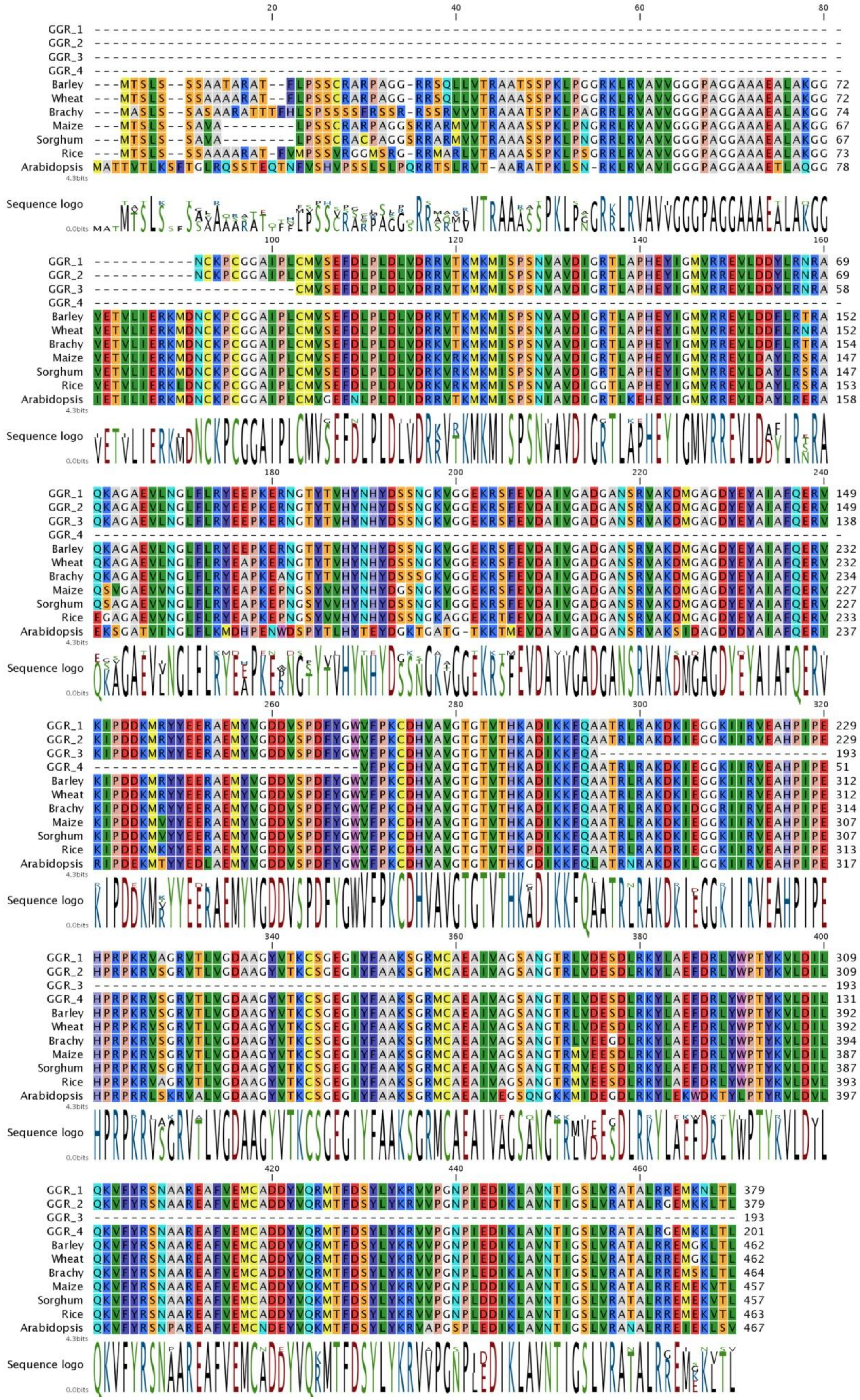
GGR_1
GGR_2
GGR_3
GGR_4
Barley
Wheat
Brachy
Maize
Sorghum
Rice
Arabidopsis
Sequence logo

C

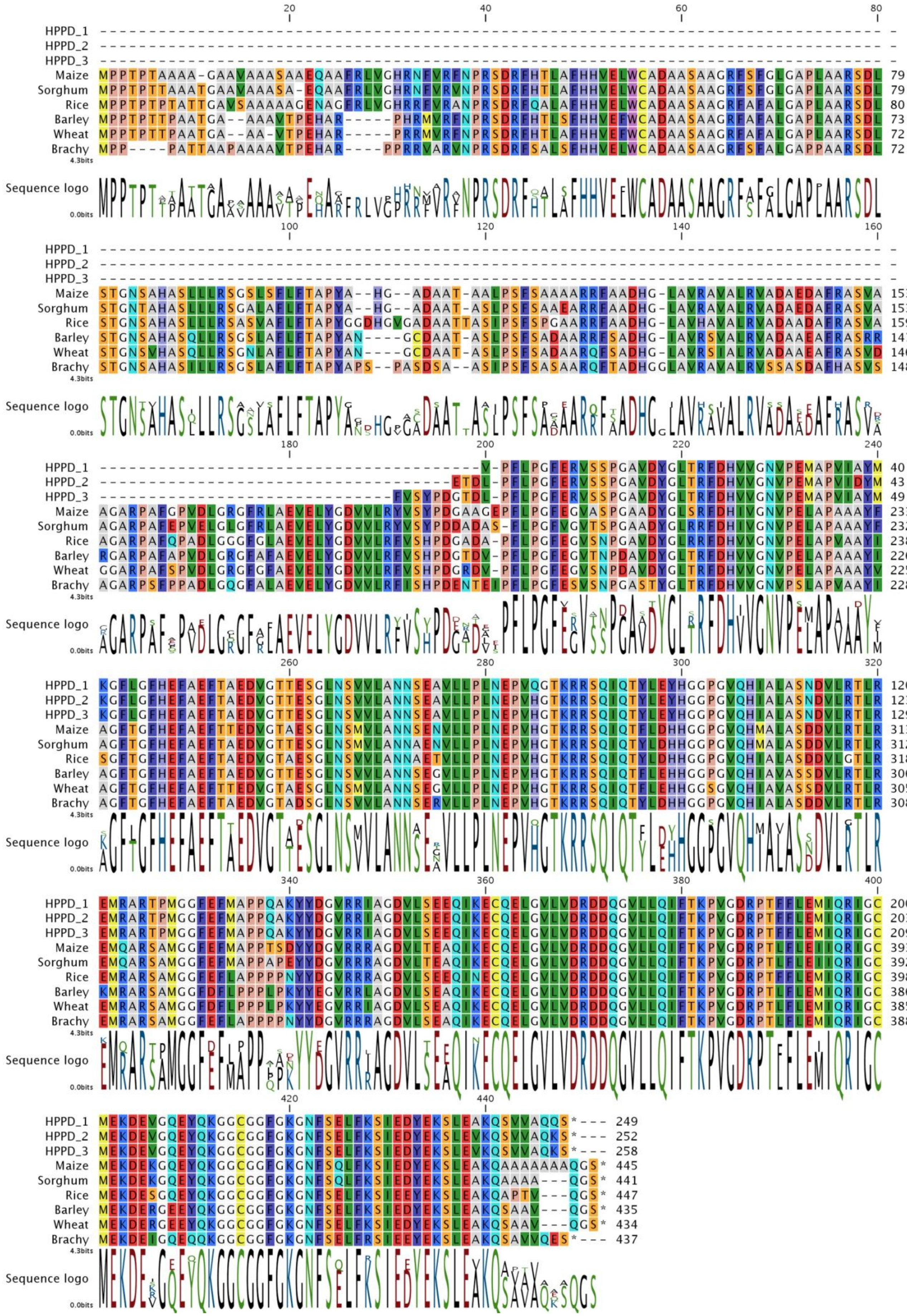
HPPD_1
HPPD_2
HPPD_3
Maize
Sorghum
Rice
Barley
Wheat
Brachy
Sequence logo
4.3bits
0.0bits

D

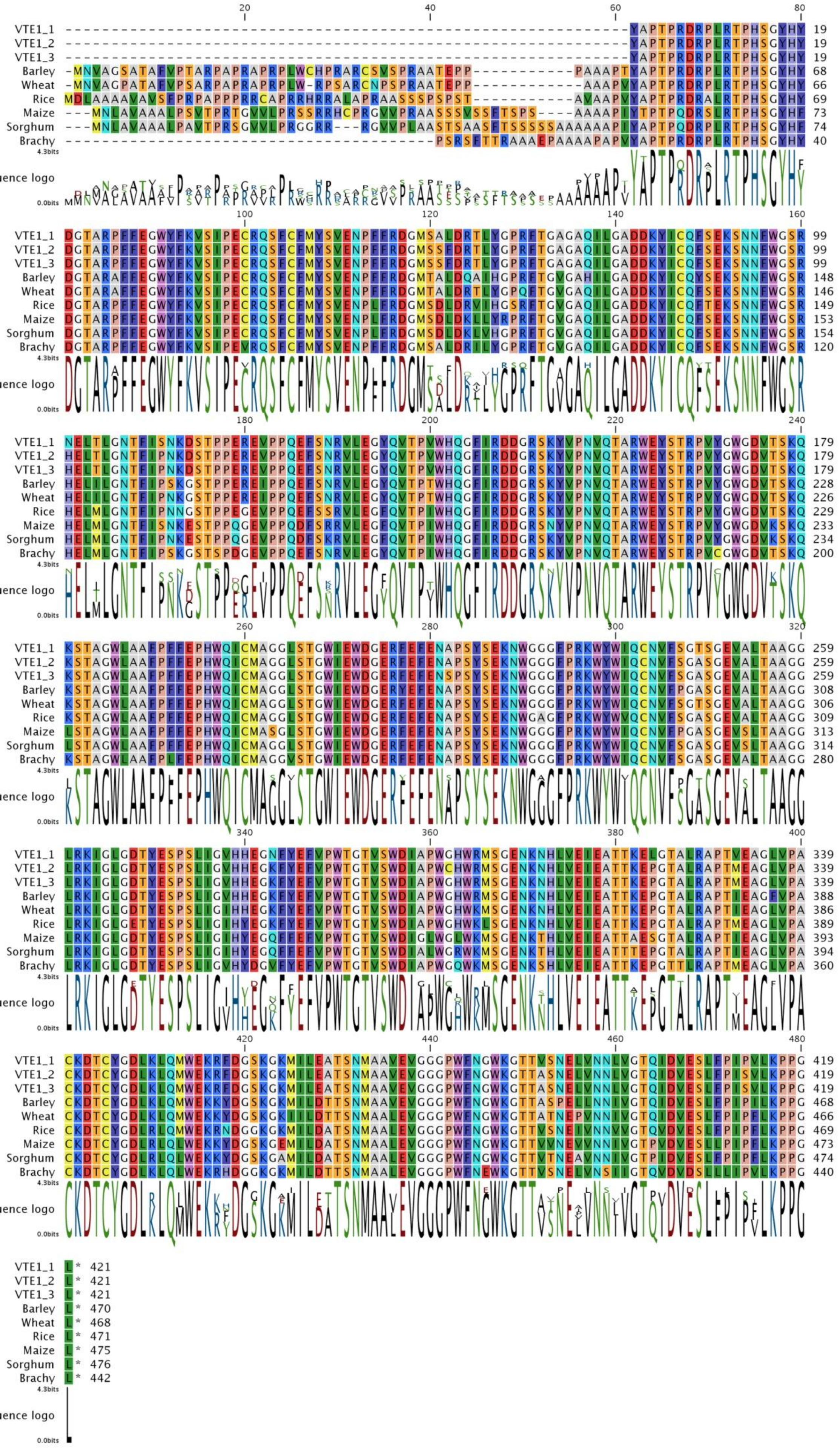
VTE1_1
VTE1_2
VTE1_3
Barley
Wheat
Rice
Maize
Sorghum
Brachy
Sequence logo

E

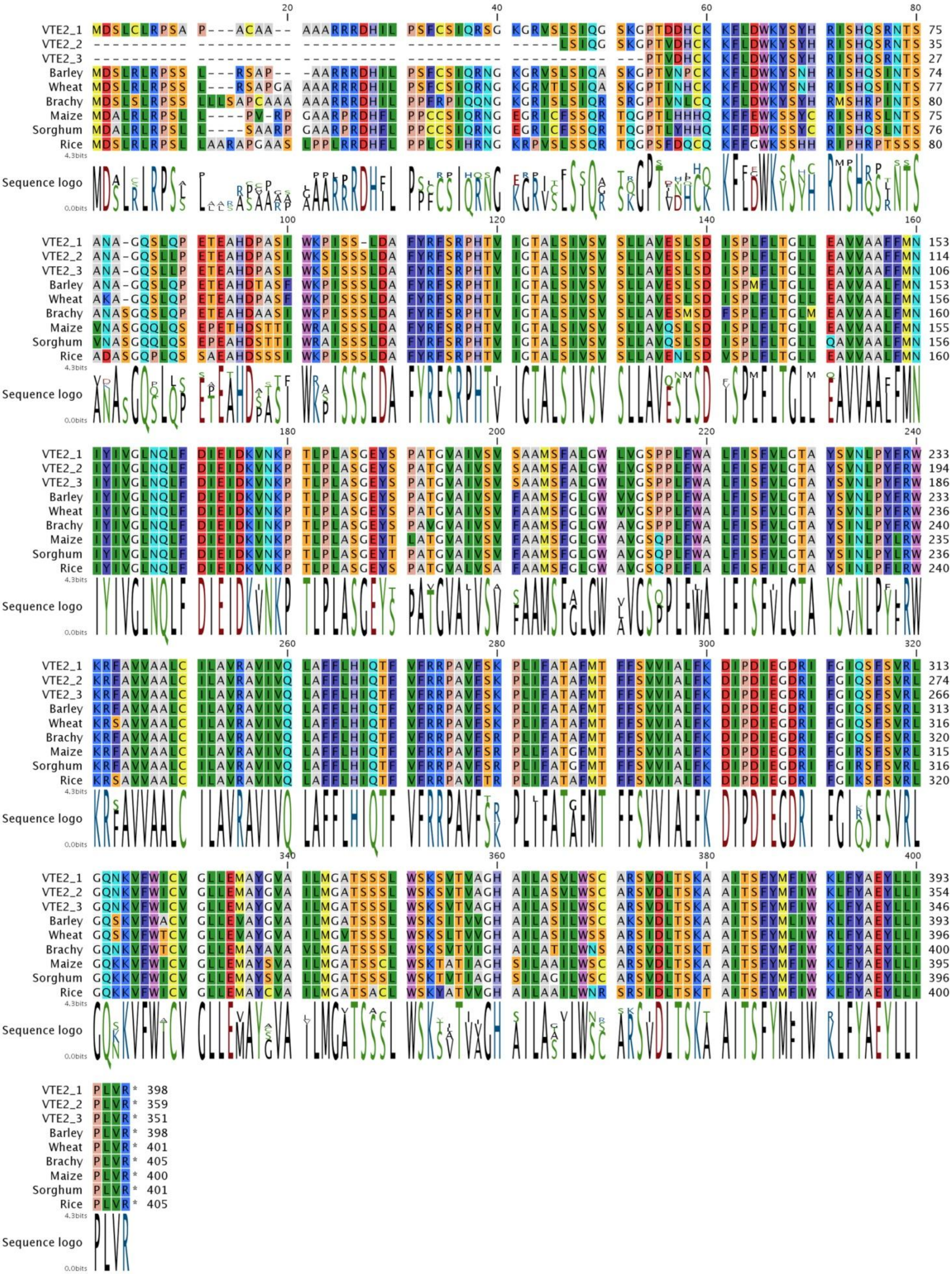
VTE2_1
VTE2_2
VTE2_3
Barley
Wheat
Brachy
Maize
Sorghum
Rice
Sequence logo

F

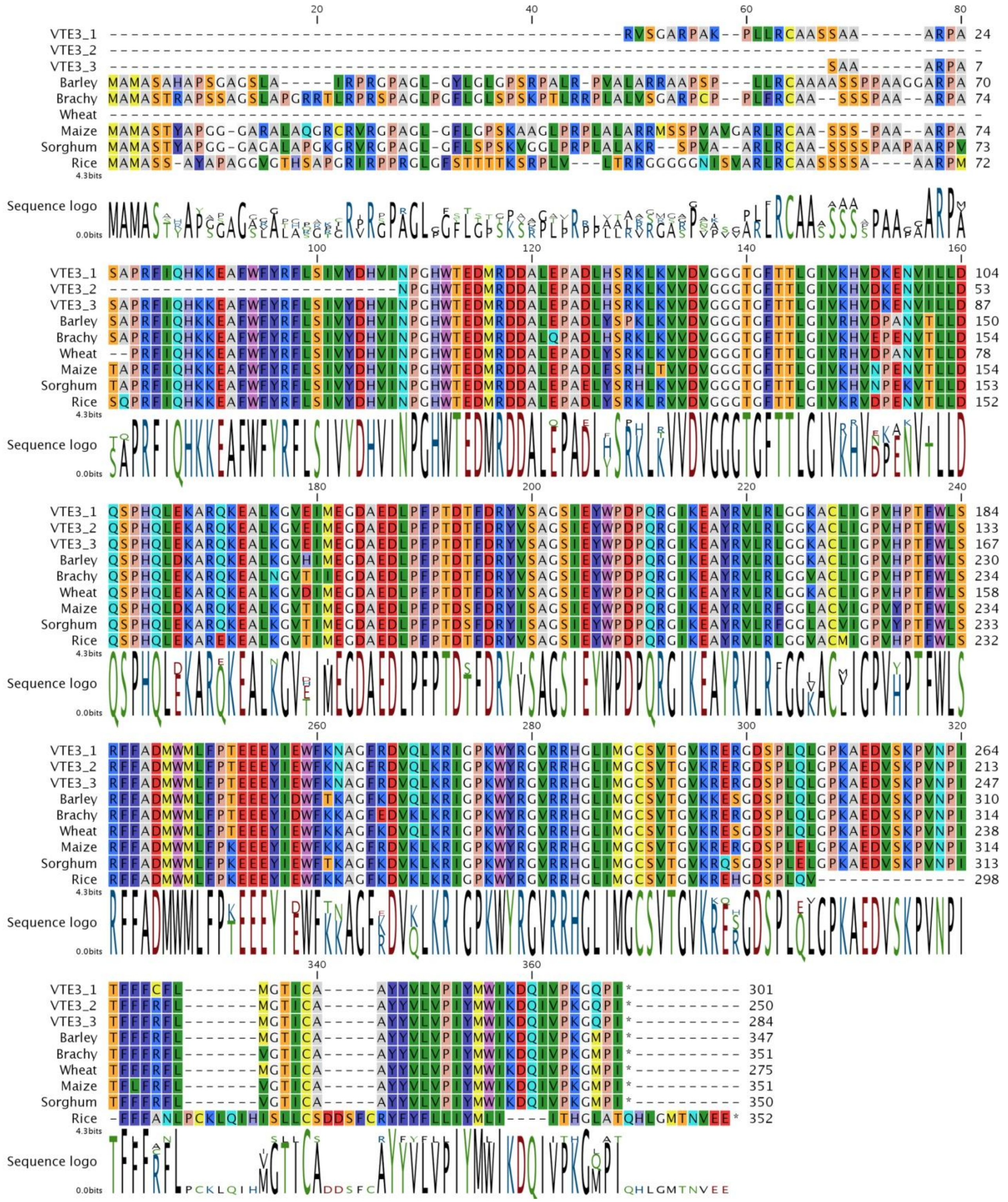
VTE3_1
VTE3_2
VTE3_3
Barley
Brachy
Wheat
Maize
Sorghum
Rice
Sequence logo
4.3bits
0.0bits

G

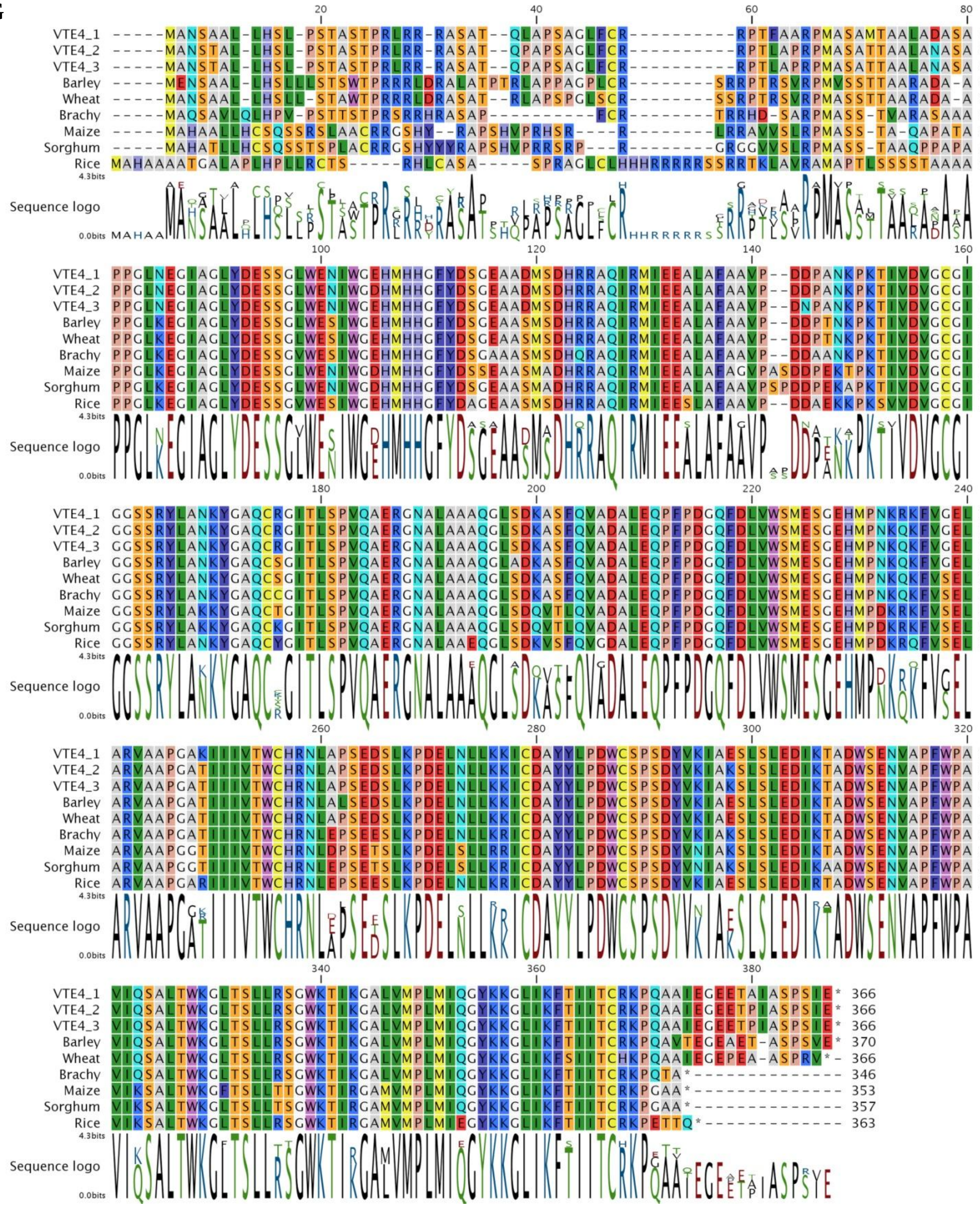

Figure S4. Amino-acid alignment of the three oat homeolog proteins with orthologous proteins from close relatives. Residues in the logo representations are color-coded by polarity: non-polar side chains (black), polar (green), negative (-) electrical charge (red), and positive (+) electrical charge (blue). A) HGGT B) GGR C) HPPD D) VTE1 E) VTE2 F) VTE3 and G) VTE4.
