# Supplementary material for: Subgenome‐specific assembly of vitamin E biosynthesis genes and expression patterns during seed development provide insight into the evolution of oat genome
Source: Plant Biotechnol J. 2016 May 26;14(11):2147–57. doi: 10.1111/pbi.12571 (PMC5096403; doi:10.1111/pbi.12571)
Supplement: Supplementary file 5 — Figure S5. Multidimensional scaling (MDS) plot of distances. [file PBI-14-2147-s007.pdf]

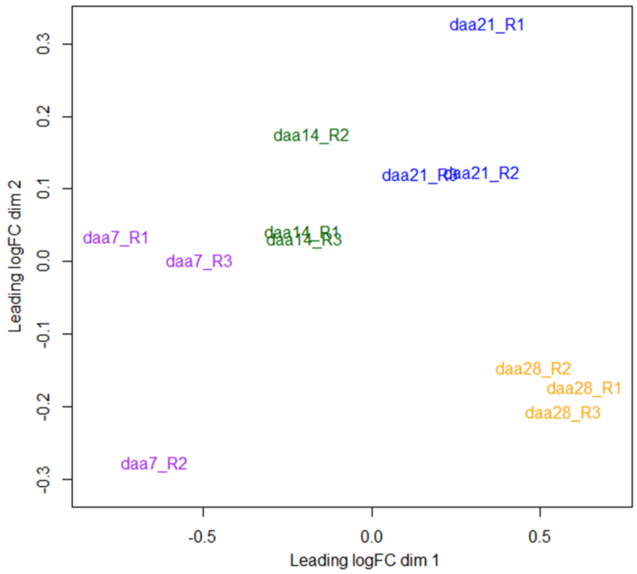

Figure S5. Multidimensional scaling (MDS) plot of distances between digital expression profiles showing the relations between all pairs of samples.
