# Supplementary figures and images for: Subgenome‐specific assembly of vitamin E biosynthesis genes and expression patterns during seed development provide insight into the evolution of oat genome
Source: Plant Biotechnol J. 2016 May 26;14(11):2147–57. doi: 10.1111/pbi.12571 (PMC5096403; doi:10.1111/pbi.12571)

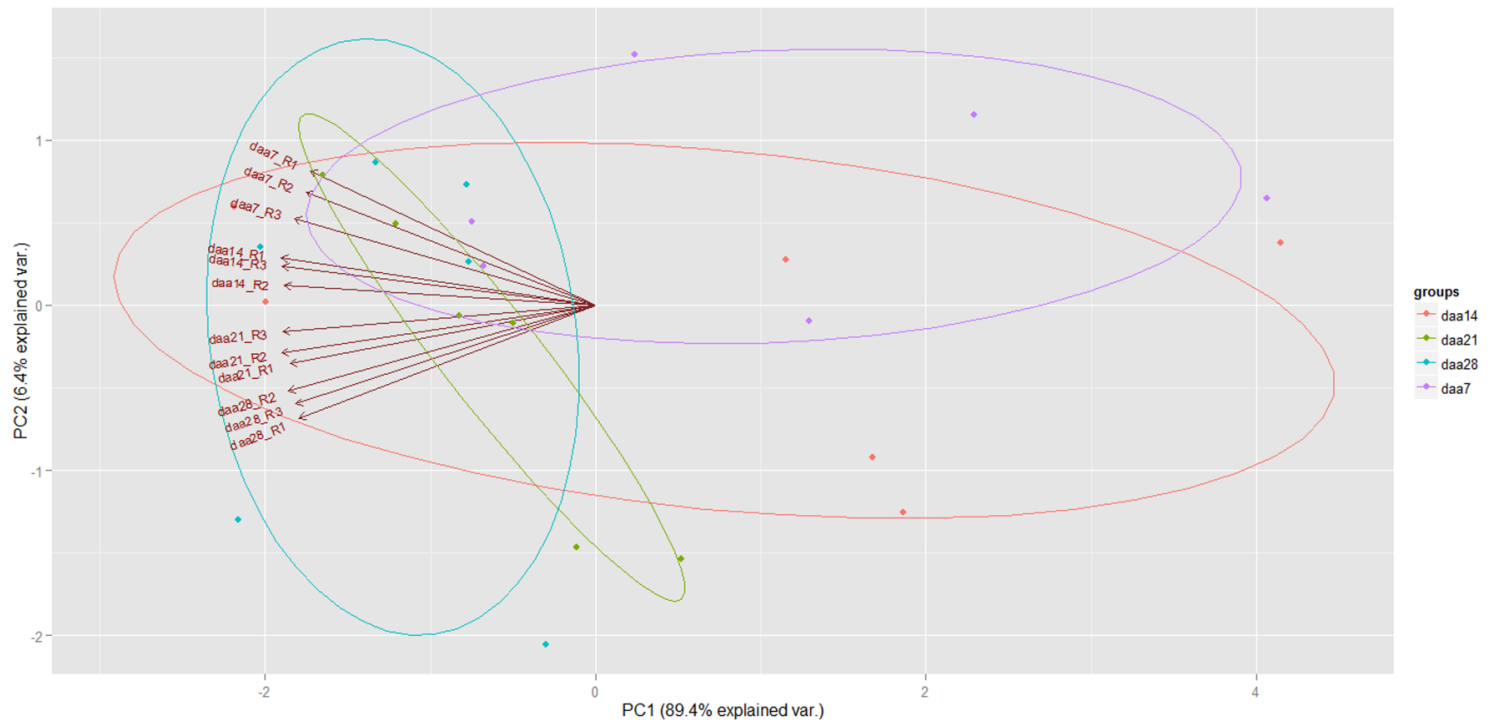

**Figure S6.** PCA plot projecting the data on the first two PCs to inspect sample relationships.

Supplement: Supplementary file 6 — Figure S6. PCA plot projecting the data on the first two PCs. [file PBI-14-2147-s006.pdf]
