# Supplementary material for: Subgenome‐specific assembly of vitamin E biosynthesis genes and expression patterns during seed development provide insight into the evolution of oat genome
Source: Plant Biotechnol J. 2016 May 26;14(11):2147–57. doi: 10.1111/pbi.12571 (PMC5096403; doi:10.1111/pbi.12571)
Supplement: Supplementary file 7 — Figure S7. Heat map showing distances between replicated samples and developmental stages. [file PBI-14-2147-s001.pdf]

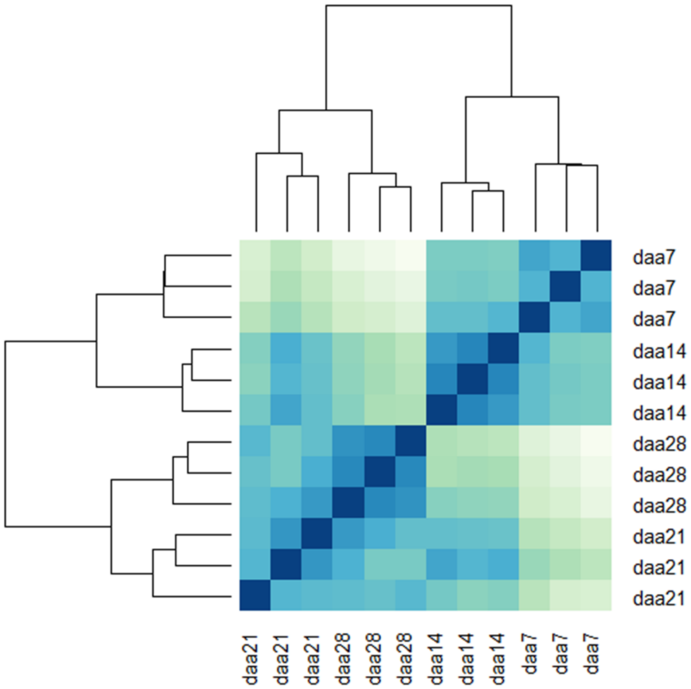

Figure S7. Heatmap showing the Euclidian distances between replicated samples and developmental stages sampled. Variance stabilization transformed counts were used to explore samples similarities and dissimilarities. Samples taken at oat seed developmental stages: 7, 14, 21, and 28 days after anthesis: daa7, daa14, daa21 and daa28, respectively.
