# Supplementary material for: Subgenome‐specific assembly of vitamin E biosynthesis genes and expression patterns during seed development provide insight into the evolution of oat genome
Source: Plant Biotechnol J. 2016 May 26;14(11):2147–57. doi: 10.1111/pbi.12571 (PMC5096403; doi:10.1111/pbi.12571)
Supplement: Supplementary file 8 — Figure S8. Heat map of expression profiles standardized to the mean. [file PBI-14-2147-s002.pdf]

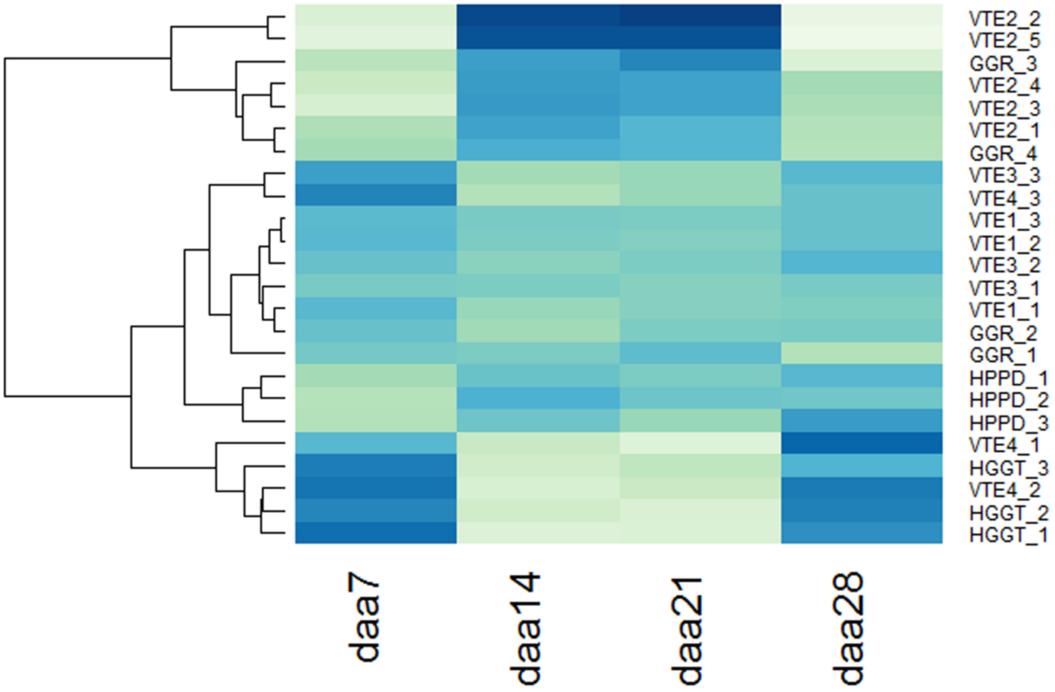

Figure S8. Developmental expression profiles of vitamin E biosynthesis genes in oat seeds. Heatmap shows variance stabilization transformed homeolog normalized expression count values for the average of the three replicates per stage. Expression levels were standardized to the mean. Samples taken at the grain developmental stages 7, 14, 21, and 28 days after anthesis: daa7, daa14, daa21 and daa28, respectively. Darker blue color implies higher expression.
