# Supplementary material for: Subgenome‐specific assembly of vitamin E biosynthesis genes and expression patterns during seed development provide insight into the evolution of oat genome
Source: Plant Biotechnol J. 2016 May 26;14(11):2147–57. doi: 10.1111/pbi.12571 (PMC5096403; doi:10.1111/pbi.12571)
Supplement: Supplementary file 9 — Table S1. Accumulation of individual tocopherols and tocotrienols in the seeds of oats during seed development. [file PBI-14-2147-s003.pdf]

Table S1. Accumulation of individual tocopherols and tocotrienols in the seeds of oats during seed development.

	μg/g ± SE of dry weight										
	α-T[a]	α-T3[b]	β-T	β-T3	γ-T	γ-T3	δ-T	δ-T3	Total T	Total T3	Total
7-daa	3.6±0.2	4.7±0.9	0.2±0.0	0.7±0.1	0.6±0.1	0.5±0.1	0.1±0.0	0.2±0.0	4.5±0.3	6.1±1.0	10.5±1.0
14-daa	4.8±1.1	6.7±1.2	0.5±0.1	1.0±0.3	0.3±0.1	0.4±0.1	0.3±0.1	0.1±0.0	5.9±1.3	8.2±1.1	14.1±2.0
21-daa	6.8±0.4	23.0±1.9	0.8±0.1	1.9±0.2	0.3±0.1	0.4±0.1	0.1±0.0	nd	8.0±0.4	25.3±2.1	33.3±2.5
28-daa	9.2±1.2	43.5±1.0	0.7±0.0	2.8±0.1	0.2±0.1	0.2±0.0	0.1±0.0	nd	10.3±1.2	46.6±1.1	56.9±0.7

daa: days after anthesis

[a]T: Tocopherols

[b]T3: Tocotrienols

nd:non-detected

Adapted from Gutierrez-Gonzalez et al., 2013a.
