# Supplementary material for: Subgenome‐specific assembly of vitamin E biosynthesis genes and expression patterns during seed development provide insight into the evolution of oat genome
Source: Plant Biotechnol J. 2016 May 26;14(11):2147–57. doi: 10.1111/pbi.12571 (PMC5096403; doi:10.1111/pbi.12571)
Supplement: Supplementary file 10 — Table S2. Transcript normalized counts and Pearson correlation with tocol concentrations. [file PBI-14-2147-s004.pdf]

Table S2. Transcript normalized counts and Pearson correlation with tocol concentrations

	daa7			daa14			daa21			daa28			Mean				pearson		
	daa7_R1	daa7_R2	daa7_R3	daa14_R1	daa14_R2	daa14_R3	daa21_R1	daa21_R2	daa21_R3	daa28_R1	daa28_R2	daa28_R3	**daa7**	**daa14**	**daa21**	**daa28**	**Total**	**T**	**T3**
GGR_1	64.56	49.37	47.15	68.93	50.37	69.19	47.53	23.20	26.79	20.09	14.08	11.68	53.69	62.83	32.51	15.28	-0.96	-0.92	-0.97
GGR_2	84.54	47.20	85.23	108.80	87.63	90.56	58.49	56.35	63.32	55.46	70.40	58.38	72.32	95.66	59.39	61.41	-0.67	-0.59	-0.68
GGR_3	14.60	14.52	4.53	13.29	13.80	16.28	1.83	8.29	12.18	4.82	7.04	4.67	11.22	14.46	7.43	5.51	-0.89	-0.83	-0.89
GGR_4	16.91	14.52	21.76	27.41	18.63	13.23	25.59	18.23	26.79	15.27	16.09	12.45	17.73	19.75	23.54	14.60	-0.38	-0.26	-0.40
HGGT_1	216.74	211.29	217.61	235.04	239.42	201.48	219.35	195.58	226.49	131.01	140.79	157.22	215.21	225.31	213.81	143.01	-0.90	-0.83	-0.91
HGGT_2	173.70	121.98	163.21	156.14	160.77	167.90	223.01	227.07	174.13	164.76	159.90	188.36	152.96	161.60	208.07	171.01	0.41	0.50	0.40
HGGT_3	166.78	169.18	178.62	133.71	172.49	165.86	89.57	144.20	137.60	76.35	123.70	107.41	171.53	157.36	123.79	102.49	-0.98	-0.99	-0.97
HPPD_1	16.14	22.51	13.60	39.03	33.12	47.83	78.60	58.01	49.93	107.70	91.51	90.29	17.42	39.99	62.18	96.50	0.98	1.00	0.97
HPPD_2	10.76	10.89	14.51	24.92	22.08	18.32	58.49	29.83	38.97	100.46	43.24	57.60	12.05	21.77	42.43	67.10	0.99	1.00	0.99
HPPD_3	10.76	13.80	13.60	51.49	35.19	50.88	67.63	130.94	66.97	126.99	133.75	130.76	12.72	45.85	88.52	130.50	0.97	1.00	0.97
VTE1_1	94.53	70.43	100.65	75.58	96.60	92.60	60.32	71.27	75.50	49.83	59.33	49.04	88.54	88.26	69.03	52.73	-1.00	-0.97	-1.00
VTE1_2	76.09	91.49	97.02	60.63	53.13	70.21	67.63	82.87	51.14	92.43	64.36	80.17	88.20	61.32	67.22	78.99	0.05	-0.14	0.07
VTE1_3	79.16	107.46	64.38	51.49	63.48	58.00	65.81	56.35	54.80	86.00	72.41	78.61	83.67	57.66	58.99	79.01	0.16	-0.03	0.18
VTE2_1	11.53	17.43	13.60	18.27	16.56	10.18	18.28	24.86	26.79	17.68	21.12	10.90	14.19	15.00	23.31	16.57	0.37	0.45	0.36
VTE2_2	3.07	5.81	3.63	4.15	0.69	2.04	0.00	0.00	1.22	3.21	2.01	3.11	4.17	2.29	0.41	2.78	-0.29	-0.44	-0.27
VTE2_3	1.54	5.81	9.07	9.14	15.18	14.25	9.14	18.23	14.61	16.88	18.10	23.35	5.47	12.85	13.99	19.44	0.89	0.95	0.87
VTE2_4	10.76	2.18	13.60	9.97	16.56	12.21	20.11	11.60	10.96	24.11	16.09	26.46	8.85	12.91	14.22	22.22	0.96	0.96	0.95
VTE2_5	3.07	1.45	2.72	4.15	0.69	3.05	1.83	0.00	3.65	3.21	3.02	0.78	2.42	2.63	1.83	2.34	-0.37	-0.39	-0.37
VTE3_1	33.82	59.54	51.68	52.32	69.00	61.05	76.77	58.01	76.72	60.28	74.42	49.81	48.35	60.79	70.50	61.50	0.50	0.64	0.48
VTE3_2	41.50	90.76	84.33	77.24	77.97	76.32	67.63	49.72	59.67	99.66	115.65	88.73	72.20	77.17	59.01	101.35	0.61	0.54	0.62
VTE3_3	112.21	137.23	103.37	106.31	91.08	107.86	51.18	114.36	91.33	102.07	91.51	94.96	117.60	101.75	85.62	96.18	-0.61	-0.73	-0.59
VTE4_1	80.70	87.86	101.55	168.60	122.13	135.34	297.96	285.08	215.53	469.37	361.03	354.14	90.04	142.02	266.19	394.85	0.99	1.00	0.99
VTE4_2	194.45	203.31	177.72	183.54	197.33	182.14	131.61	193.92	116.90	161.55	202.14	194.58	191.82	187.67	147.48	186.09	-0.23	-0.32	-0.22
VTE4_3	140.65	167.00	160.49	117.10	100.74	138.39	100.54	92.82	76.72	75.55	80.45	92.62	156.05	118.74	90.02	82.87	-0.86	-0.94	-0.84
