# Supplementary material for: Subgenome‐specific assembly of vitamin E biosynthesis genes and expression patterns during seed development provide insight into the evolution of oat genome
Source: Plant Biotechnol J. 2016 May 26;14(11):2147–57. doi: 10.1111/pbi.12571 (PMC5096403; doi:10.1111/pbi.12571)
Supplement: Supplementary file 11 — Table S3. Vitamin E orthologous sequences used for the comparisons. [file PBI-14-2147-s005.pdf]

Table S3. Vitamin E orthologous sequences used for the comparisons.

	Barley (*H. vulgare*)	Wheat (*T. aestivum*)	*Brachypodium*	Maize (*Zea mays*)	Rice (*O. sativa*)	Sorghum (*S. bicolor*)	*A. thaliana*
HGGT	AK374688	AY222861	Bradi0007s00220		Os06g43880		
GGR	AK373508	AJ920394	Bradi3g59430	GRMZM2G105644	Os02g51080	Sb04g028050	At1g74470
HPPD	AJ000693	BT009036	Bradi3g05060	GRMZM2G088396	Os02g07160	Sb004g053700	At1g06570
VTE1	AK368882	DQ456882	Bradi3g10250	GRMZM2G009785	Os02g17650	Sb004g125800	At4g32770
VTE2	AK366699	DQ231056	Bradi1g31380	GRMZM2G048472	Os06g44840	Sb010g215600	At2g18950
VTE3	AK357314	FM998046	Bradi4g01640	GRMZM2G082998	Os12g42090	Sb008g171300	At3g63410
VTE4	AK355075	AJ920394	Bradi3g52560	GRMZM2G035213	Os02g47310	Sb004g269800	At1g64970
