# Supplementary material for: Subgenome‐specific assembly of vitamin E biosynthesis genes and expression patterns during seed development provide insight into the evolution of oat genome
Source: Plant Biotechnol J. 2016 May 26;14(11):2147–57. doi: 10.1111/pbi.12571 (PMC5096403; doi:10.1111/pbi.12571)
Supplement: Supplementary file 1 — Figure S1. Capture of reads mapped to reference barley vitamin E gene. [file PBI-14-2147-s011.pdf]

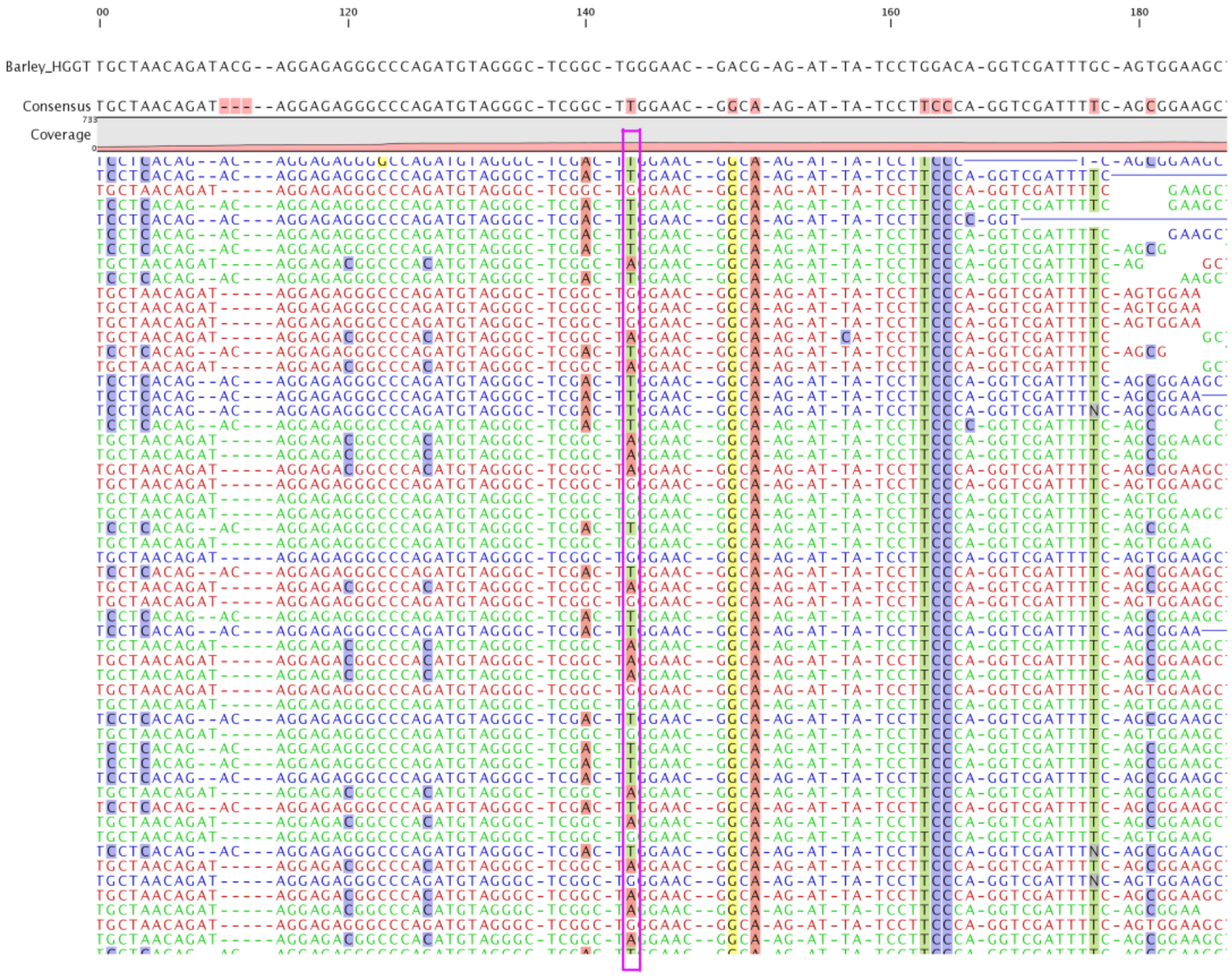

Figure S1. Capture of reads mapped to reference barley vitamin E gene. Illumina and Roche reads were mapped to vitamin E homologous genes with relaxed parameters. Mapped reads were checked for candidate allelic SNPs by visual inspection (purple box) and used in the design of allele-specific primers for PCR reactions and amplicon Sanger-sequencing. Blue reads represent mapped Illumina pairs; green reads represent mapped Illumina singletons; and red reads represent Roche-454 mapped reads.
